# Perioperative pembrolizumab, trastuzumab and FLOT in HER2-positive localized esophagogastric adenocarcinoma: a phase 2 trial

**DOI:** 10.1038/s41591-025-03979-y

**Published:** 2025-10-18

**Authors:** Alexander Stein, Eray Goekkurt, Salah-Eddin Al-Batran, Nicolas Moosmann, Thomas J. Ettrich, Thorsten Goetze, Barbara Gruen, Nils Homann, Sylvie Lorenzen, Ralf-Dieter Hofheinz, Viktor Rempel, Gabriele Siegler, Christian Müller, Benjamin Thiele, Tobias Broering, Mariana Santos Cruz, Claudia Pauligk, Mascha Binder, Joseph Tintelnot

**Affiliations:** 1 Hematology-Oncology Practice Eppendorf (HOPE), Hamburg, Germany; 2 Krankenhaus Nordwest and Institute of Clinical Cancer Research (IKF), Frankfurt, Germany; 3 Department of Medical Oncology, Barmherzige Brüder Regensburg Hospital, Regensburg, Germany; 4 l. Department of Medicine, University Hospital Ulm, Ulm, Germany; 5 Department of Medical Oncology, University Medical Center Heidelberg, Heidelberg, Germany; 6 ll. Medical Department, Klinikum Wolfsburg, Wolfsburg, Germany; 7 Department of Medicine III, TUM University Hospital, Rechts der Isar, Munich, Germany; 8 Medical Department, University Medical Center Mannheim, Mannheim, Germany; 9 Department of Gastroenterology, St. Anna Hospital Herne, Herne, Germany; 10 Department of Internal Medicine V, Hematology/Oncology, Hospital Nürnberg Nord/Paracelsus Medical University, Nürnberg, Germany; 11 Department of Hematology and Oncology, Klinik Essen-Mitte, Essen, Germany; 12 Division of Medical Oncology, University Hospital Basel, Basel, Switzerland; 13 ll. Department of Medicine, University Medical Center Hamburg-Eppendorf, Hamburg, Germany

**Keywords:** Cancer immunotherapy, Oesophageal cancer, Gastric cancer, Targeted therapies, Chemotherapy

## Abstract

Perioperative treatment strategies for HER2-positive esophagogastric adenocarcinoma remain suboptimal. Here in the open-label, phase 2 IKF/AIO PHERFLOT trial, we evaluated the safety and efficacy of adding pembrolizumab and trastuzumab to FLOT chemotherapy in patients with localized HER2-positive esophagogastric adenocarcinoma. The primary endpoints are the pathological complete response rate and the 2-year disease-free survival rate. Secondary endpoints include the R0 resection rate, feasibility and safety. Exploratory endpoints include clinical efficacy in molecularly defined subgroups. In this prespecified interim analysis, given the limited median follow-up period of 14.8 months, only one of the primary endpoints, the pathological complete response rate, and selected secondary endpoints, including the R0 resection rate, feasibility and safety, are reported here. Among 31 enrolled patients, 30 proceeded to R0 resection, and one patient declined surgery without disease progression. The combination regimen resulted in grade ≥3 treatment-related serious adverse events in 48.4% of patients (15 out of 31) aligning with established toxicity profiles of the respective agents and no treatment-related deaths. After four cycles of therapy, the pathological complete response rate was 48.4% (95% confidence interval 30.2–66.9; 15 out of 31) in the intention-to-treat population, and the subtotal regression rate (TRG1b according to Becker classification) was 19.4% (95% confidence interval 7.5–37.5; 6 out of 31), resulting in a major pathological response rate of 67.7% (95% confidence interval 48.6–83.3; 21 out of 31). Responses tended to be enriched in tumors with strong HER2 expression (immunohistochemistry 3+), high PD-L1 combined positive scores and lower T stage, but were also observed in substantial fractions of HER2 immunohistochemistry 2+/ISH+, T3 or T4 and combined positive scores <10 tumors. These findings support the feasibility and antitumor activity of perioperative chemoimmunotherapy targeting HER2 and PD-1 and warrant further validation in randomized trials. ClinicalTrials.gov registration: NCT05504720.

## Abstract (web summary)

As presented at the ESMO Congress 2025, in the single-arm phase 2 IKF/AIO PHERFLOT trial, the perioperative combination of FLOT chemotherapy with anti-PD-1 and anti-HER2 monoclonal antibodies led to a pathological complete response rate of 48.4% in patients with resectable HER2+ esophagogastric adenocarcinoma, meeting the prespecified co-primary endpoint.

## Main

Esophageal and gastric cancers rank among the most lethal malignancies globally, with approximately 1.5 million new cases and over 1 million deaths reported in 2022 ref. ^1^. For patients with resectable gastric or gastroesophageal junction adenocarcinoma, perioperative chemotherapy with the FLOT regimen—comprising of 5-fluorouracil (5-FU), leucovorin, oxaliplatin and docetaxel—has become the standard of care, supported by multiple phase 2 and 3 studies, including the pivotal FLOT4 and ESOPEC trials^2–4^.

In the metastatic setting of HER2-positive esophagogastric adenocarcinoma (EGA), the ToGA trial demonstrated that adding the HER2-targeting antibody trastuzumab to chemotherapy significantly improves survival compared to chemotherapy alone^5^. More recently, multiple trials including the pivotal KEYNOTE-811 trial demonstrated that adding pembrolizumab to fluoropyrimidine-platinum chemotherapy and trastuzumab significantly improves response, progression-free and overall survival (OS) in metastatic HER2-positive and PD-L1-positive EGA^6–8^. The MATTERHORN and DANTE studies translated this chemoimmunotherapy strategy (FLOT and PD-L1 antibody) into the perioperative setting, showing increased disease-free survival (DFS) and/or pathological complete response (pCR) rates with the addition of durvalumab or atezolizumab to FLOT^9,10^. Nonetheless, with pCR rates plateauing at 19–23% in the MATTERHON and DANTE trials, and ranging between 12.9% and 14% in other perioperative trials with chemotherapy and PD-1/PD-L1 inhibitors, further therapeutic intensification is needed to improve outcomes^11,12^. Current perioperative strategies specifically targeting the HER2-positive subpopulation of EGA are not routinely implemented. The single-arm HER-FLOT trial combined trastuzumab with FLOT, showing a pCR rate of 21.4% (ref. ^13^). Subsequently, the PETRARCA trial evaluated FLOT combined with dual HER2-targeted therapy using trastuzumab and pertuzumab, and demonstrated improved pCR rates compared to FLOT alone (35% versus 12%)^14^. On the other hand, the INNOVATION trial did not demonstrate a significant improvement in major pathological response or OS with the addition of pertuzumab to trastuzumab across different chemotherapy regimens (only 45% of patients received FLOT, while the remainder received a 5-FU/platinum doublet)^15^. Notably, the arm combining trastuzumab with chemotherapy (without pertuzumab) showed a higher major pathological response rate compared to chemotherapy alone, indicating a potential benefit and further supporting the activity of HER2-targeted therapy with trastuzumab in the perioperative setting. However, whether combining HER2-targeted therapy with chemotherapy and immunotherapy provides superior outcomes—as has been shown in metastatic combined positive score (CPS)-positive disease and suggested by preclinical evidence—remains uncertain^6–8,16–18^.

To address this unmet need, the phase 2 IKF/AIO PHERFLOT trial investigated a triple-modality approach that combined FLOT chemotherapy with the PD-1 inhibitor pembrolizumab and the HER2-targeting antibody trastuzumab in patients with localized HER2-positive EGA. The primary aim was to enhance pathological tumor regression, DFS and explore the feasibility of this intensified perioperative strategy.

## Results

### Trial design

**Fig. 1:**
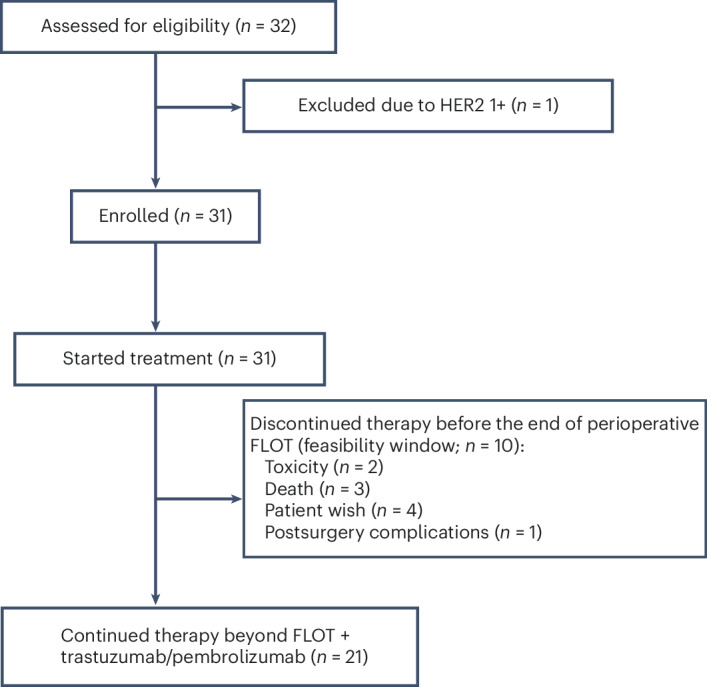
CONSORT diagram of patients included in the study. An illustration showing the number of patients screened for eligibility, reason for exclusion, the number enrolled and allocated to treatment, and those who either completed or discontinued perioperative therapy during the perioperative FLOT period, defined as the feasibility window. The enrolled cohort (*n* = 31) represents the ITT population.

From 17 March 2023 to 7 May 2024, 32 patients were screened and 31 were enrolled across 11 sites in Germany in the open-label, randomized phase 2 IKF/AIO PHERFLOT trial, comprising the intention-to-treat (ITT) population (Fig. 1). The targeted number of patients was 30; therefore, the trial over-recruited by one patient. Key inclusion criteria were nonmetastatic, resectable HER2-positive EGA—defined by immunohistochemistry (IHC) 3+ or IHC 2+ with ISH positivity—located at the gastroesophageal junction (types I–III) or stomach (cT2–4, any N or M0). Key exclusion criteria included prior exposure to immunotherapies, recent major surgery (within 2 weeks), active immunodeficiency, chronic immunosuppression exceeding 10 mg prednisone daily and uncontrolled cardiac conditions (for example, left ventricular ejection fraction < 55%). More information on the trial design, along with a full list of eligibility criteria, is available in the Methods.

**Extended Data Fig. 1:**
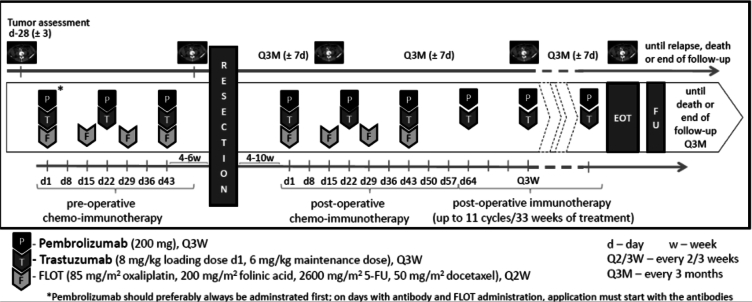
Therapy scheme. The therapy scheme is depicted, showing the timing of therapies, surgery, disease assessment, and follow-up.

Patients were scheduled to receive pembrolizumab (200 mg) and trastuzumab (initial loading dose of 8 mg kg^−1^, followed by 6 mg kg^−1^) every 3 weeks for 3 cycles before surgery. Concurrent FLOT chemotherapy included docetaxel 50 mg m^−2^, oxaliplatin 85 mg m^−2^, leucovorin 200 mg m^−2^ and a 24-hour infusion of 5-FU at 2,600 mg m^−2^, administered every 2 weeks for 4 cycles (Extended Data Fig. 1). Surgery was scheduled no earlier than 4 weeks after the final preoperative dose. Postoperatively, patients received 4 additional cycles of the same chemoimmunotherapy, followed by up to 11 cycles of pembrolizumab and trastuzumab alone, totaling 17 cycles over approximately 1 year. The primary endpoints are the pCR rate and the 2-year DFS rate. The trial aimed for a pCR rate greater than 30% and a 2-year DFS rate of over 70%. However, since this paper reports the prespecified interim analysis and the median follow-up time of 14.8 months is too short to assess the 2-year DFS rate, only the pCR rate is reported. Secondary endpoints include the R0 resection rate, overall response rate, OS, feasibility rate (defined as the proportion of patients who did not experience severe toxicity or withdrew from treatment before the final postoperative administration of FLOT plus trastuzumab and pembrolizumab, for reasons other than progressive disease) and safety. Exploratory endpoints include clinical efficacy in molecularly defined subgroups, such as CPS 0 versus 1–9 versus ≥10, HER2-3+ versus HER2-2+/ISH+ and deficient mismatch repair (dMMR) and microsatellite instability-high (MSI-high). In this prespecified interim analysis, only the R0 resection rate, feasibility safety and findings from exploratory molecular and clinical subgroups are reported as secondary endpoints.

### Patient and tumor characteristics

Overall 31 patients were recruited to the trial. Despite lack of disease progression, one patient withdrew consent for surgery during neoadjuvant therapy opting for alternative medicine instead of continuing with the protocol. Later, this patient experienced disease progression while off active therapy, thereby excluding a complete response. The median patient age was 65 years (range, 33–76). Most tumors were stage ≥T3 (67.7%) and exhibited lymph node involvement (58.1%; Table 1). All patients had HER2-positive tumors by local pathology, defined as either IHC 3+ or IHC 2+ with ISH positivity; 80.6% of cases were IHC 3+. Histologically, the majority of tumors were of intestinal type (51.6%), followed by diffuse type (9.7%) and mixed histology (3.2%). Signet ring cells were identified in 12.9% of tumors, while 64.5% were negative for signet ring features. With 19.4% of tumors being CPS-negative, the majority were CPS-positive, with 12.9% missing values.

**Table 1:** Baseline patient and tumor characteristics

Baseline factors		All patients
Age, median (range)		65 (33–76)
Sex		
	Female	6 (19.4)
	Male	25 (80.6)
ECOG	0	20 (64.5)
	1	11 (35.5)
HER2	3+	25 (80.6)
	2+/ISH+	6 (19.4)
Histology	Diffuse type	3 (9.7)
	Intestinal type	16 (51.6)
	Mixed	1 (3.2)
	Not evaluable	2 (6.5)
	NA	9 (29.0)
Signet ring cells	Yes	4 (12.9)
	No	20 (64.5)
	NA	7 (22.6)
Localization	Stomach	7 (22.6)
	AEG l	11 (35.5)
	AEG ll	10 (32.3)
	AEG lll	3 (9.7)
Barret	Yes	7 (22.6)
	No	18 (58.1)
	NA	6 (19.4)
Grading	Gx	2 (6.5)
	G1	4 (12.9)
	G2	13 (41.9)
	G3	12 (38.7)
T stage	Tx	1 (3.2)
	T1b	1 (3.2)
	T2	8 (25.8)
	T3	20 (64.5)
	T4a	1 (3.2)
N stage	Nx	3 (9.7)
	N0	10 (32.3)
	N1	18 (58.1)
CPS	0	6 (19.4)
	1–9	10 (32.3)
	≥10	11 (35.5)
	NA	4 (12.9)

Data presented as *n* with percentages in parentheses. NA, data not available; ECOG, Eastern Cooperative Oncology Group; AEG, adenocarcinoma of the esophagogastric junction; Nx, Tx and Gx, unclear status. Histology was defined according to the Lauren classification; ‘not evaluable’ indicates cases that could not be classified based on the Lauren classification.

Tumor localization included adenocarcinoma of the esophagogastric junction (AEG) type I (35.5%), AEG type II (32.3%), stomach (22.6%) and AEG type III (9.7%) cancers. Barrett’s esophagus was identified in 22.6% of cases. Tumor grading revealed G2 differentiation in 41.9%, G3 in 38.7% and G1 in 12.9%.

### Pathological response

**Fig. 2:**
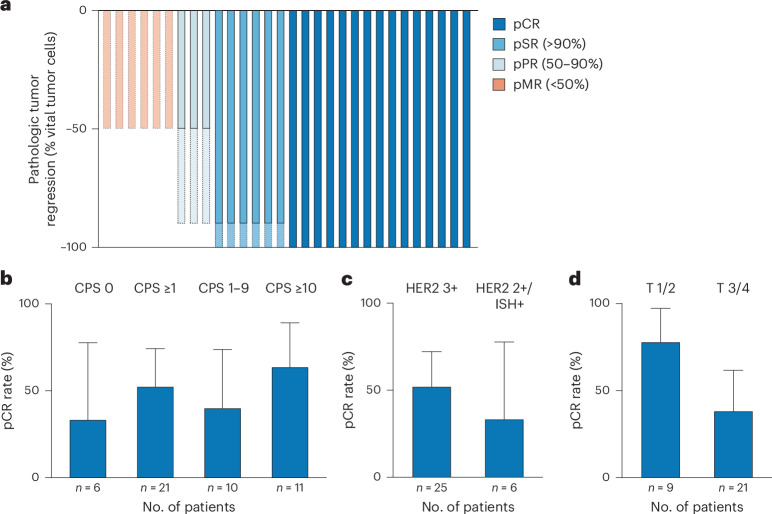
Pathological response in operated patients and in molecular and clinical subgroups. **a**, Pathological response distribution in the full cohort according to Becker classification. pCR indicates no residual tumor (TRG1a); subtotal regression (pSR, TRG1b) reflects ≥90% tumor regression; partial response (pPR, TRG2) indicates 50–90% regression; minor response (pMR, TRG3) corresponds to <50% regression. Solid segments indicate minimal regression, while dotted outlines indicate maximal estimated regression according to Becker classification. **b**, pCR rate in patients with available PD-L1 CPS data (*n* = 27), stratified by CPS 0, ≥1, 1–9 and ≥10. **c**, pCR rate stratified by HER2 expression (HER2 IHC 3+ versus HER2 2+/ISH+). **d**, pCR stratified by tumor stage (T1/2 versus T3/4); one tumor was classified as Tx (*n* = 30). The complete response rate is depicted by bars, and the upper limit of the 95% CI interval is shown.

All 30 patients who underwent surgery achieved R0 resection. pCR, defined as Becker TRG1a with no residual lymph node metastasis, was observed in 50.0% (95% CI 31.3–68.7; 15 out of 30) of patients. Subtotal regression (TRG1b) occurred in 20.0% (95% CI 7.7–38.6; 6 out of 30), while partial (TRG2) and minor responses (TRG3) were observed in 10.0% (95% CI 2.1–26.5; 3 out of 30) and 20.0% (95% CI 7.7–38.6; 6 out of 30) of cases, respectively (Fig. 2a). In the ITT population, the pCR rate was 48.4% (95% CI 30.2–66.9; 15 out of 31), subtotal regression occurred in 19.4% (95% CI 7.5–37.5; 6 out of 31), and therefore the major pathological response rate (TRG1a/b) reached 67.7% (95% CI, 48.6–83.3; 21 out of 31). Partial response was observed in 9.7% (95% CI 2.0–25.8; 3 out of 31), minor response in 19.4% (95% CI 7.5–37.5; 6 out of 31) and data were missing in 3.2% of patients (Table 2). Histopathological analysis revealed absence of lymphovascular invasion in 83.3% and no evidence of angioinvasion in 93.3% of resected specimens. Postoperative staging according to American Joint Committee on Cancer (AJCC)/Union for International Cancer Control (UICC) criteria showed tumor downstaging to stage IA or lower in 65.2% (95% CI 42.7–83.6; 15 out of 23) of gastroesophageal junction or distal esophageal tumors and 57.1% of gastric cancers (95% CI 18.4–90.1; 4 out of 7) (Extended Data Tables 1 and 2).

**Table 2:** Pathological response in the ITT population

	pCR	pSR	pPR	pMR	NA
ITT, *n* = 31	*n* = 15, 48.4% (30.2–66.9)	*n* = 6, 19.4% (7.5–37.5)	*n* = 3, 9.7% (2.0–25.8)	*n* = 6, 19.4% (7.5–37.5)	*n* = 1, 3.2%

*n*, number of patients; 95% CIs are shown in parentheses.

### Safety

All patients experienced at least one adverse event (AE) (Supplementary Information). Of these, 29 AEs were observed in more than 10% of patients (Table 3). Among the most common AEs of any grade were anorexia (32.3%), constipation (32.3%), diarrhea (83.9%), fatigue (32.3%), nausea (54.8%), decreased neutrophil count (38.7%), peripheral sensory neuropathy (80.6%), weight loss (58.1%) and decreased white blood cell count (48.4%). The most common grade ≥3 AEs were diarrhea (38.7%), decreased neutrophil count (25.8%), sepsis (19.4%) and weight loss (16.1%).

**Table 3:** AEs occurring in more than 10% of patients

National Cancer Institute Common Toxicity Criteria	Grade 1	Grade 2	Grade 3	Grade 4	Grade 5	Total
Diarrhea	5 (16.1%)	9 (29.0%)	12 (38.7%)	–	–	26 (83.9%)
Peripheral sensory neuropathy	14 (45.2%)	9 (29.0%)	2 (6.5%)	–	–	25 (80.6%)
Weight loss	3 (9.7%)	10 (32.3%)	5 (16.1%)	–	–	18 (58.1%)
Nausea	5 (16.1%)	10 (32.3%)	2 (6.5%)	–	–	17 (54.8%)
White blood cell decrease	5 (16.1%)	6 (19.4%)	4 (12.9%)	–	–	15 (48.4%)
Neutrophil count decrease	1 (3.2%)	3 (9.7%)	5 (16.1%)	3 (9.7%)	–	12 (38.7%)
Anorexia	4 (12.9%)	5 (16.1%)	1 (3.2%)	–	–	10 (32.3%)
Constipation	5 (16.1%)	5 (16.1%)	–	–	–	10 (32.3%)
Fatigue	4 (12.9%)	5 (16.1%)	1 (3.2%)	–	–	10 (32.3%)
Anemia	2 (6.5%)	3 (9.7%)	4 (12.9%)	–	–	9 (29.0%)
Fever	4 (12.9%)	3 (9.7%)	2 (6.5%)	–	–	9 (29.0%)
Mucositis oral	6 (19.4%)	2 (6.5%)	1 (3.2%)	–	–	9 (29.0%)
Alopecia	3 (9.7%)	5 (16.1%)	–	–	–	8 (25.8%)
Vomiting	3 (9.7%)	3 (9.7%)	2 (6.5%)	–	–	8 (25.8%)
Hypokalemia	2 (6.5%)	3 (9.7%)	2 (6.5%)	–	–	7 (22.6%)
Pain	4 (12.9%)	3 (9.7%)	–	–	–	7 (22.6%)
Aspartate aminotransferase increase	6 (19.4%)	–	–	–	–	6 (19.4%)
Sepsis	–	–	4 (12.9%)	1 (3.2%)	1 (3.2%)	6 (19.4%)
Alanine aminotransferase increase	3 (9.7%)	2 (6.5%)	–	–	–	5 (16.1%)
Dysgeusia	3 (9.7%)	2 (6.5%)	–	–	–	5 (16.1%)
Dysphagia	1 (3.2%)	1 (3.2%)	3 (9.7%)	–	–	5 (16.1%)
Cough	2 (6.5%)	2 (6.5%)	–	–	–	4 (12.9%)
Dyspnea	2 (6.5%)	–	1 (3.2%)	1 (3.2%)	–	4 (12.9%)
Eczema	3 (9.7%)	1 (3.2%)	–	–	–	4 (12.9%)
Hypothyroidism	1 (3.2%)	3 (9.7%)	–	–	–	4 (12.9%)
Lipase increased	1 (3.2%)	1 (3.2%)	2 (6.5%)	–	–	4 (12.9%)
Platelet count decrease	3 (9.7%)	–	1 (3.2%)	–	–	4 (12.9%)
Pruritus	3 (9.7%)	–	1 (3.2%)	–	–	4 (12.9%)
Rash acneiform	2 (6.5%)	2 (6.5%)	–	–	–	4 (12.9%)

Data presented as *n* with percentages in parentheses. AEs are reported according to Common Terminology Criteria for Adverse Events. The table reflects the highest grade per patient.

Grade ≥3 treatment-related serious adverse events (SAEs) occurred in 48.4% of patients. Treatment-related SAEs were attributed to pembrolizumab in 32.3% of patients, trastuzumab in 22.6%, oxaliplatin in 41.9%, 5-FU in 48.4%, folinic acid in 29.0% and docetaxel in 41.9% (Extended Data Table 3). The most common grade ≥3 treatment-related AEs were diarrhea, which was attributed to FLOT in 83.3% (10 out of 12) of cases and to pembrolizumab and trastuzumab in 50.0% (6 out of 12); decreased neutrophil count, which was attributed to FLOT in 100% (8 out of 8) of cases and not to pembrolizumab or trastuzumab; and decreased white blood cell count, which was attributed to FLOT in 100% (4 out of 4) of cases and to pembrolizumab and trastuzumab in 50.0% (2 out of 4). No immune-related AEs of special interest or other clinically notable events were observed. Two patients experienced fatal SAEs, both unrelated to the therapy: one died from hyperglycemia and sepsis within 60 days postoperatively, and the other from acute respiratory distress syndrome approximately 3.5 months after surgery (Extended Data Table 4). Moreover, one patient died due to the underlying disease outside the SAE reporting period; the event was unrelated to treatment. Overall, only two patients discontinued therapy due to toxicity during the perioperative FLOT period, defined as the feasibility window. In addition, three patients discontinued due to death, four due to patient preference and one due to postsurgical complications, resulting in a positive feasibility outcome (aimed feasibility rate ≥0.66; see Fig. 1).

Surgery was performed in 30 patients. The mean time to surgery was 88.9 days after enrollment (range, 72–111 days; Extended Data Table 5). The per-protocol window of 4–6 weeks posttreatment was met in 93.3% of cases (28 out of 30). Surgery was complication-free in 70.0% of patients (21 out of 30). Surgical and medical complications were observed in four patients each and one patient showed both surgical and medical complications. Surgical complications included postoperative hemorrhage (*n* = 1), anastomotic leakage (*n* = 2), fistula (*n* = 1), herniation (*n* = 1) and conduit ischemia (*n* = 1) (Extended Data Table 6). Medical complications included pneumonia (*n* = 1), sepsis (*n* = 1), cardiovascular and respiratory failure with acute respiratory distress syndrome (*n* = 1), renal failure (*n* = 1) and other events such as pleural effusion, hemorrhagic shock, staphylococcal infection, worsening of sleep apnea with delirium and peripheral pulmonary embolism (Extended Data Table 7). Overall, eight patients required reoperation (26.7%). The median inpatient stay was 14 days (range, 8–113). No patient died within 30 days postsurgery, but one patient died within 60 days after surgery as described.

### Tumor subgroups

PD-L1 expression, measured by the CPS, is a validated biomarker for predicting response to immune checkpoint inhibitors in EGA^19–21^. Similarly, HER2 status serves as a predictive marker for response to HER2-targeted therapies such as trastuzumab^19,22,23^. CPS data were available for 27 patients. Complete and subtotal responses were observed across CPS 0, CPS 1–9 and CPS ≥ 10 subgroups (Fig. 2b and Table 4). In the group of CPS negative tumors, 33.3% (95% CI 4.3–77.7; 2 out of 6) had a pCR, 50.0% (95% CI 11.8–88.2; 3 out of 6) had a subtotal response and 16.7% (95% CI 0.4–64.1; 1 out of 6) had a partial response (Table 4). In the group of CPS ≥ 1 tumors, 52.4% (95% CI 29.8–74.3; 11 out of 21) had a pCR, 14.3% (95% CI 3.0–36.3; 3 out of 21) had a subtotal response, 28.6% (95% CI 11.3–52.2; 6 out of 21) had a minor response and 4.8% (1 out of 21) did not undergo surgery. In the group of CPS 1–9 tumors, 40.0% (95% CI 12.2–73.8; 4 out of 10) had a pCR, 50.0% (95% CI 18.7–81.3; 5 out of 10) had a minor response and 10.0% (1 out of 10) did not undergo surgery. In the group of CPS ≥ 10 tumors, 63.6% (95% CI 30.8–89.1; 7 out of 11) had a pCR, 27.3% (95% CI 6.0–61.0; 3 out of 11) had a subtotal regression and 9.1% (95% CI 0.2–41.3; 1 out of 11) had a minor response.

**Table 4:** Pathological response in the ITT population based on CPS scores of 0, ≥1, 1–9 or ≥10; HER2 3+ or HER2 2+/ISH+; T1/2 or T3/4 or dMMR/MSI

	*n*	pCR	pSR	pPR	pMR	NA
CPS 0	6	33.3% (4.3–77.7)	50.0% (11.8–88.2)	16.7% (0.4–64.1)	–	–
CPS ≥ 1	21	52.4% (29.8–74.3)	14.3% (3.0–36.3)	–	28.6% (11.3–52.2)	4.8%
CPS 1–9	10	40.0% (12.2–73.8)	–	–	50.0% (18.7–81.3)	10.0%
CPS ≥ 10	11	63.6% (30.8–89.1)	27.3% (6.0–61.0)	–	9.1% (0.2–41.3)	–
HER2 3+	25	52.0% (31.3–72.2)	16.0% (4.5–36.1)	12.0% (2.5–31.2)	16.0% (4.5–36.1)	4.0%
HER2 2+/ISH+	6	33.3% (4.3–77.7)	33.3% (4.3–77.7)	–	33.3% (4.3–77.7)	–
T1/2	9	77.8% (40.0–97.2)	–	–	11.1% (0.3–48.2)	11.1%
T3/4	21	38.1% (18.1–61.6)	28.6% (11.3–52.2)	9.5% (1.2–30.4)	23.8% (8.2–47.2)	–
dMMR/MSI	3	100% (29.2–100)	–	–	–	–

*n*, number of patients; percentages and 95% CIs are shown in parentheses.

Both HER2 IHC 3+ and IHC 2+ with ISH amplification tumors were eligible for inclusion. Among patients with HER2 IHC 3+ expression, the pCR rate was 52.0% (95% CI 31.3–72.2; 13 out of 25), compared to 33.3% (95% CI 4.3–77.7; 2 out of 6) in the HER2 IHC 2+/ISH+ subgroup (Fig. 2c and Table 4). Among patients with lower-stage tumors (T1/2), 77.8% (95% CI 40.0–97.2; 7 out of 9) achieved a complete response, while 11.1% (95% CI 0.3–48.2; 1 out of 9) had a minor response and one declined surgery (Fig. 2d and Table 4). In contrast, among those with T3 or T4 tumors, the pCR rate was 38.1% (95% CI 18.1–61.6; 8 out of 21) and subtotal regression occurred in 28.6% (95% CI 11.3–52.2; 6 out of 21), resulting in a combined major regression rate of 66.7% (95% CI 43.0–85.4; 14 out of 21) in large tumors.

MMR/MSI-status, tested via PCR or IHC, was available for 29 patients; three of these were classified as dMMR by IHC. One of them was MSI stable by PCR, while PCR results were unavailable for the other two. Notably, all three patients with dMMR achieved complete pathological responses.

## Discussion

Neoadjuvant immunotherapy has reshaped the management of different cancers, with the most notable success seen in dMMR/MSI-high localized rectal cancer, where remarkable complete response rates have enabled cure without the need for surgery^24–26^. In EGA, the phase 3 MATTERHORN trial demonstrated that adding the PD-L1 inhibitor durvalumab to FLOT chemotherapy increased the pCR rate from 7.2% to 19.2% in resectable EGA, suggesting a shift in future standards of care^10^. Other chemoimmunotherapy trials in the perioperative setting have typically reported pCR rates ranging between 12.9% and 14%, suggesting that further treatment intensification may be needed to improve outcomes^11,12^. Meanwhile, HER2-targeted therapy with trastuzumab has improved outcomes in metastatic HER2-positive EGA, and the KEYNOTE-811 trial showed that adding pembrolizumab to trastuzumab and chemotherapy significantly improved objective response rate, progression-free survival and OS in CPS-positive cases^5–7^.

In this context, the PHERFLOT trial evaluated an intensified perioperative regimen combining FLOT with pembrolizumab and trastuzumab in patients with localized HER2-positive disease. We observed a pCR rate of 48.4% in the ITT population and 50.0% among resected patients, both exceeding the expected pCR rate of 30.0%, which was defined as one of the trial’s two primary endpoints. An additional 19.4% achieved subtotal regression, particularly in T3 and T4 tumors, suggesting that increasing neoadjuvant intensity may lead to deeper responses.

These outcomes compare favorably with historical data: 15% pCR in FLOT4 ref. ^2^, 19% in MATTERHORN^10^, 23% in the DANTE trial^9^ and up to 35% in HER2-targeted regimens such as PETRARCA^14^. In a recent phase 2 trial, capecitabine and oxaliplatin were combined with trastuzumab ± atezolizumab, yielding pCR rates of 14–38% (*n* = 3 or 8)^27^. The comparable patient populations suggest that the more intensive FLOT backbone used in PHERFLOT may induce superior responses. Similar indications are emerging from other trials, such as KEYNOTE-585, which used chemotherapy backbones more frequently than FLOT and showed negative results for the combination of perioperative chemotherapy and immunotherapy^11^. These findings suggest that the choice of chemotherapy backbone can influence outcomes, further supporting the global use of FLOT.

Whether the improved pCR rate of the PHERFLOT regimen translates into improved DFS remains to be proven, as the median follow-up time is still too short to assess the second primary endpoint of the 2-year DFS rate. If proven successful, pCR rates of around 50%—along with an additional 20% showing subtotal regression and continued therapy planned for up to 1 year—should prompt a discussion about organ-preserving approaches, similar to those established for dMMR/MSI-high rectal cancer^24–26^. In EGA, complete or major pathological response has been associated with longer DFS and OS in real-world data for FLOT, docetaxel-based perioperative chemotherapy and FLOT combined with the PD-1 inhibitor toripalimab^28–30^. However, the definition of complete pathological response in the absence of a resection in EGA is complicated by falsely-negative endoscopic and biopsy findings, as well as falsely-positive and negative positron emission tomography–computed tomography scans^31,32^. Nonetheless, current trials are investigating nonoperative approaches in esophageal cancer, demonstrating the feasibility of accurately assessing clinical complete remission through repeated evaluations^33,34^. Furthermore, a nonoperative approach may be more feasible in a cohort with a particularly high likelihood of achieving pCR. Our data suggest that this could apply to at least 50–70% of patients with HER2-positive EGA treated with FLOT, trastuzumab and the PD-1 inhibitor pembrolizumab, particularly if treatment is continued after the neoadjuvant phase with four additional cycles of FLOT plus trastuzumab and a PD-1 inhibitor in cases of major local remission indicative of a later clinical complete response.

The safety profile of PHERFLOT was consistent with expectations. Most adverse events were attributable to chemotherapy. Grade ≥3 events such as neutropenia (25.8%), nausea (6.5%) and febrile neutropenia (3.2%) occurred at lower rates than those reported in FLOT4 ref. ^2^. In contrast, grade 3 diarrhea increased to 38.7%, compared to around 6% in trials of FLOT alone or FLOT plus durvalumab^2,4,10^. Most cases of diarrhea were attributed to FLOT (83%), while only 50% were considered related to pembrolizumab or trastuzumab. All three agents are known to potentially cause diarrhea^2,35,36^. In the ToGA trial, the addition of trastuzumab increased the rate of grade ≥3 diarrhea from 4% to 8%^5^. Similarly, in the PRODIGE 51–FFCD-GASTFOX trial, the addition of docetaxel raised the rate of grade ≥3 diarrhea from 7% to 15% (ref. ^37^). In contrast, the addition of checkpoint inhibitors did not increase the rate of perioperative diarrhea in either KEYNOTE-585 or MATTERHORN^10,11^. However, combining FLOT with pertuzumab and trastuzumab—that is, dual HER2 blockade—markedly increased the rate of grade ≥3 diarrhea to 41%^38^. Therefore, it is likely that the combination of chemotherapy including docetaxel and trastuzumab accounts for the observed increase in grade 3 diarrhea. Still, we cannot exclude that the addition of pembrolizumab also contributed to the incidence of diarrhea when combined with FLOT and trastuzumab. Overall, only two patients discontinued treatment during the perioperative feasibility window due to toxicity. In addition, three patients discontinued due to death, four due to patient preference and one due to postsurgical complications. Although the reasons for patient preference were not disclosed, even when all such events were counted as nonfeasible, the feasibility rate still met the target of ≥0.66, leading to a positive conclusion regarding the feasibility of the PHERFLOT regimen.

Two patients experienced fatal SAEs, neither treatment-related. Another patient died due to the underlying disease outside the SAE reporting period; again, the event was unrelated to treatment. Postoperative complications occurred in nine patients, and eight required reoperation (26.7%) including two due to anastomotic leakage, which is substantially higher compared to FLOT4 (10%). However, the median hospital stay was 14 days (range, 8–113), and was therefore comparable to the 15 days observed in FLOT4. Importantly, no 30-day postoperative deaths occurred. Although the sample size in this trial does not permit a direct comparison to complication rates in FLOT4, no signal of increased early postoperative mortality was observed; however, the reoperation rate was higher as detailed. The reason for the observed increase in surgical morbidity cannot be conclusively determined. Targeting HER2 in the perioperative setting has not been associated with increased surgical mortality in either the PETRARCA or HER-FLOT trials^13,14,38^. In the HER-FLOT trial, a numerical increase in anastomotic leakage was observed but not in overall morbidity^13^. This was presumed to be due to the higher proportion of esophageal cancers included in the trial. Of note, the distribution of AEG types I–III and gastric cancers was comparable between PHERFLOT and HER-FLOT. Regarding FLOT combined with PD-1/PD-L1 inhibition, the MATTERHORN trial has not yet reported detailed surgical morbidity; however, the absence of increased delays in initiating adjuvant therapy suggests no substantial rise in perioperative morbidity^10^. Therefore, it remains unclear whether the combination of HER2-targeted therapy and immunotherapy, differences in patient composition compared to FLOT4 or the small sample size with possible oversampling contributed to this observation. The possibility of increased perioperative morbidity, together with the higher incidence of diarrhea, although not exceeding grade 3 severity, raises the question of whether de-escalation strategies should be explored in patients achieving a pCR. This may be particularly important in molecular subgroups such as patients who are dMMR/MSI-high. In our trial, all three patients with dMMR tumors achieved a pCR (100%), a rate that appears higher than those reported in other studies: 63% in the DANTE trial and a 38% higher rate in KEYNOTE-585 for patients with dMMR/MSI-high treated with chemoimmunotherapy^9,11,39^. However, the number of patients with dMMR/MSI-high in these studies is small, limiting definitive conclusions. Nevertheless, these findings suggest that adding trastuzumab may further enhance pCR rates even in this highly immunotherapy-responsive subgroup, or that the subset of HER2-positive dMMR/MSI-high EGA exhibits increased overall immunotherapy responsiveness^40^.

Exploratory analyses suggested biological activity across patient subgroups. Patients with a PD-L1 CPS ≥ 10 showed higher pCR rates (63.6%) than those with CPS 1–9 (40.0%), though responses were observed in both groups. Of note, a pCR rate of 33.3% (with one of the two cases being dMMR) and a subtotal regression rate of 50% (with all cases being MMR proficient) suggest activity in PD-L1-negative patients. This contrasts with the current approval of pembrolizumab and trastuzumab in combination with chemotherapy for CPS ≥ 1 metastatic HER2-positive EGA, which was based on KEYNOTE-811 data^6,7^. Similarly, HER2 IHC 3+ tumors had higher pCR rates (52.0%) than IHC 2+/ISH+ tumors (33.3%). These findings suggest that both the PD-1 inhibitor pembrolizumab and the HER2-targeted antibody trastuzumab may contribute to the high pCR rates observed in this cohort. Among early-stage tumors (T1 and T2), the pCR rate reached 77.8%; in T3 and T4 tumors, the combined pCR and subtotal regression rate was 66.7%. The ratio of pCR to subtotal regression in larger tumors compared to smaller ones suggests that extended neoadjuvant therapy may further increase pCR rates in the T3 and T4 group.

Limitations of the PHERFLOT trial include its single-arm design and its conduct in a single country, potentially limiting generalizability. Moreover, a limitation of this publication is the absence of mature DFS data—a co-primary endpoint. Nevertheless, this regimen demonstrated remarkable efficacy, yielding higher pCR rates than those reported for the current standard of care. However, these outstanding outcomes may be offset by increased perioperative morbidity. Accordingly, future trials of this or similar protocols should evaluate whether organ preservation strategies might be appropriate for patients who achieve complete responses and/or whether other therapy de-escalation strategies might be feasible for these patients.

Overall, the PHERFLOT trial demonstrates that perioperative FLOT combined with pembrolizumab and trastuzumab is safe and feasible, and induces high pathological response rates in localized HER2-positive EGA. Among operated patients, the pCR rate was 50%, and at least subtotal regression was observed in 70%, demonstrating superior efficacy of this regimen compared with current standards. Pending longer-term survival data, PHERFLOT provides a strong rationale for further investigation in larger randomized trials.

## Methods

### Trial design and recruitment

The PHERFLOT study is an open-label, single-arm, multicenter exploratory phase 2 trial that investigates perioperative pembrolizumab, trastuzumab and FLOT in patients with HER2-positive, localized esophagogastric adenocarcinoma. Between 17 March 2023 and 7 May 2024, 31 patients were enrolled across 11 German cancer centers. The trial is NCT05504720 (clinicaltrials.gov) and 2024-513610-34-00 (euclinicaltrials.eu) registered.

### Participant eligibility and inclusion criteria

Participants were eligible for enrollment if all of the following criteria were met and written informed consent was obtained before any study-specific procedures. Participants were adults (≥18 years) of any sex. Patients self-reported their sex and ethnicity; no information on gender was collected. There were no sex-specific enrollment restrictions. Participants were required to be able and willing to comply with all protocol procedures, including surgery, based on the investigator’s judgment. Eligible participants had histologically confirmed adenocarcinoma of the stomach or gastroesophageal junction (Siewert Type I–III/AEG l–lll), staged as cT2–4, any N, M0, or any T, N+ or M0. Tumors had to be medically and technically resectable, without invasion of adjacent structures, and with no signs of peritoneal carcinomatosis or distant metastases. Absence of distant metastasis was confirmed via thoracoabdominal CT or MRI. If clinically suspected, bone metastases were excluded with bone scintigraphy. Laparoscopy was required in patients with T3 or T4 tumors of diffuse histology or suspected peritoneal disease. Tumors were HER2-positive, defined as IHC 3+ or IHC 2+ with ISH+, based on certified local testing of the primary tumor. All patients were candidates for curative-intent resection, had not received prior systemic anticancer therapy or surgical resection of the primary tumor, and had an ECOG performance status of 0 or 1. Male participants agreed to use contraception during treatment and for 6 months afterward. Female participants were either not of childbearing potential or agreed to use contraception for at least 7 months following the final dose. Pregnant or breastfeeding individuals were excluded.

Adequate organ function was defined by the following laboratory criteria: an absolute neutrophil count of ≥1,500 per μl, leukocyte count of ≥3,000 per μl, platelet count of ≥100,000 per μl, and hemoglobin level of ≥9.0 g dl^−1^ or ≥ 5.6 mmol l^−1^ without recent transfusion or erythropoietin support. Renal function had to be preserved, with a creatinine clearance of ≥ 50 ml min^−1^, either measured or calculated per institutional standards. Hepatic function criteria included a total bilirubin of ≤1.5 times the upper limit of normal, or direct bilirubin within normal limits in patients with elevated total bilirubin, and transaminases (aspartate aminotransferase and alanine aminotransferase) ≤ 2.5 ⨉ upper limit of normal. Coagulation parameters had to be within acceptable limits, with an international normalized ratio or prothrombin time, and activated partial thromboplastin time ≤1.5 ⨉ upper limit of normal, unless the participant was receiving therapeutic anticoagulation.

### Exclusion criteria

Participants were excluded from the study if any of the following conditions were present: receipt of a live or live-attenuated vaccine within 30 days before the first dose of study treatment (administration of killed vaccines was permitted); current or recent (within 4 weeks or 5 half-lives, whichever is longer) participation in another clinical trial involving an investigational agent or device; a diagnosis of immunodeficiency or receipt of chronic systemic corticosteroids exceeding 10 mg of prednisone equivalent per day, or other immunosuppressive therapies, within 7 days before the first dose; an active second malignancy requiring treatment within the past 2 years (exceptions included adequately treated basal cell or squamous cell skin cancer or carcinoma in situ); diagnosis or suspicion of myelodysplastic syndrome or acute myeloid leukemia; severe dyspnea at rest requiring supplemental oxygen; history of severe allergic or anaphylactic reactions to chimeric or humanized antibodies or fusion proteins; known hypersensitivity to Chinese hamster ovary cell products, pembrolizumab or trastuzumab; any contraindication or hypersensitivity to components of the chemotherapy backbone (docetaxel, 5-FU, leucovorin or oxaliplatin) or known dihydropyrimidine dehydrogenase deficiency. Patients with reduced dihydropyrimidine dehydrogenase activity (Clinical Pharmacogenetics Implementation Consortium score 1.0–1.5) could be enrolled with dose adjustment following discussion with the sponsor. Other exclusion criteria included: active autoimmune disease requiring systemic treatment in the past 2 years (hormone replacement therapy was allowed); history of noninfectious pneumonitis/interstitial lung disease requiring steroids, or current interstitial lung disease; active infection requiring systemic treatment; known history of HIV infection; known active hepatitis B (HBsAg positive) or hepatitis C infection (detectable HCV RNA); any serious or uncontrolled medical condition making the participant an unsuitable candidate for the trial (for example, uncontrolled arrhythmias, recent myocardial infarction, severe psychiatric illness, unstable spinal cord compression, superior vena cava syndrome, extensive interstitial lung disease on high-resolution computed tomography, prior allogeneic stem cell or organ transplant); and pregnancy or breastfeeding. Participants unwilling or unable to use contraception or those planning to conceive or father a child during the study or within 6 months after its completion were also excluded.

### Protocol amendments and safety monitoring board

The first protocol version, V1.2, was approved on 11 October 2022 by the independent ethics committee of the medical council Hamburg. A single protocol amendment was made on 14 March 2024. This amendment included a change in the sponsor’s name, updates to the protocol in accordance with the new Clinical Trial Regulation and the transition of all clinical trials to the Clinical Trials Information System portal in Europe, and the removal of a minimum hemoglobin threshold for the administration of study drugs, as this was deemed unnecessary by the investigators. In addition, the amendment clarified that all patients who received at least one dose of the study treatment would be considered evaluable for safety and included in the safety population. The amendment also included formal corrections and adjustments. Safety parameters, including SAEs, were monitored in near real time by a continuous toxicity monitoring board for the first six patients enrolled, to promptly identify any potential safety risks. Furthermore, the toxicity monitoring board convened after these six patients had completed the third treatment cycle with pembrolizumab, trastuzumab and FLOT, and had passed their presurgical assessment. The purpose of this meeting was to reevaluate the risk–benefit ratio of the trial and to provide a recommendation on its continuation to the coordinating investigator and the sponsor. Following this initial phase, the safety monitoring board continued to oversee the study through two semiannual and two annual safety reports.

### Treatment regimen

Patients received pembrolizumab (200 mg) and trastuzumab (initial loading dose of 8 mg kg^−1^, followed by 6 mg kg^−1^) every 3 weeks. Concurrent FLOT chemotherapy comprised docetaxel 50 mg m^−2^, oxaliplatin 85 mg m^−2^, leucovorin 200 mg m^−2^ and a 24-hour infusion of 5FU at 2,600 mg m^−2^ every 2 weeks for 4 cycles. Surgery was scheduled not earlier than 4 weeks after the final preoperative dose. Postoperatively, patients underwent 4 additional cycles of the same chemoimmunotherapy, followed by up to 11 cycles of pembrolizumab and trastuzumab alone, totaling 17 cycles over approximately 1 year.

### Statistical analysis

The study’s co-primary endpoints are the 2-year disease-free survival rate (DFSR@2) and pCR rate. For the 2-year disease-free survival rate, the null hypothesis of *P* ≤ 50% against the alternative of *P* ≥ 70% will be tested using a 1-sided *α* of 0.10 and 80% power in a Fleming single-stage design. For the pCR rate, the null hypothesis of *P* ≤ 12% versus an alternative of *P* ≥ 30% was tested with a 1-sided *α* of 0.05 and 80% power. Both calculations required 27 evaluable patients; accounting for a 10% dropout rate, the planned enrollment was set at 30 patients. Secondary endpoints including overall response, R0 resection, OS, Becker regression grading (TRG1a/b), perioperative morbidity and mortality, feasibility rate and safety (AEs per NCI CTC v5.0) are analyzed descriptively, with time-to-event outcomes estimated by Kaplan–Meier methods. Calculations were performed on the data actually available. Incomplete time-to-event observations were handled as censored measurements, and missing data for the primary endpoint were considered failures. In this interim analysis, only the pCR rate is reported because the 2-year disease-free survival rate data are not yet mature. Regarding the secondary endpoints, the R0 resection rate, safety and toxicity, and feasibility are reported, as survival data are still immature and overall response rate data are not yet available. The trial protocol and statistical analysis plan are available in the Supplementary Information. The CIs for pathological response rates were calculated using the Clopper–Pearson method. CIs were calculated using Prism, version 9.5.1. For reporting of the other endpoint data, SAS software version 9.4 or higher (SAS Institute Inc.) or R version 3.6.1 or higher (R Foundation for Statistical Computing) was used.

### Pathology and biomarker assessment

Local pathologists determined pCR and tumor regression grades according to Becker, with central report review for confirmation. PD-L1 expression as CPS, dMMR/MSI and HER2 status (IHC/ISH) were assessed locally on baseline tumor specimens in most cases. In seven cases, PD-L1 and dMMR were tested centrally due to missing local information. At local sites, routine clinical testing followed established pathology protocols. For cases requiring central testing, the following standardized protocol was applied. IHC was performed on 2-µm paraffin sections using the Ventana Benchmark XT automated staining system (Ventana Medical Systems). The following antibodies were used at the manufacturer’s ready-to-use concentrations: PD-L1 (Ventana, clone SP263; catalog no. 790-4905), MLH1 (Ventana, clone M1; catalog no. 760-5091), PMS2 (Ventana, clone A16-4; catalog no. 760-5094), MSH2 (Ventana, clone G219-1129; catalog no. 760-5093) and MSH6 (Ventana, clone SP93; catalog no. 760-5092). Tumors were considered MMR proficient if MLH1, PMS2, MSH2 and MSH6 all demonstrated nuclear staining in tumor cells. CPS was calculated as the number of PD-L1-stained tumor cells, lymphocytes and macrophages divided by the total number of viable tumor cells, multiplied by 100. CPS = 0 indicated no PD-L1-positive cells, CPS 1–9 indicated low PD-L1 expression and CPS ≥ 10 indicated high PD-L1 expression. All available results were reported at the time of submission. Missing data are indicated.

### Reporting summary

Further information on research design is available in the Nature Portfolio Reporting Summary linked to this article.

## Online content

Any methods, additional references, Nature Portfolio reporting summaries, source data, extended data, supplementary information, acknowledgements, peer review information; details of author contributions and competing interests; and statements of data and code availability are available at 10.1038/s41591-025-03979-y.

## Supplementary information

Supplementary Information Supplementary Table 1 (all adverse events), Study Protocol and Statistical Analysis Plan.

Reporting Summary

## Figures and Tables

**Extended Data Table 1:**
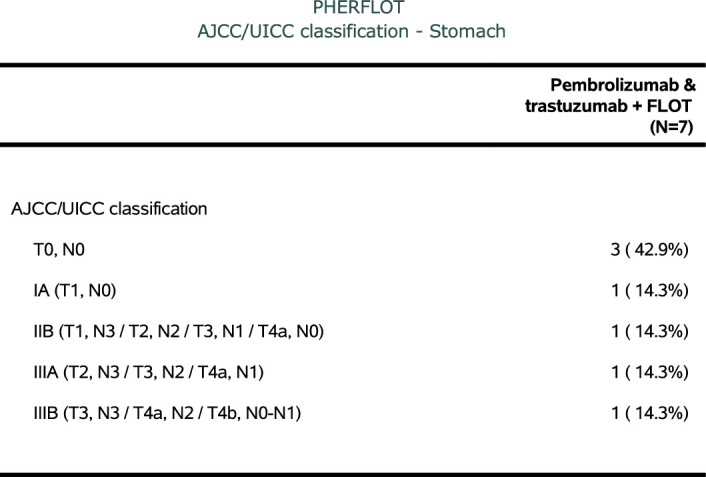
AJCC/UICC response

AJCC/UICC classification for stomach cancer, based on post-neoadjuvant status in the resected specimen. Number of patients and percentages are indicated.

**Extended Data Table 2:**
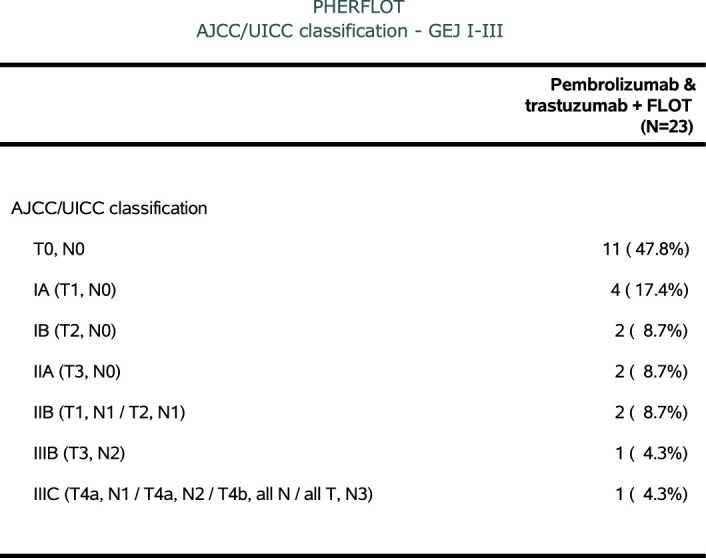
AJCC/UICC response

AJCC/UICC classification for GEJ (types I–III) cancer, based on post-neoadjuvant status in the resected specimen. Number of patients and percentages are indicated.

**Extended Data Table 3:**
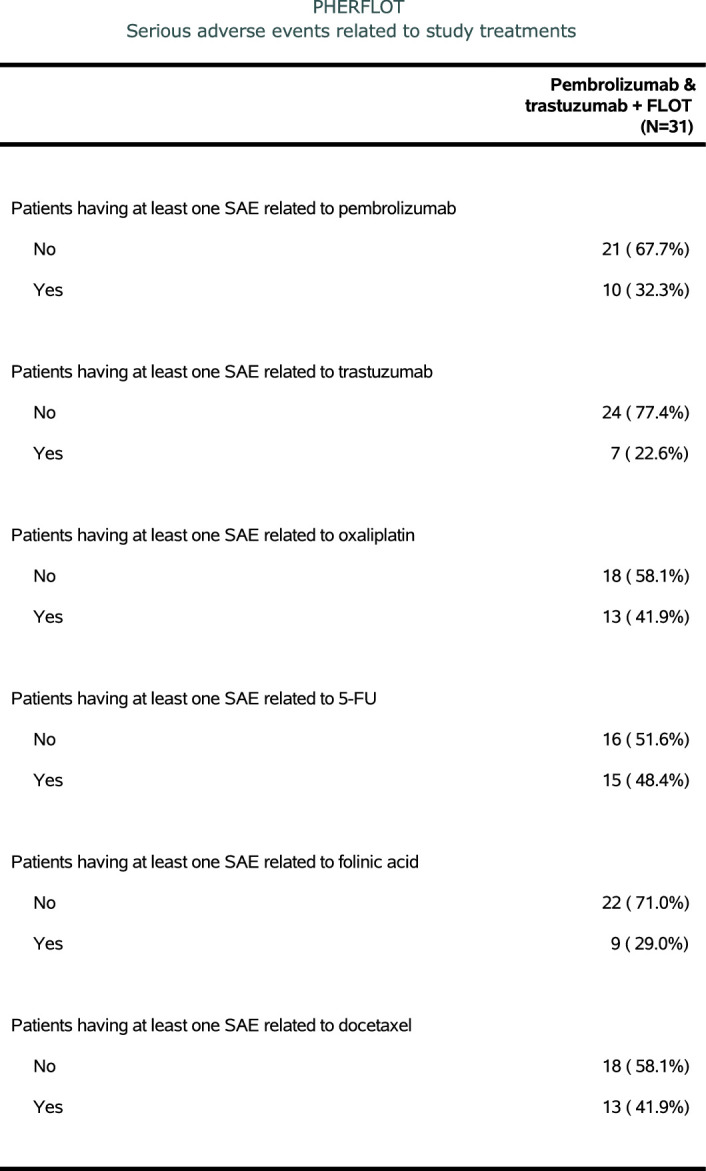
SAEs by study treatment

SAEs related to study treatments are indicated. Number of patients and percentages are indicated.

**Extended Data Table 4:**
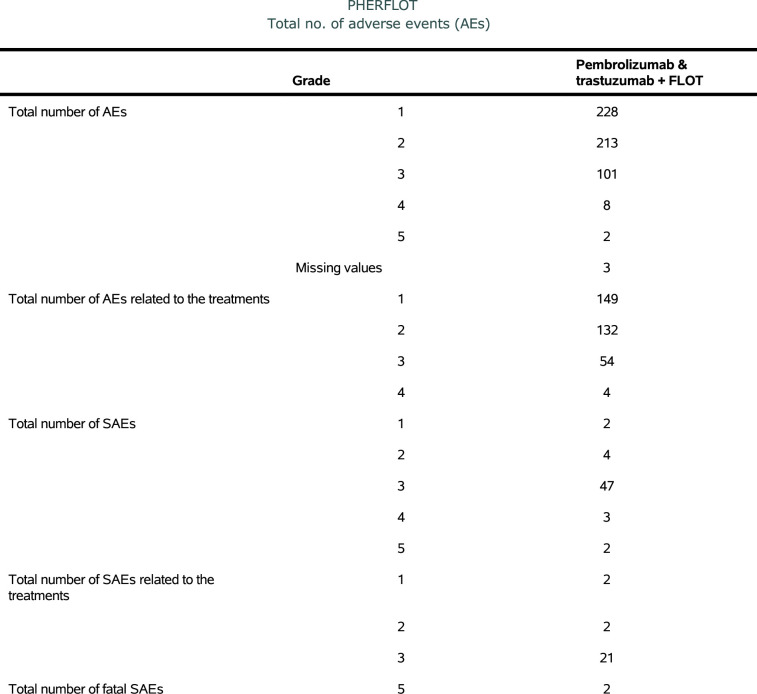
Number of SAEs

The numbers of AEs, treatment-related AEs, serious AEs (SAEs), treatment-related SAEs, and fatal SAEs are reported. Patient numbers and percentages are indicated.

**Extended Data Table 5:**
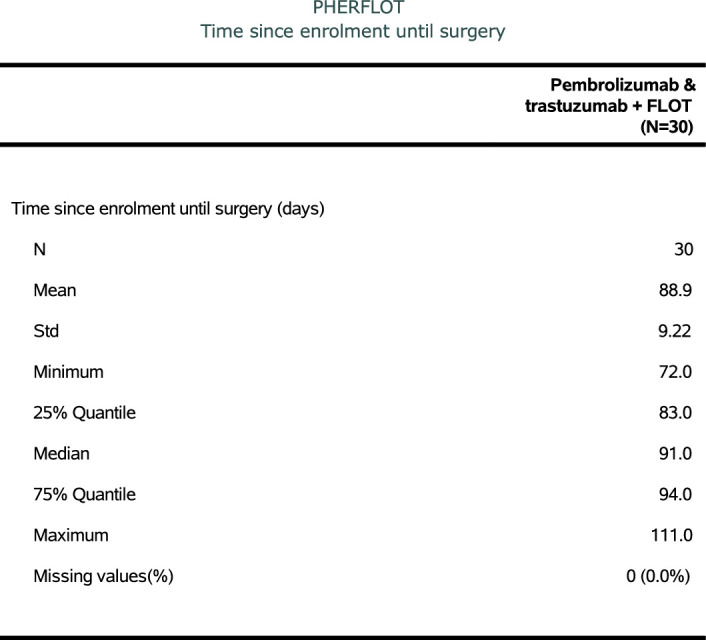
Time to surgery

Time from enrollment to surgery (in days) is reported. N, mean, minimum, median, maximum, standard deviation (Std), and the 25th and 75th percentiles are shown.

**Extended Data Table 6:**
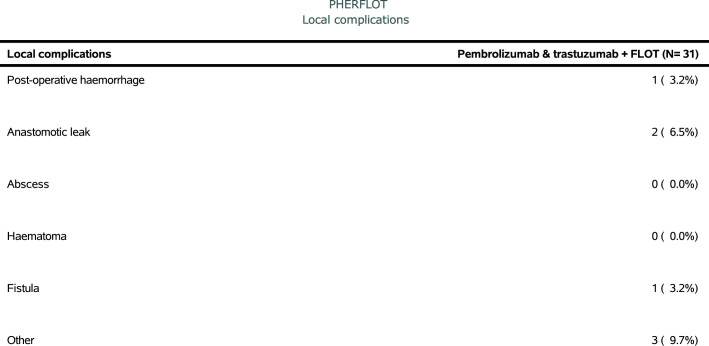
Local complications

Postoperative local complications. Number of patients and percentages are provided.

**Extended Data Table 7:**
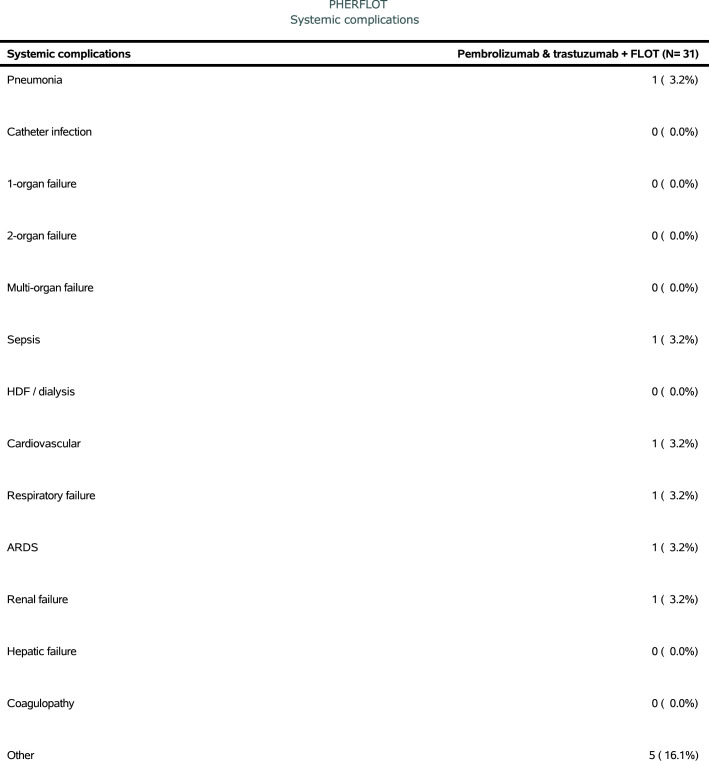
Systemic complications

Postoperative systemic complications. Number of patients and percentages are provided.

## Data Availability

Data generated or analyzed during the preparation of this publication are included in this article and its Extended Data or Supplementary Information. Anonymized individual participant data and related documents can be requested from the corresponding authors. Responses to such requests can be expected within 1 month. The trial protocol is available in the Supplementary Information.
