# Supplementary material for: Perioperative pembrolizumab, trastuzumab and FLOT in HER2-positive localized esophagogastric adenocarcinoma: a phase 2 trial
Source: Nat Med. 2025 Oct 18;31(12):4197–204. doi: 10.1038/s41591-025-03979-y (PMC12705456; doi:10.1038/s41591-025-03979-y)
Supplement: Supplementary file 1 — Supplementary Table 1 (all adverse events), Study Protocol and Statistical Analysis Plan. [file 41591_2025_3979_MOESM1_ESM.pdf]

nature medicine

Supplementary information https://doi.org/10.1038/s41591-025-03979-y

Perioperative pembrolizumab, trastuzumab and FLOT in HER2-positive localized esophagogastric adenocarcinoma: a phase 2 trial

In the format provided by the
authors and unedited

PHERFLOT
Adverse events: max grade per patient by grade and NCI CTC Term

Pembrolizumab & trastuzumab + FLOT (N=31)						
NCI CTC Term	Grade1	Grade2	Grade3	Grade4	Grade5	Total
Abdominal infection			2 (6.5%)			2 (6.5%)
Abdominal pain		2 (6.5%)				2 (6.5%)
Acidosis			1 (3.2%)			1 (3.2%)
Acute kidney injury			1 (3.2%)			2 (6.5%)
Alanine aminotransferase increased	3 (9.7%)	2 (6.5%)				5 (16.1%)
Alkaline phosphatase increased	2 (6.5%)					2 (6.5%)
Allergic reaction		1 (3.2%)	1 (3.2%)			2 (6.5%)
Alopecia	3 (9.7%)	5 (16.1%)				8 (25.8%)
Anaphylaxis			1 (3.2%)			1 (3.2%)
Anemia	2 (6.5%)	3 (9.7%)	4 (12.9%)			9 (29.0%)
Anorexia	4 (12.9%)	5 (16.1%)	1 (3.2%)			10 (32.3%)
Arthritis		1 (3.2%)				1 (3.2%)
Aspartate aminotransferase increased	6 (19.4%)					6 (19.4%)
Atrial fibrillation		1 (3.2%)				1 (3.2%)
Autoimmune disorder		1 (3.2%)				1 (3.2%)
Back pain	1 (3.2%)					1 (3.2%)
Bloating		1 (3.2%)				1 (3.2%)
Bronchial infection		1 (3.2%)				1 (3.2%)
Catheter related infection		1 (3.2%)				1 (3.2%)
Chills			1 (3.2%)			1 (3.2%)
Colitis		1 (3.2%)	1 (3.2%)			2 (6.5%)
Conjunctivitis	1 (3.2%)	2 (6.5%)				3 (9.7%)
Constipation	5 (16.1%)	5 (16.1%)				10 (32.3%)
Cough	2 (6.5%)	2 (6.5%)				4 (12.9%)
Creatinine increased	2 (6.5%)					2 (6.5%)
Dehydration		3 (9.7%)				3 (9.7%)

PHERFLOT
Adverse events: max grade per patient by grade and NCI CTC Term

	Pembrolizumab & trastuzumab + FLOT (N=31)					
**NCI CTC Term**	**Grade1**	**Grade2**	**Grade3**	**Grade4**	**Grade5**	**Total**
Diarrhea	5 (16.1%)	9 (29.0%)	12 (38.7%)			26 (83.9%)
Dizziness	1 (3.2%)					1 (3.2%)
Dry eye	1 (3.2%)					1 (3.2%)
Dry mouth		1 (3.2%)				1 (3.2%)
Dry skin	1 (3.2%)	1 (3.2%)				2 (6.5%)
Dysgeusia	3 (9.7%)	2 (6.5%)				5 (16.1%)
Dysphagia	1 (3.2%)	1 (3.2%)	3 (9.7%)			5 (16.1%)
Dyspnea	2 (6.5%)		1 (3.2%)	1 (3.2%)		4 (12.9%)
Eczema	3 (9.7%)	1 (3.2%)				4 (12.9%)
Edema limbs	2 (6.5%)	1 (3.2%)				3 (9.7%)
Encephalopathy		1 (3.2%)				1 (3.2%)
Enterocolitis infectious		1 (3.2%)				1 (3.2%)
Epistaxis	3 (9.7%)					3 (9.7%)
Erythema multiforme		1 (3.2%)				1 (3.2%)
Erythroderma		2 (6.5%)				2 (6.5%)
Esophageal anastomotic leak			1 (3.2%)	2 (6.5%)		3 (9.7%)
Esophagitis		1 (3.2%)				1 (3.2%)
Fatigue	4 (12.9%)	5 (16.1%)	1 (3.2%)			10 (32.3%)
Febrile neutropenia			1 (3.2%)			1 (3.2%)
Fever	4 (12.9%)	3 (9.7%)	2 (6.5%)			9 (29.0%)
Flu like symptoms		1 (3.2%)				1 (3.2%)
Flushing	1 (3.2%)					1 (3.2%)
Gastric stenosis			2 (6.5%)			2 (6.5%)
Gastroesophageal reflux disease	1 (3.2%)					1 (3.2%)
Gastrointestinal anastomotic leak			1 (3.2%)			1 (3.2%)
Hematuria			1 (3.2%)			1 (3.2%)

PHERFLOT
Adverse events: max grade per patient by grade and NCI CTC Term

Pembrolizumab & trastuzumab + FLOT (N=31)						
**NCI CTC Term**	**Grade1**	**Grade2**	**Grade3**	**Grade4**	**Grade5**	**Total**
Hepatic failure						1 (3.2%)
Herpes simplex reactivation	1 (3.2%)					1 (3.2%)
Hoarseness	1 (3.2%)					1 (3.2%)
Hypercalcemia		1 (3.2%)				1 (3.2%)
Hyperglycemia	1 (3.2%)		2 (6.5%)			3 (9.7%)
Hyperkalemia		1 (3.2%)				1 (3.2%)
Hyperphosphatemia	3 (9.7%)					3 (9.7%)
Hypertension		1 (3.2%)	2 (6.5%)			3 (9.7%)
Hyperthyroidism		1 (3.2%)				1 (3.2%)
Hypoalbuminemia		2 (6.5%)				2 (6.5%)
Hypoglycemia		2 (6.5%)				2 (6.5%)
Hypokalemia	2 (6.5%)	3 (9.7%)	2 (6.5%)			7 (22.6%)
Hypomagnesemia	1 (3.2%)	1 (3.2%)				2 (6.5%)
Hypophosphatemia		2 (6.5%)				2 (6.5%)
Hypotension	1 (3.2%)		1 (3.2%)			2 (6.5%)
Hypothyroidism	1 (3.2%)	3 (9.7%)				4 (12.9%)
Ileus			2 (6.5%)			2 (6.5%)
Joint range of motion decreased	1 (3.2%)					1 (3.2%)
Lipase increased	1 (3.2%)	1 (3.2%)	2 (6.5%)			4 (12.9%)
Localized edema	1 (3.2%)					1 (3.2%)
Lung infection		1 (3.2%)	1 (3.2%)			2 (6.5%)
Lymphocyte count decreased			1 (3.2%)			1 (3.2%)
Lymphocyte count increased		1 (3.2%)				1 (3.2%)
Mucositis oral	6 (19.4%)	2 (6.5%)	1 (3.2%)			9 (29.0%)
Muscle cramp	1 (3.2%)					1 (3.2%)

PHERFLOT
Adverse events: max grade per patient by grade and NCI CTC Term

Pembrolizumab & trastuzumab + FLOT (N=31)						
**NCI CTC Term**	**Grade1**	**Grade2**	**Grade3**	**Grade4**	**Grade5**	**Total**
Muscle weakness left-sided		1 (3.2%)				1 (3.2%)
Myalgia		1 (3.2%)				1 (3.2%)
Myocardial infarction		1 (3.2%)				1 (3.2%)
Myocarditis			1 (3.2%)			1 (3.2%)
Nail changes	1 (3.2%)					1 (3.2%)
Nausea	5 (16.1%)	10 (32.3%)	2 (6.5%)			17 (54.8%)
Neutrophil count decreased	1 (3.2%)	3 (9.7%)	5 (16.1%)	3 (9.7%)		12 (38.7%)
Oral hemorrhage	1 (3.2%)					1 (3.2%)
Pain	4 (12.9%)	3 (9.7%)				7 (22.6%)
Pain in extremity		2 (6.5%)				2 (6.5%)
Palpitations	1 (3.2%)					1 (3.2%)
Pancreatic enzymes decreased		1 (3.2%)				1 (3.2%)
Peripheral sensory neuropathy	14 (45.2%)	9 (29.0%)	2 (6.5%)			25 (80.6%)
Platelet count decreased	3 (9.7%)		1 (3.2%)			4 (12.9%)
Pleural effusion	2 (6.5%)	1 (3.2%)				3 (9.7%)
Pruritus	3 (9.7%)		1 (3.2%)			4 (12.9%)
Rash acneiform	2 (6.5%)	2 (6.5%)				4 (12.9%)
Respiratory failure					1 (3.2%)	2 (6.5%)
Restlessness		1 (3.2%)				1 (3.2%)
Restrictive cardiomyopathy		1 (3.2%)				1 (3.2%)
Sepsis			4 (12.9%)	1 (3.2%)	1 (3.2%)	6 (19.4%)
Sinus tachycardia	1 (3.2%)		1 (3.2%)			2 (6.5%)
Skin ulceration	1 (3.2%)					1 (3.2%)
Sleep apnea	1 (3.2%)					1 (3.2%)
Stomach pain	1 (3.2%)					1 (3.2%)

PHERFLOT
Adverse events: max grade per patient by grade and NCI CTC Term

	Pembrolizumab & trastuzumab + FLOT (N=31)					
NCI CTC Term	Grade1	Grade2	Grade3	Grade4	Grade5	Total
Syncope			1 (3.2%)			1 (3.2%)
Thromboembolic event		1 (3.2%)	1 (3.2%)			2 (6.5%)
Thyroid stimulating hormone increased	1 (3.2%)					1 (3.2%)
Upper respiratory infection		1 (3.2%)				1 (3.2%)
Urinary tract infection		2 (6.5%)	1 (3.2%)			3 (9.7%)
Ventricular arrhythmia		1 (3.2%)				1 (3.2%)
Vertigo	1 (3.2%)					1 (3.2%)
Vomiting	3 (9.7%)	3 (9.7%)	2 (6.5%)			8 (25.8%)
Weight loss	3 (9.7%)	10 (32.3%)	5 (16.1%)			18 (58.1%)
White blood cell decreased	5 (16.1%)	6 (19.4%)	4 (12.9%)			15 (48.4%)

CONFIDENTIALITY STATEMENT

This document and all of the information relating to it are the confidential property of Frankfurter Institut für Klinische Krebsforschung IKF GmbH. No part of it may be transmitted, reproduced, published, or used by other persons without the permission of Frankfurter Institut für Klinische Krebsforschung IKF GmbH.

Sponsor:

Frankfurter Institut für Klinische Krebsforschung IKF GmbH
- Represented by its Managing Director -
Prof. Dr. med. Salah-Eddin Al-Batran
[redacted]

TITLE:

Pembrolizumab and trastuzumab in combination with FLOT in the perioperative treatment of HER2-positive, localized esophagogastric adenocarcinoma - A phase II trial of the AIO study group

– PHERFLOT –

EudraCT NUMBER: 2021-006512-87 / EU CT No. 2024-513610-34-00

Sponsor's Protocol Code Number: PHERFLOT

AIO Study Number: AIO-STO-0321

Coordinating Investigator (LKP):

Dr. med. Eray Gökkurt
[redacted]

Study Management:

[redacted]

Protocol Committee:
Prof. Dr. med. Salah Al-Batran, Frankfurt/Main
Prof. Dr. med. Thorsten Götze, Frankfurt/Main
Prof. Dr. med. Ralf Hofheinz, Mannheim
Prof. Dr. med. Sylvie Lorenzen, München
Prof. Dr. med. Alexander Stein, Hamburg
Dr. med Joseph Tintelnot, Hamburg

Continuous Toxicity Monitoring Board:

[redacted]

Study Statistics:

Frankfurter Institut für Klinische Krebsforschung

[redacted]

Monitoring and Data Management:

Frankfurter Institut für Klinische Krebsforschung

[redacted]

Central Pharmacy:

[redacted]

Translational Research:

Dr. med. Eray Gökkurt
Prof. Dr. med. Alexander Stein

[redacted]

Institutional Review Board (Ethics Committee) under CTD

Ethikkommission der Ärztekammer Hamburg

[redacted]

Approval of the protocol

Pembrolizumab and trastuzumab in combination with FLOT in the perioperative treatment of HER2-positive, localized esophagogastric adenocarcinoma - A phase II trial of the AIO study group

– PHERFLOT –

Version 1.3, March 14, 2024

Signatures

Coordinating Investigator (LKP) according to AMG

Dr. med. Eray Gökkurt

[redacted]

Representative of the Sponsor

Prof. Dr. med. Salah-Eddin Al-Batran

[redacted]

Investigator’s agreement

I have read the attached protocol entitled

Pembrolizumab and trastuzumab in combination with FLOT in the perioperative treatment of HER2-positive, localized esophagogastric adenocarcinoma - A phase II trial of the AIO study group

– PHERFLOT –

Version 1.3, March 14, 2024

diligently and agree to abide by all provisions set forth therein.

I agree to comply with the International Conference on Harmonization Tripartite Guideline on Good Clinical Practice, all applicable national regulations as well as the requirements of the appropriate Institutional Review Board/Independent Ethics Committee and any other institutional requirements.

I am aware of my responsibilities as a Principal Investigator/ Deputy under the GCP national regulations and trial protocol. I agree to appropriately direct and assist the staff under my control who will be involved in this clinical trial. This is documented in a training log.

I agree to ensure that the confidential information contained in this document will not be used for any purpose other than the evaluation or conduct of the clinical investigation without the prior written consent of the study sponsor.

I consent to report every serious clinical adverse event to the sponsor within 24 hours of awareness, whether it is related to study medication or not.

Site Number: ______________

Site Name: ____

_________	___________________________	__________________________
Date	Principal Investigator, Print name	Signature
_________	___________________________	__________________________
Date	Deputy, Print name	Signature

TABLE OF CONTENTS

Table of Contents 5
Abbreviations 9
1 Trial Summary 11
2 Trial Design 14
2.1 Trial Design 14
2.2 Trial Schema 16
2.3 Schedule of activities 17
3 Objective(s), Hypothesis(es), and Endpoint(s) 21
3.1 Primary Objective, Hypothesis, and Endpoint 21
3.2 Secondary Objective(s), Hypothesis(es), and Endpoint(s) 21
3.3 Exploratory Objective 22
4 Background and Rationale 22
4.1 Esophagogastric adenocarcinoma 22
4.2 Systemic treatment of localized esophagogastric adenocarcinoma – the FLOT regimen 22
4.3 Pembrolizumab 23
4.3.1 Pembrolizumab pharmaceutical and therapeutic background 24
4.3.2 Preclinical and clinical trial data 25
4.4 PD-1 and PD-L1 inhibition in HER2 negative esophagogastric adenocarcinoma as 1st-line therapy 25
4.5 Trastuzumab 26
4.5.1 Trastuzumab, pharmaceutical and therapeutic background 27
4.5.2 Trastuzumab in esophagogastric cancer 27
4.6 Combination of checkpoint inhibition and HER2 targeting agents 27
4.7 Rationale 28
4.7.1 Rationale for the trial and selected population 28
4.7.2 Justification for dosage of FLOT chemotherapy 28
4.7.3 Justification for dose of pembrolizumab 28
4.7.4 Justification for dose of trastuzumab 29
4.7.5 Justification for treatment duration 29
4.7.6 Rationale for endpoints 29
4.7.7 Benefit risk assessment 30
5 Methodology 31
5.1 Study population 31
5.1.1 Participant inclusion criteria 31
5.1.2 Participant exclusion criteria 33
5.1.3 Lifestyle considerations 35
5.1.4 Pregnancy 35
5.2 Trial intervention(s) 36
5.2.1 Timing of dose administration 37
5.2.2 Prerequisites for application of study intervention 38
5.2.3 Dose modification and toxicity management of FLOT chemotherapy. 39
5.2.4 Dose Modification and toxicity management for immune-related AEs associated with pembrolizumab and combination therapy 40

5.2.5 Dose modifications and toxicity management for AEs associated with trastuzumab and combination therapy ... 47
5.3 Concomitant medications/vaccinations (allowed & prohibited) ... 48
5.3.1 Acceptable concomitant medications ... 48
5.3.2 Prohibited concomitant medications ... 49
5.3.3 Rescue medications & supportive care ... 50
5.4 Surgery ... 50
5.4.1 Resection of the primary tumor ... 50
5.4.2 Tumors of the gastroesophageal junction (GEJ I-III) ... 51
5.4.3 Gastric cancer ... 51
5.4.4 Splenectomy ... 52
5.4.5 Pancreatic resection ... 52
5.5 Participant Discontinuation Criteria ... 53
5.6 Participant withdrawal from study ... 53
5.7 Clinical criteria for early trial termination ... 54
5.7.1 Handling premature treatment (whole treatment) termination ... 54
5.7.2 Handling disease progression prior to surgery ... 54
5.7.3 Handling inoperability defined during surgery and R1/2 resection ... 55
6 Trial Assessments and Procedures ... 55
6.1 Trial Procedures ... 55
6.1.1 Administrative and general procedures ... 56
6.1.2 Clinical Procedures/Assessments ... 59
6.1.3 Clinical safety laboratory procedures/ assessments ... 63
6.1.4 Other Procedures ... 65
6.1.5 Visit requirements ... 65
6.2 Adverse Events (AEs), Serious Adverse Events (SAEs), and Other Reportable Safety Events ... 73
6.2.1 Time period and frequency for collecting AE, SAE, and other reportable safety event information ... 74
6.2.2 Method of Detecting AEs, SAEs, and other reportable safety events ... 75
6.2.3 Follow-up of AE, SAE, and other reportable safety event information ... 76
6.2.4 Sponsor responsibility for reporting AEs ... 76
6.2.5 Pregnancy and exposure during breastfeeding ... 76
6.2.6 Events of clinical interest (ECIs) ... 76
6.3 Treatment of Overdose ... 77
7 Continuous toxicity Monitoring board ... 77
8 Accompanying Research Project ... 78
8.1 Sampling Time Points, Materials and Analyses ... 78
8.1.1 Tissue Sample ... 78
8.1.2 Blood Samples ... 78
8.1.3 Stool and Saliva Samples ... 79
9 Statistical Analysis Plan ... 79
9.1 Justification of sample size ... 79
9.2 Statistical analysis plan ... 80
9.3 Analysis ... 81
9.3.1 Population for analysis ... 81
9.3.2 Primary endpoint ... 81

9.3.3 Secondary endpoints 82
9.3.4 Exploratory endpoints 82
10 Labeling, Packaging, Storage and Return of Clinical supplies 82
10.1 Investigational product 82
10.2 Packaging and labeling information 83
10.3 Clinical supplies disclosure 83
10.4 Storage and handling requirements 83
10.5 Returns and reconciliation 83
11 Administrative and Regulatory Details 84
11.1 Regulatory and ethical compliance 84
11.2 Registration and request for authorization of the trial 84
11.3 Ethics committee 84
11.4 Informed consent 85
11.5 Insurance 85
11.6 Confidentiality 85
11.7 Confidentiality of subject records 86
11.8 Confidentiality of investigator information 86
11.9 Compliance with financial disclosure requirements 87
11.10 Quality management system 87
11.10.1 Quality control and quality assurance 87
11.10.2 Audits and inspections 87
11.10.3 Monitoring 87
11.11 Notification of Serious Breaches 88
11.12 Data management 88
11.12.1 Data Handling/Data Capture System 89
11.12.2 Plausibility check, data cleaning and coding 89
11.12.3 Publication and registration of the study 89
12 References 90
13 Appendices 93
Appendix 1: ECOG Performance Status 93
Appendix 2: Contraceptive guidance and pregnancy testing 94
Appendix 3: Adverse Events: Definitions and procedures for recording, evaluating, follow-up, and reporting 96
Appendix 4: HER2 testing in gastric cancer 103
Appendix 5: Translational research working instructions 104

LIST OF TABLES

Table 1 Schedule of activities 17
Table 2 Adequate organ function laboratory values 32
Table 3 Trial interventions 36
Table 4 Dose adjustment in case of oxaliplatin-related neurotoxicity 39
Table 5 Dose modification and toxicity management guidelines for immune-related AEs associated with pembrolizumab monotherapy and IO Combinations 42
Table 6 Pembrolizumab Infusion reaction dose modification and treatment guidelines 45
Table 7 Laboratory tests 64
Table 8 Reporting time periods and time frames for AEs and other reportable safety events 75

Table 9 Product descriptions 83
Table 10 Appendix 1 - ECOG Performance Status [43] 93
Table 11 Appendix 2 - Highly effective contraception methods 95
Table 12 Appendix 3 - IHC scoring for HER2 103

LIST OF FIGURES

Figure 1 Study flow chart 16
Figure 2 Treatment overview 37
Figure 3 Appendix 3 - HER2 testing algorithm 103

Abbreviations

5-FU	5-fluorouracil
ADCC	antibody-dependent cellular cytotoxicity
AE	adverse event
ALT	Alanine aminotransferase /serum glutamic pyruvic transaminase
AMG	Arzneimittelgesetz
ASCO	American Society of Clinical Oncology
AST	Aspartate aminotransferase /serum glutamic oxaloacetic transaminase
CD	cluster of differentiation
CPS	combined prognostic score
CR	complete response
CT	Computer tomography
CTCAE	National Cancer Institute Common Terminology Criteria for Adverse Events
CTD	Clinical Trial Directive
CTLA-4	cytotoxic T-lymphocyte associated protein 4
CTR	Clinical Trial Regulation
DFS	disease free survival
DLT	dose limiting toxicity
DPD	dihydropyrimidine dehydrogenase
DRESS	drug rash with eosinophilia and systemic symptom
dx	day x
ECF	Epirubicin, Cisplatin and 5-FU
ECG	Electrocardiogram
ECG	Electrocardiogram
ECI	events of clinical interest
ECOG	Eastern Cooperative Oncology Group
eCRF	electronic case report form
ECX	Epirubicin, Cisplatin and Xeloda
EGFR	epidermal growth factor receptor
EMA	European Medicines Agency
EOT	End of treatment
FDA	U.S. Food and Drug Administration
FFPE	Formalin-fixed, paraffin embedded
FLOT	fluorouracil plus leucovorin, oxaliplatin and docetaxel
FOLFOX	folinic acid, fluorouracil, and oxaliplatin
FPI	first patient in
FSH	follicle stimulating hormone
FU	Follow-up
GCP	Good Clinical Practice
G-CSF	colony stimulating factor
GEJ	gastroesophageal junction
GI	gastrointestinal
HER2	human epidermal growth factor receptor 2
HIV	Human immunodeficiency virus
HRT	hormonal replacement therapy
IAR	infusion related reaction
ICF	Informed Consent Form
ICH	International Conference on Harmonization or immunohistochemistry
IO	immuno-oncology
irAEs	immune-related AEs
IRB/ERC	Institutional Review Board/ Ethical Review Committee
ISH	in situ hybridization
ITT	Intention-to-treat
IV	intravenous
LAD	lymphadenectomy
LDH	Lactate dehydrogenase
LKP	Leiter der Klinischen Prüfung (coordinating investigator)
LPFT	last patient first treatment
LPI	last patient in
LPLT	last patient last treatment
LPO	last patient out
LVEF	left ventricular ejection fraction
mAb	monoclonal antibody
MASCC	Multinational Association of Supportive Care in Cancer
MRI	Magnetic Resonance Imaging
MRI	Magnetic resonance imaging
MSI	Microsatellite instability
NCI	National Cancer Institute
NCI	National Cancer Institute
ORR	objective response rate
OS	overall survival
pCR	pathological complete response
PD	progressive disease
PD-1	programmed cell death protein 1
PD-L1/2	programmed cell death ligand 1/2
PEI	Paul-Ehrlich-Institut
PFS	Progression-free survival
po	per oral
PR	partial response
QxW	every x weeks

RECIST	Response evaluation criteria in solid tumors
SAE	serious adverse events
SAP	statistical analysis plan
SAR	serious adverse reaction
SJS	Stevens-Johnson Syndrome
SmPC	Summary of Product Characteristics
SOC	standard of care
SOP	Standard Operating Procedure
SUSAR	Suspected unexpected serious adverse reaction
T1DM	type 1 diabetes mellitus
TEN	toxic epidermal necrolysis
T-regs	regulatory T-cells
TTP	time to progression
ULN	upper limit of normal
WHO	world health organization
WOCBP	Woman of Child-Bearing Potential

1 TRIAL SUMMARY

**Abbreviated Title**	PHERFLOT – Pembrolizumab and trastuzumab in combination with FLOT in the perioperative treatment of HER2-positive, localized esophagogastric adenocarcinoma - A phase II trial of the AIO study group
**Trial Phase**	Phase II
**Clinical Indication**	HER2 positive, localized esophagogastric adenocarcinoma
**Trial Type**	single arm, open label, multicenter, phase II trial
**Type of control**	No control arm
**Route of administration**	• FLOT regimen intravenous (IV)<br>• Pembrolizumab intravenous (IV)<br>• Trastuzumab-biosimilar intravenous (IV)
**Trial Blinding**	Unblinded, open-label
**Treatment**	<u>Preoperative Study-Treatment*:</u><br>o Pembrolizumab: 200 mg flat dose IV over 30 min, d1, d22, d43<br>o Trastuzumab: loading dose 8 mg/kg IV over 90 min, d1<br>6 mg/kg IV over 30 min**, d22, d43<br>o FLOT:<br>Docetaxel 50 mg/m² IV over 1 hour<br>Oxaliplatin 85 mg/m² IV over 2 hours<br>Folinic Acid*** 200 mg/m² IV over 1 hour<br>5-FU**** 2600 mg/m² IV over 24 hours<br>Every 2 weeks (d1, d15, d29, d43*)<br><br>*Therapy can also be administered over two days, administering pembrolizumab/trastuzumab on first day and FLOT on following day at timepoints where combination is planned.<br>**if initial dose was well tolerated<br>***Folinic acid can be applied according to local standards (product & dosing).<br>****See section 5.2 for dose adjustments in patients with a reduced DPD activity<br><br><u>Surgical Resection</u><br>is recommended to be scheduled 4-6 weeks after last preoperative treatment.

	<u>Postoperative Study-Treatment:</u><br>Within 4-10 weeks after Surgery<br>o Combination of Pembrolizumab/Trastuzumab/FLOT as described above, d1-d43<br>followed by combination of<br>o Pembrolizumab (200 mg flat dose) and Trastuzumab (6 mg/kg) for up to 11 further cycles (Q3W)<br>In total patients will receive up to 17 pembrolizumab/trastuzumab administrations (including pre- and postoperative applications).
**Number of trial participants**	30
**Estimated enrollment period**	15 months (counted after at least 80% of sites activated), overall 18 months
**Estimated duration of trial**	max. 32 months from FPI to LPLT, followed by max. 24 months of survival follow up<br><br>consisting of:<br>18 months recruiting (FPI to LPI)<br>+ max. 14 months of treatment (LPFT to LPLT)<br>+ max 24 months FU for OS after LPLT
**Duration of Participation**	Each subject will participate in the trial from the time the subject signs the Informed Consent Form (ICF) through the final contact (last follow-up visit).<br><br>All eligible patients will receive pembrolizumab at a dosage of 200 mg flat dose in combination with trastuzumab (6 mg/kg after loading dose of 8 mg/kg) every 3 weeks and 5-FU 2600 mg/m^2 for 24 h, folinic acid 200 mg/m^2, oxaliplatin 85 mg/m^2 and docetaxel 50 mg/m^2 (FLOT regimen) every 2 weeks for 8 weeks, followed by surgical resection 4 weeks after last preoperative treatment at the earliest, followed by further 8 weeks of the same regimen (within 4-10 weeks), followed by pembrolizumab 200 mg and trastuzumab 6 mg/kg alone for up to 11 cycles. In total 1 year of systemic treatment (17 pembrolizumab/ trastuzumab administrations max. per patient incl. pre- and postoperative chemo-immunotherapy).<br><br>Treatment will continue until relapse/progressive disease (PD), unacceptable adverse events (AEs), intercurrent illness that prevents further administration of treatment, investigator’s decision to withdraw the subject, patient´s wish or withdrawal, pregnancy of the female subject, noncompliance with study intervention or procedure requirements, administrative reasons requiring cessation of treatment, or completion of treatment per protocol.

	Subjects who discontinue for reasons other than relapse/PD will have post-treatment follow-up for disease status until relapse/PD, initiating a non-study cancer treatment, withdrawing consent, or becoming lost to follow-up. All subjects will be followed for overall survival (OS) until death, withdrawal of consent, loss to follow-up, or the end of the study.<br><br>After the last administration of study medication, each subject will be followed for 30 days for AE monitoring. Serious adverse events (SAEs) and Events of Clinical Interest (ECIs) will be collected for 90 days after the last administration of study medication or for a minimum of 30 days after the end of treatment if the subject has initiated a new anticancer therapy, whichever is earlier.<br><br>***Pembrolizumab and/or trastuzumab -related SAEs (SAR) and ECIs regarding pembrolizumab must be collected for 90 days after the last administration of study medication independent of the start of a new anticancer therapy.***
**Estimated average length of treatment per patient**	12 months of systemic treatment + about 2-3 months of perioperative management including primary tumor surgery

2 TRIAL DESIGN

2.1 Trial Design

This is a multicenter, single arm, prospective, open-label phase II trial investigating the clinical activity of a perioperative therapy consisting of a combination of pembrolizumab, trastuzumab and FLOT, followed by pembrolizumab plus trastuzumab alone for a maximum systemic treatment duration of one year (in total up to 17 pembrolizumab/trastuzumab administrations).

Patients suffering from previously untreated localized HER2 (human epidermal growth factor receptor 2) positive (as per Appendix 4) esophagogastric adenocarcinoma (≥ T2 any N+ or any T N+) without evidence of metastatic disease will be included in the study and are scheduled to receive a perioperative therapy consisting of a combination of pembrolizumab, trastuzumab and FLOT, followed by pembrolizumab plus trastuzumab alone until tumor relapse/progression or occurrence of limiting toxicity for a maximum of 17 administrations of systemic treatment with pembrolizumab and trastuzumab (incl. pre- and postoperative chemo-immunotherapy). Similar to prior trials in locally advanced esophagogastric adenocarcinoma (KeyNote 585[35], MATTERHORN[45], DANTE (ongoing, NCT03421288), PETRARCA[29]) and in conjunction with other adjuvant immunotherapy application schedules (e.g. for melanoma, triple negative breast cancer or urothelial carcinoma) treatment with pembrolizumab and trastuzumab will be limited to one year. The rates of HER2 and PD-L1 (CPS of at least 1) positivity vary between 70 and 85%. We recently showed in the phase II INTEGA trial no correlation between survival and PD-L1 status in HER2 positive disease treated with immunotherapy and trastuzumab[44]. Thus, only HER2 status (and not PD-L1 status) will be applied as molecular selection factor in this trial.

The primary objective of this phase II study is to demonstrate the efficacy of the FLOT/trastuzumab/pembrolizumab regimen in terms of an improvement in disease free survival (DFS) according to RECIST v1.1 and an increase in the pathological complete response (pCR) rate compared to historical controls (interim read out after surgery of last patient in study with 18 months recruitment after 24 months). Secondary objectives are further efficacy and tolerability parameters, including overall response rate according to RECIST v1.1, R0 resection rate, overall survival, safety, and tolerability (including perioperative morbidity).

Imaging examinations will be performed using RECIST 1.1 for determining assessment of response. Imaging assessments will be performed after the pre-operative treatment with pembrolizumab, trastuzumab and FLOT before surgery and then every 3 months (Q3M) independent of treatment delays. RECIST 1.1 will be used by the site for treatment decisions until first radiologic evidence of relapse/ progressive disease (PD) during neoadjuvant treatment.

Subjects will continue to be treated with pembrolizumab plus trastuzumab until progressive disease or relapse during treatment, unacceptable adverse events (AEs), intercurrent illness that prevents further administration of treatment, investigator’s decision to withdraw the

subject, patient´s wish or withdrawal, pregnancy of the subject, noncompliance with study intervention or procedure requirements, administrative reasons requiring cessation of treatment, or the subject has received all study interventions as per protocol.

Subjects who discontinue study intervention for reasons other than relapse/PD will have post-treatment follow-up for disease status until relapse/PD, initiating a non-study cancer treatment, withdrawing consent, or becoming lost to follow-up. All subjects will be followed for overall survival (OS) until death, withdrawal of consent, loss to follow-up, or the end of the study – whichever comes first.

AEs will be monitored throughout the study and graded in severity according to the guidelines outlined in the National Cancer Institute (NCI) Common Terminology Criteria for Adverse Events (CTCAE) version 5.0 (Appendix 3). After the end of treatment, each subject will be followed for 30 days after last administration of study medication for AE monitoring (pembrolizumab and/or trastuzumab -related AEs for 90 days). Serious adverse events (SAEs) and events of clinical interest (ECIs) will be collected for 90 days after the last administration of study medication or 30 days after the last administration of study medication if the subject initiates new anticancer therapy, whichever is earlier. ***Pembrolizumab and/or trastuzumab -related SAEs (SARs) and ECIs regarding pembrolizumab must be collected for 90 days after the last administration of study medication independent of the start of a new anticancer therapy.***

There will be a near real time monitoring of safety parameters (e.g., SAEs reported, any potential unexpected adverse events, accumulation of certain non-serious AEs) by a continuous toxicity monitoring board for the first 6 patients enrolled to immediately identify any risks for patient safety. In addition to these ad hoc scheduled meetings depending on SAE reporting, the toxicity monitoring board will meet after the first 6 patients have finished the third treatment with pembrolizumab plus trastuzumab and have passed their presurgical assessment to re-evaluate the risk-benefit ratio of the study and provide a recommendation on the continuation of the study to the Coordinating Investigator and Sponsor. Recruitment can be halted at the discretion of the toxicity monitoring board.

There is no full interim analysis planned for this study, due to the small sample size and the relatively short recruitment period. However, single objectives like the pCR rate will be analyzed as soon as sufficient events are available for analysis (after surgery of the last patient) as detailed in the statistical analysis plan (SAP) (Section 9).

This study will be conducted in conformance with Good Clinical Practices (GCP).

Specific procedures to be performed during the trial, as well as their prescribed times and associated visit windows, are outlined in the schedule of activities (Section 2.3). Details of each procedure are provided in trial assessments and procedures (Section 6).

2.2 Trial Schema

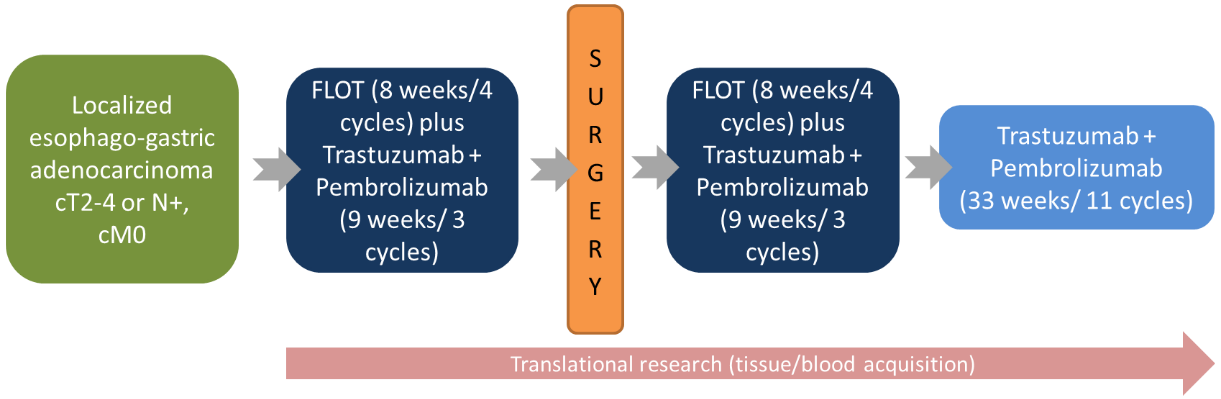

Figure 1 Study flow chart

2.3 Schedule of activities

Table 1 Schedule of activities

	Screening	Study Intervention													EOT	Follow-up	
	Study Screening	Pre-operative Treatment Pembrolizumab, Trastuzumab, FLOT					Before Surgery	After Surgery	Post-operative Treatment Pembrolizumab, Trastuzumab, FLOT					Pembrolizumab + Trastuzumab (Q3W) (up to 11 Cycles)		Safety Follow-up	Efficacy and Survival Follow Up[q]
Scheduled Timing in days (Scheduling Window in days):	≤ -4 weeks (-28 to -1)	1 (±3	15 (±3)	22 (±3)	29 (±3)	43 (±3)	4-6 weeks after last treatment	4-10 weeks after surgery	1 (±3)	15 (±3)	22 (±3)	29 (±3)	43 (±3)	64 up to further 11 cycles (Q3W)	At time of discon. (±4)	30 days post discon. (±7)	Q3M post discon. (±7)
**Administrative Procedures**																	
Informed Consent	X																
Inclusion/Exclusion Criteria	X																
HER2	X[m]																
Demographics and Medical History including history of blood transfusions	X																
Prior and Concomitant Medication Review	X	X	X	X	X	X	X	X	X	X	X	X	X	X	X	X	
**Clinical Procedures/Assessments**																	
Review Adverse Events	X	X	X	X	X	X	X	X	X	X	X	X	X	X	X	X (up to 90d)	
Full /Directed Physical Examination [b]	X[a]	X[b]					X	X	X[b]					X[b]	X		
ECOG Performance Status	X[a]	X[a]	X	X	X	X	X	X	X	X	X	X	X	X	X	X	
Vital Signs [c]	X[a]	X[a]	X	X	X	X	X	X	X	X	X	X	X	X	X	X	

	Screening	Study Intervention													EOT	Follow-up	
	Study Screening	Pre-operative Treatment Pembrolizumab, Trastuzumab, FLOT					Before Surgery	After Surgery	Post-operative Treatment Pembrolizumab, Trastuzumab, FLOT					Pembrolizumab + Trastuzumab (Q3W) (up to 11 Cycles)		Safety Follow-up	Efficacy and Survival Follow Up[q]
Scheduled Timing in days (Scheduling Window in days):	≤ -4 weeks (-28 to -1)	1 (±3	15 (±3)	22 (±3)	29 (±3)	43 (±3)	4-6 weeks after last treatment	4-10 weeks after surgery	1 (±3)	15 (±3)	22 (±3)	29 (±3)	43 (±3)	64 up to further 11 cycles (Q3W)	At time of discon. (±4)	30 days post discon. (±7)	Q3M post discon. (±7)
12-Lead ECG	X[a]	X[a]	X[d]				X		X[d]					X[d]	X		
Echocardiography	X[a]							X						X[t]	X		
Pembrolizumab Administration*		X		X		X			X		X		X	X			
Trastuzumab Administration**		X		X		X			X		X		X	X			
FLOT Administration***		X	X		X	X			X	X		X	X				
Post-study anticancer therapy status															X	X	X
Survival status		X	X	X	X	X	X	X	X	X	X	X	X	X	X	X	X
**Laboratory Procedures/Assessments: analysis performed by LOCAL laboratory**																	
Pregnancy test – urine or serum β-hCG[e]	X[e]	at monthly intervals and whenever an expected menstruation cycle is missed, or pregnancy is otherwise suspected for up to 7 months after last administration of study medication															
PT/INR and aPTT[f, l]	X[a]	X[d]					X		X[d]					X[d]	X		
Hematology and chemistry panel[g,h,l]	X[a]	X[a]	X	X	X	X			X	X	X	X	X	X	X	X	
Urinalysis[i]	X[a]	X[d]					X		X[d]						X		
TSH (fT3, fT4)[k, l]	X[a]	X[a]	X	X	X	X			X	X	X	X	X	X	X		
HIV, Hepatitis B & C	X[a]																
**Efficacy Measurements**																	
Tumor Imaging	X[n]						X[o]	X[v]	X[o]						X[p]		X[o]

	Screening	Study Intervention													EOT	Follow-up	
	Study Screening	Pre-operative Treatment Pembrolizumab, Trastuzumab, FLOT					Before Surgery	After Surgery	Post-operative Treatment Pembrolizumab, Trastuzumab, FLOT					Pembrolizumab + Trastuzumab (Q3W) (up to 11 Cycles)		Safety Follow-up	Efficacy and Survival Follow Up[q]
Scheduled Timing in days (Scheduling Window in days):	≤ -4 weeks (-28 to -1)	1 (±3	15 (±3)	22 (±3)	29 (±3)	43 (±3)	4-6 weeks after last treatment	4-10 weeks after surgery	1 (±3)	15 (±3)	22 (±3)	29 (±3)	43 (±3)	64 up to further 11 cycles (Q3W)	At time of discon. (±4)	30 days post discon. (±7)	Q3M post discon. (±7)
**Tumor Biopsies/Archival Tissue Collection/Correlative Studies**																	
Tissue Collection Baseline Biopsy / Surgery	X[r)]							X[r)]									
Blood samples	X[s)]		X				X	X	X (Q3M)								
Stool Samples & Saliva	X						X						X[u)]	X[u)]			

* Pembrolizumab 200 mg day 1 Q3W

** Trastuzumab Q3W, 8 mg/kg at d1 (pre- & post-OP), 6 mg/kg d22-43, and when given without chemotherapy Q3W.

*** FLOT regimen (4 bi-weekly cycles pre- & post-OP, i.e. d1, 15, 29, 43): Oxaliplatin 85 mg/m^2; Folinic Acid 200 mg/m^2; 5-FU 2.600 mg/m^2; Docetaxel 50 mg/m^2. Investigators can also use their local protocols for FLOT.

a. To be performed within 14 days prior to first study medication administration; can be skipped at d1 if screening assessment is not older than 14 days

b. Full Physical Examination to be performed at baseline, before and after surgery and at EOT; Directed Physical Examination otherwise. Full physical examination includes heart, chest, abdomen, skin, lymph nodes, neurological exam, inspection of accessible mucosa and weight, height (only baseline); Directed Physical Examination: as clinically indicated.

c. Vital signs should include temperature, pulse, respiratory rate, blood pressure, oxygen saturation

d. As clinically indicated.

e. To be performed in WOCBP only. If applicable, this test should be repeated a maximum of 24 h before the study medication administration. During treatment and for up to 7 months after the last dose, pregnancy testing will be performed at monthly intervals and whenever an expected menstrual cycle is missed or when pregnancy is otherwise suspected, in accordance with the CTFG guidance on contraception.

f. Quick's time [PT/INR], aPTT.

g. Complete blood count CBC), hemoglobin, hematocrit, White Blood Cell (WBC) count with differential & absolute neutrophil; absolute lymphocyte; red blood cells (RBCs) and platelet count
h. albumin; alkaline phosphatase; alanine aminotransferase (ALT); aspartate aminotransferase (AST); lactate dehydrogenase (LDH); uric acid; Calcium; Glucose; Phosphorus; Potassium; Sodium; Magnesium; Total Bilirubin; Direct Bilirubin (If total bilirubin is elevated above the upper limit of normal); Total protein; Blood Urea Nitrogen; C-reactive protein (CRP); Gamma-GT; Creatinine, creatinine clearance, lipase.
i. Urinalysis by qualitative examination (stick) for: blood, glucose, proteins, nitrites, ketones, leucocytes, gravity, pH.
j. To be repeated every second cycle from the third pembrolizumab plus trastuzumab cycle onwards or when clinically indicated.
k. fT3, fT4 to be analyzed if TSH is outside of normal range.
l. These assessments may be performed up to 1 day prior to the visit (entire study) in order to have the results available on the visit day (pre-dose).
m. According to local laboratory standards. Testing should be timed according to clinical workflow. HER2 positivity is an inclusion criterion. For HER2 testing in gastric cancer see Appendix 4.
n. Screening tumor imaging has to be performed within 28 days (± 3 days) prior first study drug administration (available imaging before study inclusion within these timelines should be used)
o. Imaging during treatment phase will be performed after the end of the preoperative treatment/before surgery and then every 3 months (± 7 days) until the start of a further anticancer therapy or relapse/disease progression, calculated from the date of the last imaging; an earlier imaging can be performed if clinically indicated. After 2 years since EOT, imaging will be done every 6 months (± 7 days).
p. Subjects who discontinue study therapy without confirmed relapse/PD per RECIST, tumor imaging should be performed at the time of treatment discontinuation (± 4 weeks). If previous tumor imaging was obtained within 4 weeks prior to the date of discontinuation, then additional tumor imaging at treatment discontinuation is not required.
q. Every 3 months for efficacy (see also o) for imaging) and survival up to 24 months after last patient last treatment. After first progression/relapse only survival status to be documented (could also be done by a phone call).
r. Baseline sample: Archival tumor tissue sample or newly obtained (if obtained within clinical routine) (baseline sample). Surgery sample: at least 1 representative sample, primary tumor preferred to lymph nodes. Formalin-fixed, paraffin embedded (FFPE) tissue blocks are preferable to slides.
s. Blood samples (2x 10mL Streck® tubes for accompanying research project to be taken at baseline (day 1 or up to 7 days before first study medication administration), before second FLOT (d15), pre- and postoperatively and every 3 months afterwards until relapse/progression.
t. Echocardiography every 3 months during trastuzumab treatment
u. Stool samples and saliva for accompanying research project to be taken at baseline, preoperatively, after completion of adjuvant chemotherapy (1st cycle of trastuzumab and pembrolizumab without chemotherapy) and 3 months after completion of adjuvant chemotherapy.
v. After surgery tumor assessment should be performed by sonography or X-rays (or other standard methods) according to the common S3 guidelines to determine the absence of the tumor/metastases.

3 OBJECTIVE(S), HYPOTHESIS(ES), AND ENDPOINT(S)

3.1 Primary Objective, Hypothesis, and Endpoint

Objective: Assessment of co-primary objectives of disease-free survival and the pathological complete response rate

Hypothesis: We hypothesize that the DFS rate after 2 years (DFSR@2) should be 70% or more and pCR rate is 30% or greater.

CO-Primary Endpoints:

DFSR@2 is defined as the proportion of patients being tumor/disease free and alive 2 years after enrolment.

The pCR rate is defined as the absence of residual tumor based on evaluation of the resected esophagogastric specimen in the primary tumor by local pathology.

3.2 Secondary Objective(s), Hypothesis(es), and Endpoint(s)

a) **Objective**: Assessment of overall response rate (ORR) according to RECIST v1.1,

   Secondary Endpoint: ORR – percentage of patients with CR or partial response (PR) according to RECIST 1.1.

b) **Objective**: Assessment of R0 resection rate

   Secondary Endpoint: R0 resection - microscopically margin negative resection with no gross or microscopic tumor remains in the areas of the primary tumor and/or sampled regional lymph nodes.

c) **Objective**: Assessment of Overall survival (OS)

   Secondary Endpoint: OS – time from enrolment to the date of death of any cause

d) **Objective**: Assessment of Feasibility

   Hypothesis: We hypothesize that a treatment regimen of pembrolizumab and trastuzumab in combination with FLOT 8 weeks pre- as well as post-surgery, followed by pembrolizumab and trastuzumab treatment for up to one year (from the start of the study treatment) is feasible for most patients, and that no more than 1/3 of patients have discontinued the treatment prior to the end of postoperative chemotherapy for a reason other than progressive disease (i.e., feasibility rate ≥ 0.66).

Secondary Endpoint: Feasibility rate - severe toxicity/withdrawal rate before the last postoperative administration of pembrolizumab/trastuzumab/FLOT has been completed.

e) Objective: Assessment of Safety and toxicity

Hypothesis: We hypothesize that the number of patients with at least 1 AE of CTCAE grade ≥ 3 will be slightly higher than with FLOT and trastuzumab (compared to AIO HERFLOT trial 28).

Secondary Endpoint: (Serious) adverse events - recorded and graded according to NCI-CTCAE V5.0. Occurrence of (serious) adverse events at any time during the study. Description by nature (System Organ Class and Preferred Term), severity and causal relationship to drug administration.

3.3 Exploratory Objective

- **Objective:** Assessment whether clinical efficacy correlates with molecularly-defined subgroups (PD-L1 expression, MSI subtypes, and others).

4 BACKGROUND AND RATIONALE

4.1 Esophagogastric adenocarcinoma

With more than 1.5 million cases in 2018 esophageal and gastric cancer belong to the most common malignancies worldwide. Both diseases are associated with a high disease-related mortality, resulting in approximately 1.3 million deaths per year.[2] In esophageal carcinoma a strong increase in the incidence of adenocarcinomas has been observed during the last decades, representing the most common subtype of esophageal carcinoma in Northern America, Northern Europe and Oceania. In addition, an increase of adenocarcinomas of the gastroesophageal junction has been observed during the last decades, while the incidence of non-cardiac gastric cancer is declining in western countries.

4.2 Systemic treatment of localized esophagogastric adenocarcinoma – the FLOT regimen

Emerging data from recent phase III trials indicate that immune checkpoint inhibitors such as Pembrolizumab prolong OS and PFS when added to the doublet chemotherapy in advanced esophagogastric adenocarcinoma. However, the extent of improvement regarding PFS is smaller than expected and if administered as monotherapy, there is an increased early mortality with the checkpoint inhibitors as compared with chemotherapy (crossing survival

curves).[1,3,4] This is most likely explained by the fact that patients need time to establish antitumoral immunity, while some patients with aggressive disease experience early disease progression and death.
FLOT, a docetaxel-based triple combination consisting of 5-FU, leucovorin, oxaliplatin and docetaxel, is one of the most intensively evaluated regimens for gastric and gastroesophageal junction (GEJ) cancer. It has been evaluated in the metastatic setting, in the limited metastatic setting[5], in elderly patients and in operable patients.[6-9] The AIO FLOT4 phase II/III study has evaluated FLOT versus Epirubicin, Cisplatin and 5-FU (ECF)/Epirubicin, Cisplatin and Xeloda (ECX) (n = 716) in patients with resectable gastric or gastro-oesophageal junction adenocarcinoma . The accrual has been completed in 2015.

The phase II part of the randomized phase II/III FLOT4 trial regarding histopathological regression, published in Lancet Oncology, associated FLOT with significantly higher rates of pCR as compared to ECF (20 [16%] of 128 vs. 8 [6%] of 137; p=0.02). Also, the rate of complete or subtotal regression (TRG1a/b) was significantly higher with FLOT (47 [37%] of 128 vs. 31 [23%] of 137, p=0.02).[7]

The results of the phase III part of the FLOT4 trial, published in Lancet, showed an increased OS in the FLOT group compared to the ECF/EXC group (hazard ratio [HR] 0.77; 95% confidence interval [CI; 0.63 to 0.94]; median OS, 50 months [38.33 to not reached] vs 35 months [27.35 to 46.26]). FLOT also improved PFS (median PFS, 30 months vs 18 months; HR 0.75 [0.62 - 0.91]; p = 0.004). Perioperative complications were 50% with ECF/ECX and 51% with FLOT. 30- and 90-day mortality was 3% and 8% with ECF/ECX and 2% and 5% with FLOT. The relative effect from FLOT was observed in all subgroups, including elderly and signet cell tumors, and was numerically pronounced in Siewert type 1 esophageal tumors (HR 0.60), Barrett tumors (HR 0.62), small tumors T1/2 (HR 0.66) or nodal negative tumors (HR 0.64). In multivariate analyses, parameters associated with favorable survival were FLOT therapy (HR 0.75, p = 0.006); stomach as the primary (HR 0.74; p = 0.005), and nodal negativity (HR 0.72, p = 0.022). ECOG PS of 0 showed a trend (HR 0.82; p = 0.078). Age and Lauren's type of histology had no impact on survival. Post-hoc analyses of relapse-free survival (PFS excluding patients without R0-resection) still favored FLOT (HR 0.8; p = 0.049). 87% of relapses were systemic or both systemic and locoregional. The most frequent sites of relapse were peritoneal (31%) followed by lymphatic (26%), and liver (19%).[10]

FLOT is regarded a standard chemotherapy regimen for gastric cancer in Germany in the perioperative setting and thus the best backbone protocol for the current trial.

4.3 Pembrolizumab

Pembrolizumab (Keytruda®) is a potent humanized immunoglobulin G4 (IgG4) monoclonal antibody (mAb) with high specificity of binding to the programmed cell death protein 1 (PD-1) receptor, thus inhibiting its interaction with programmed cell death ligand 1 (PD-L1) and 2 (PD-

L2). Based on preclinical *in vitro* data, pembrolizumab has high affinity and potent receptor blocking activity for PD-1. Pembrolizumab has an acceptable preclinical safety profile and is in clinical development as an intravenous (IV) immunotherapy for advanced malignancies. Keytruda® is indicated for the treatment of patients across a number of indications because of its mechanism of action to bind the PD-1 receptor on the T cell. For more details on specific indications refer to the Investigator brochure.

4.3.1 Pembrolizumab pharmaceutical and therapeutic background

The importance of intact immune surveillance function in controlling outgrowth of neoplastic transformations has been known for decades.[11] Accumulating evidence shows a correlation between tumor-infiltrating lymphocytes in cancer tissue and favorable prognosis in various malignancies. In particular, the presence of CD8+ T-cells and the ratio of $CD8^+$ effector T-cells/ $FoxP3^+$ regulatory T-cells (T-regs) correlates with improved prognosis and long-term survival in solid malignancies, such as ovarian, colorectal, and pancreatic cancer; hepatocellular carcinoma; malignant melanoma; and renal cell carcinoma. Tumor-infiltrating lymphocytes can be expanded ex vivo and reinfused, inducing durable objective tumor responses in cancers such as melanoma.[12,13]

The PD-1 receptor-ligand interaction is a major pathway hijacked by tumors to suppress immune control. The normal function of PD-1, expressed on the cell surface of activated T-cells under healthy conditions, is to down-modulate unwanted or excessive immune responses, including autoimmune reactions. PD-1 (encoded by the gene *PDCD1*) is an immunoglobulin superfamily member related to cluster of differentiation 28 (CD28) and cytotoxic T-lymphocyte-associated protein 4 (CTLA-4) that has been shown to negatively regulate antigen receptor signaling upon engagement of its ligands (PD-L1 and/or PD-L2).[14,15]

PD-1 and its family members are type I transmembrane glycoproteins containing an Ig-variable–type (IgV-type) domain responsible for ligand binding and a cytoplasmic tail responsible for the binding of signaling molecules. The cytoplasmic tail of PD-1 contains 2 tyrosine-based signaling motifs, an immunoreceptor tyrosine-based inhibition motif, and an immunoreceptor tyrosine-based switch motif. Following T-cell stimulation, PD-1 recruits the tyrosine phosphatases, SHP-1 and SHP-2, to the immunoreceptor tyrosine-based switch motif within its cytoplasmic tail, leading to the dephosphorylation of effector molecules such as CD3 zeta (CD3ζ), protein kinase C-theta (PKCθ), and zeta-chain-associated protein kinase (ZAP70), which are involved in the CD3 T-cell signaling cascade.[15-18] The mechanism by which PD-1 down-modulates T-cell responses is similar to, but distinct from, that of CTLA-4, because both molecules regulate an overlapping set of signaling proteins.[19] As a consequence, the PD-1/PD-L1 pathway is an attractive target for therapeutic intervention in esophagogastric adenocarcinoma.

4.3.2 Preclinical and clinical trial data

Refer to the Investigator's Brochure for preclinical and clinical data.

4.4 PD-1 and PD-L1 inhibition in HER2 negative esophagogastric adenocarcinoma as 1st-line therapy

During the last years several 1st-line phase III trials have been completed exploring safety and efficacy of PD-1 or PD-L1 inhibition in esophagogastric adenocarcinoma.

The use of pembrolizumab alone or in combination with chemotherapy was examined among patients with PD-L1 CPS (combined prognostic score) ≥ 1 in the KEYNOTE 062 trial, in this study pembrolizumab showed non-inferiority in comparison to chemotherapy alone in terms of overall survival. However, response rate (RR) and progression-free survival were nominally inferior to chemotherapy alone.[20] Among patients with a PD-L1 CPS ≥ 10 an improvement in overall survival in comparison to chemotherapy was observed (exploratory analysis). The combination of chemotherapy and pembrolizumab showed a trend towards improved efficacy albeit no significantly superior OS or PFS.[20]

The CheckMate 649 trial investigated the 1st-line nivolumab plus chemotherapy versus chemotherapy alone. The results showed that the combination of nivolumab and FOLFOX chemotherapy were superior regarding OS in comparison to chemotherapy alone both in patients with a PD-L1 CPS expression ≥ 5 (primary endpoint), as well as in the total study population irrespective of the PD-L1 expression. In addition, an increased response rate was observed both in PD-L1 positive patients (CPS ≥5) as well as in the all-comers population and also in PD-L1 negative patients (CPS <1).[3]

The combination of pembrolizumab and cisplatin-/fluoropyrimidine-based chemotherapy was explored in the KEYNOTE 590 trial, which included squamous cell esophageal cancer as well as adenocarcinomas of the esophagus and gastro-esophageal junction (Siewert Type 1). In this trial superior OS of the combination of pembrolizumab and chemotherapy was observed among the population with a PD-L1 CPS expression ≥ 10 as well as in the total study population, which included squamous cell carcinoma and adenocarcinoma patients. Additionally, no significant difference was observed regarding overall survival in patients with an adenocarcinoma and a PD-L1 CPS of higher or lower than 10.[4]

We recently showed in the phase II INTEGA trial no correlation between survival and PD-L1 status in HER2 positive disease treated with immunotherapy and trastuzumab.[44]

Efficacy in intent to treat population (ITT) and subgroups in the INTEGA trial.

	All (n=88) ITT		HER2+ central (n=76)		CPS=0 (n=23)		CPS≥1 (n=59)		CPS≥5 (n=46)	
	Trast/ Nivo/ Ipi	Trast/ Nivo/ FOLFOX	Trast/ Nivo/ Ipi	Trast/ Nivo/ FOLFOX	Trast/ Nivo/ Ipi	Trast/ Nivo/ FOLFOX	Trast/ Nivo/ Ipi	Trast/ Nivo/ FOLFOX	Trast/ Nivo/ Ipi	Trast/ Nivo/ FOLFOX
	N=44	N=44	N=40	N=36	N=11	N=12	N=31	N=28	N=24	N=22
**ORR**	32%	56%	35%	63%	27%	50%	36%	63%	33%	67%
**mPFS**	3.2 mo	10.7 mo	3.4 mo	10.7 mo	3.2 mo	11.4 mo	2.2 mo	10.7 mo	2.2 mo	11 mo
**PFSR @12**	15%	37%	17%	36%	20%	50%	14%	33%	7%	38%
**mDOR**	5.8 mo	9.2 mo	na	na	na	na	na	na	na	na
**mOS**	16.4 mo	21.8 mo	16.4 mo	22.4 mo	25.2 mo	30.8 mo	16.4 mo	21.6 mo	12.5 mo	21.6 mo
**OSR@ 12**	57%	70%	58%	74%	78.8%	75%	54%	71%	53%	72%

Abbreviations: Trast Trastuzumab, Nivo Nivolumab, Ipi Ipilimumab, FOLFOX folinic acid, 5-FU, oxaliplatin, ORR overall response rate, mPFS median progression free survival, PFSR@12 progression free survival rate at 12 months, mDOR median duration of response, mOS median overall survival, OSR@12 overall survival rate at 12 months, mo months, na not applicable.

For avelumab used as maintenance therapy after a platinum-based chemotherapy a comparable efficacy was observed in comparison to a prolonged chemotherapy irrespective of PD-L1 expression.[45]

In addition, a recent meta-analysis showed no clear correlation of PD-L1 expression measured by CPS and response to combined chemoimmunotherapy; a survival benefit of combined chemoimmunotherapy was observed in the all-comers population.[46]

Taken together, across those studies a synergistic benefit of chemotherapy and a PD-1 inhibitor was not limited to PD-L1 positive tumors. Furthermore, particularly in HER2 positive disease no signs of any interaction between PD-L1 status and efficacy was noted. Thus, only HER2 status (and not PD-L1 status) will be applied as molecular selection factor in this trial.

In summary, early results provide evidence of a superiority of combining chemotherapy and a PD-1 inhibitor, also irrespectively of PD-L1 status.

4.5 Trastuzumab

Trastuzumab (ONTRUZANT®) is a humanized IgG1 monoclonal antibody addressing the juxtamembrane portion of the extracellular domain of the HER2 receptor, which prevent the activation of its intracellular tyrosine kinase. Targeting of HER2 by trastuzumab induces an immune-mediated response causing the internalization and recycling of HER2. Trastuzumab is administrated as intravenous therapy and is indicated for the treatment of HER2-positive breast and gastric cancer. For more details on specific indications refer to the SmPC.

4.5.1 Trastuzumab, pharmaceutical and therapeutic background

HER2 belongs to the epidermal growth factor receptor (EGFR) family. This proto-oncogenic protein is a membrane-bound tyrosine kinase receptor promoting cell proliferation and cancer development upon activation.[21] Overexpression of HER2 leads to increased homo- and heterodimerization (e.g. HER2:HER3), which initiates a strong pro-tumorigenic signaling cascade.[22] Breast and gastric cancer cases demonstrate a substantial HER2 overexpression, predominantly driven by *HER2* amplification at the DNA level.[23,24]

Trastuzumab, the first anti-HER2 antibody developed in 1990, inhibits the dimerization of the receptor, leads to its internalization and degradation, abolishes the intracellular PI3K-AKT signaling pathway and induces antibody-dependent cellular cytotoxicity (ADCC) of the targeted cells.[25]

Trastuzumab in combination with chemotherapy improved the OS in women with HER2-positive metastatic breast cancer and, therefore, is used as standard of care (SOC) treatment for HER2-positive breast cancer for more than a decade.[26] In 2011, trastuzumab was also FDA-approved for the treatment of metastatic, HER2-positive gastric or gastroesophageal cancer.

4.5.2 Trastuzumab in esophagogastric cancer

The ToGa study, compromising trastuzumab in combination with chemotherapy versus chemotherapy alone for the treatment of HER2-positive advanced gastric or gastroesophageal junction cancer, revealed an improved OS of the combination therapy.[27] This leads to the approval chemotherapy combined with trastuzumab in the treatment of advanced stomach cancer by the FDA and EMA.

The combination of trastuzumab with FLOT as perioperative treatment for patients with HER2-positive locally advanced esophagogastric adenocarcinoma, investigated in the HER-FLOT trial, was found to be safe and showed a pCR > 20%.[28] Furthermore, the addition of trastuzumab and pertuzumab, a further anti-HER2 mAB, to perioperative FLOT treatment of HER2-positive resectable esophagogastric adenocarcinoma significantly improved the pCR and nodal negatively rates as shown in the PETRARCA trial.[29]

4.6 Combination of checkpoint inhibition and HER2 targeting agents

The HER2 receptor antibody trastuzumab induces both, antibody dependent cytotoxicity and lymphoid infiltration in the tumor tissue.[30] Recent preclinical data demonstrated the synergistic effect of combining HER2 blockade with immune checkpoint inhibition. Muller and colleagues nicely showed the high efficacy of HER2 blockade by the antibody drug conjugate trastuzumab emtansine in combination with PD-1 and CTLA4 blockade in an orthotopic breast cancer model.[31] The combination of CAPOX, trastuzumab and pembrolizumab showed an overall

response rate of 91% in HER2 positive metastatic disease.[32] Recently published data of the PANTHERA trial confirmed the high efficacy of the combination of trastuzumab, pembrolizumab and chemotherapy in another single arm phase II trial.[33] Furthermore, initial results of the phase III KEYNOTE 811 trial showed significantly increased response with the addition of pembrolizumab to chemotherapy and trastuzumab from 52% to 74% (p=0.00005).[34]

4.7 Rationale

4.7.1 Rationale for the trial and selected population

The addition of PD-1 inhibitors or trastuzumab to 1st-line chemotherapy increases response rates (KEYNOTE 059, CheckMate 649, ToGa).[3,4,27] The combination of CAPOX, trastuzumab and pembrolizumab showed an overall response rate of 91%.[32] Furthermore, the combination of perioperative FLOT with trastuzumab with or without pertuzumab significantly increased pCR rate from about 15% with FLOT to 22% or even 35% with double HER2 inhibition (AIO HER-FLOT and PETRARCA).[28,29] The KEYNOTE 585 trial (NCT03221426) recently completed recruitment for FLOT ± pembrolizumab in the perioperative setting.[35] Thus, the combination of FLOT and trastuzumab and pembrolizumab is the next step in the perioperative setting and should be evaluated.

4.7.2 Justification for dosage of FLOT chemotherapy

The FLOT chemotherapy consists of Docetaxel 50 mg/m², Oxaliplatin 85 mg/m², Calcium Folinate 200 mg/m² and 5-FU 2600 mg/m² every 2 weeks. This regimen is one of the standard regimens for perioperative use in adenocarcinomas of the stomach and esophagogastric junction in Germany and worldwide and serves as chemotherapeutical backbone in several phase II-III trials in esophagogastric cancer. Several trials combined FLOT in the dosage as described above with additional monoclonal antibodies like pembrolizumab, trastuzumab and/or pertuzumab with constant and acceptable safety profiles.

4.7.3 Justification for dose of pembrolizumab

The planned dose of pembrolizumab for this study is 200 mg every 3 weeks (Q3W). Based on the totality of data generated in the Keytruda development program, 200 mg Q3W is the appropriate dose of pembrolizumab for adults across all indications and regardless of tumor type. As outlined below, this dose is justified by:

- Clinical data from 8 randomized studies in melanoma and NSCLC indications demonstrating flat dose- and exposure-efficacy relationships from 2 mg/kg Q3W to 10 mg/kg Q2W, representing an approximate 5- to 7.5-fold exposure range (refer to IB, Section 5.2.2)

- Population PK analysis showing that both fixed dosing and weight-based dosing provides similar control of PK variability with considerable overlap in the distributions of exposures, supporting suitability of 200 mg Q3W
- Clinical data showing meaningful improvement in benefit-risk including overall survival at 200 mg Q3W across multiple indications, and
- Pharmacology data showing full target saturation in both systemic circulation (inferred from pharmacokinetic [PK] data) and tumor (inferred from physiologically-based PK [PBPK] analysis) at 200 mg Q3W.

4.7.4 Justification for dose of trastuzumab

The dosing for trastuzumab is based on the current SmPC. For the treatment of metastatic gastric cancer an initial dose with 8 mg/kg is recommended. The recommended maintenance dose for 3-weekly intervals is 6 mg/kg.

4.7.5 Justification for treatment duration

Similar to prior trials in locally advanced esophagogastric adenocarcinoma (KeyNote 585[35], MATTERHORN[47], DANTE (ongoing, NCT03421288), PETRARCA[29]) and in conjunction with other adjuvant immunotherapy application schedules (e.g. for melanoma, triple negative breast cancer or urothelial carcinoma) treatment with pembrolizumab and trastuzumab will be limited to one year.

4.7.6 Rationale for endpoints

4.7.6.1 Efficacy endpoints

The co-primary objectives of this phase II study is to demonstrate the efficacy of the FLOT/trastuzumab/pembrolizumab regimen in terms of an improvement in disease free survival according to RECIST v1.1 and an increase in the pathological complete response rate compared to historical controls (interim read out after surgery of last patient in study with 18 months recruitment after 24 months).

Secondary objectives are further efficacy and tolerability parameters, including overall response rate according to RECIST v1.1, DFS according to RECIST v1.1, R0 resection rate, overall survival, safety and tolerability (including perioperative morbidity).

4.7.6.2 Planned exploratory biomarker research

Predictive markers to tailor treatment are urgently warranted either at baseline or early during treatment.

Firstly, we will evaluate strategies to predict the outcome of checkpoint inhibition by liquid biopsy immunoprofiling at baseline and shortly after initiation of the treatment and correlate this with PD-L1 expression as potential response predictive biomarker. Therefore, tumor-infiltrating lymphocytes (TiL) repertoire will be determined by next-generation sequencing (NGS) of T-cell receptor beta (TCRβ) and immunoglobulin heavy locus (IGH). Furthermore, liquid biopsy NGS-based immunoprofiling (TCRβ & IGH) will be performed prior to treatment initiation and before the second pembrolizumab dose to determine response predictive immune signature (diversification pattern as read-out for ongoing immune activation, TiL clone expansion in peripheral blood). In addition, baseline FFPE will be centrally tested for PD-L1, MSI and EBV to account for further baseline markers with potential or likely predictive value for checkpoint-inhibition, although the coincidence of at least MSI and EBV with HER2 amplification is rare.[36,37]

Resistance to HER2 targeting in HER2 positive tumors might be present upfront or will eventually develop during treatment. Several mechanisms of treatment induction have already been shown, particularly loss of HER2 amplification.[38] Therefore, baseline FFPE and ctDNA will be assessed for HER2 (IHC and ISH in FFPE) and HER signaling alterations (amplifications and/or mutations in e.g. EGFR, HER2, HER3, PIK3CA).

The gut microbiota consists of trillions of bacteria and was recently introduced as a key factor influencing response to checkpoint inhibitor treatment in melanoma or lung cancer and chemotherapy treatment in colorectal cancer.[39-41] These microbes can either directly control an anti-tumor immune response within the intestine or interact with immune and tumor-cells inside the tumors. We will analyze the oral and intestinal microbiome by 16S rRNA sequencing before treatment initiation, at time of surgery and after chemotherapy completion to explore the stability of the microbiota during treatment, compare the different microbiota to the intra-tumoral microbiota at time of surgery (from FFPE tissue) and correlate bacterial species or diversity patterns with response to therapy to reveal potential biomarkers.

4.7.7 Benefit risk assessment

The overall outcome of esophagogastric cancer, although relevantly improving during the last decades, remains poor with more than 50% of patients recurring and consecutively dying despite aggressive treatments including perioperative chemotherapy and resection.[10] Thus, further improvements are urgently required. Different combination regimens of chemotherapy (fluoropyrimidins, oxaliplatin +/- docetaxel) with pembrolizumab and/or trastuzumab has shown clinically relevant activity with a manageable safety pattern in recent phase II and III trials. Based on these data, particularly the KEYNOTE 585 trial with FLOT and pembrolizumab [Bang 2019] and HER-FLOT and PETRARCA with FLOT and trastuzumab +/- pertuzumab in the perioperative setting with high efficacy in perioperative treatment and good tolerability of the respective regimen,[28,29] the PHERFLOT regimen seems safe and tolerable and yields a high

potential to improve the limited prognosis of HER2 positive esophagogastric cancer patients. In addition, the close meshed monitoring of the CTMB (particular the run-in safety analysis) will detect any potential unexpected adverse events.

5 METHODOLOGY

5.1 Study population

Investigators will recruit patients directly during regular clinical consultation visits at the respective trial site. All study related investigations and enrolment of patients will only be performed after a written consent was collected using the ethics committee approved patient information and consent forms (see also chapter 11.4).

Patients fulfilling the inclusion-/exclusion criteria will be captured online in the electronic case report form (eCRF) as screening patients to obtain a patient number which is used for identification during the screening process and, after enrolment, for pseudonymized identification throughout the study.

5.1.1 Participant inclusion criteria

Participants are eligible to be included in the study only if all of the following criteria apply:

1. The participant provides written informed consent for the trial.
2. Male/female* participants who are at least 18 years of age on the day of signing informed consent.

   *There are no data that indicate special gender distribution. Therefore, patients will be enrolled in the study gender-independently.
3. In the investigator's judgement, participant is willing and able to comply with the study protocol including the planned surgical treatment
4. Histologically confirmed adenocarcinoma of the GEJ (Type I-III according to Siewert´s classification) or the stomach (cT2, cT3, cT4, any N category, M0, or any T, N+, M0) that:
   - is not infiltrating any adjacent organs or structures by CT or MRI evaluation
   - does not involve peritoneal carcinomatosis
   - is considered medically and technically resectable

   Note: the absence of distant metastases must be confirmed by CT or MRI of the thorax and abdomen, and, if there is clinical suspicion of osseous lesions, a bone scan. If peritoneal carcinomatosis is suspected clinically, its absence must be confirmed by laparoscopy. Diagnostic laparoscopy is mandatory in patients with T3 or T4 tumors of the diffuse type histology in the stomach.

5. Participants must have HER2-positive disease defined as either IHC 3+ or IHC 2+, the latter in combination with ISH+, as assessed locally by a certified test on primary tumor (see Appendix 4)
6. Participants must be candidates for potential curative resection as determined by the treating surgeon
7. No prior systemic-anti cancer therapy (e.g. cytotoxic or targeted agents or radiotherapy)
8. No prior partial or complete esophagogastric tumor resection
9. ECOG (Eastern Cooperative Oncology Group) performance status score of 0 or 1
10. Male participants: A male participant must agree to use a contraception as detailed in Appendix 2 of this protocol during the treatment period and for at least 6 months after the last dose of study intervention and refrain from donating sperm during this period.

    Female participants: A female participant is eligible to participate if she is not pregnant (see Appendix 2), not breastfeeding, and at least one of the following conditions applies:

    - Not a woman of childbearing potential (WOCBP) as defined in Appendix 2

    OR

    - A WOCBP who agrees to follow the contraceptive guidance as given in Appendix 2 during the treatment period and for at least 7 months after the last dose of study intervention.
11. Participants have adequate organ function as defined in the following table (Table 2). Specimens must be collected within 14 days prior to enrolment (also to be repeated if older than 14 days at day of first treatment).

Table 2 Adequate organ function laboratory values

System	Laboratory Value
Hematological	
Absolute neutrophil count (ANC)	≥ 1500/µL
leucocytes	≥ 3000/µL
Thrombocytes	≥ 100000/µL
Hemoglobin	≥ 9.0 g/dL or ≥ 5.6 mmol/L[a]
Renal: Measured or calculated[b] creatinine clearance (CrCl)	≥ 50 mL/min
Hepatic	
Total bilirubin	≤ 1.5 ×ULN OR direct bilirubin ≤ ULN for participants with total bilirubin levels > 1.5 × ULN

System	Laboratory Value
AST (SGOT) and ALT (SGPT)	≤ 2.5 × ULN
Coagulation	
International normalized ratio (INR) OR prothrombin time (PT) and activated partial thromboplastin time (aPTT)	≤ 1.5 × ULN unless participant is receiving anticoagulant therapy as long as PT or aPTT is within therapeutic range of intended use of anticoagulants

ALT (SGPT)=alanine aminotransferase (serum glutamic pyruvic transaminase); AST (SGOT)=aspartate aminotransferase (serum glutamic oxaloacetic transaminase); GFR=glomerular filtration rate; ULN=upper limit of normal.

[a] Criteria must be met without erythropoietin dependency and without packed red blood cell (pRBC) transfusion within the last 2 weeks.

[b] CrCl should be calculated per institutional standard.

5.1.2 Participant exclusion criteria

Participants are excluded from the study if any of the following criteria apply:

1. Participants with involved retroperitoneal (e.g. para-aortal, paracaval or interaortocaval lymph nodes) or mesenterial lymph nodes (distant metastasis!)
2. A WOCBP who has a positive urine pregnancy test within 72 hours prior to start of study intervention (see Appendix 2). If the urine test is positive or cannot be confirmed as negative, a serum pregnancy test will be required.
3. Received prior therapy with an anti-PD-1, anti-PD-L1, or anti-PD-L2 agent or with an agent directed to another stimulatory or co-inhibitory T-cell receptor (e.g., CTLA-4, OX-40, CD137).
4. Participant received colony-stimulating factors (e.g. granulocyte colony-stimulating factor [G-CSF], granulocyte-macrophage colony-stimulating factor [GM-CSF] or recombinant erythropoietin) within 28 days prior to the first dose of study intervention.
5. Major surgery within 2 weeks of starting study intervention and patients must have recovered from any effects of any major surgery.
6. Concomitant use of drugs inhibiting (dihydropyrimidine dehydrogenase) DPD activity (including sorivudine, brivudine), the required wash out phase is 4 weeks before start of the study intervention.
7. Inadequate cardiac function (LVEF value < 55 %) as determined by echocardiography
8. Resting ECG indicating uncontrolled, potentially reversible cardiac conditions, as judged by the investigator (e.g., unstable ischemia, uncontrolled symptomatic arrhythmia, congestive heart failure, QTcF prolongation > 500 ms, electrolyte disturbances, etc.), or patients with congenital long QT syndrome.

9. Participant has received a live vaccine or live-attenuated vaccine within 30 days prior to the first dose of study drug. Administration of killed vaccines is allowed.
10. Participant is currently participating in or has participated in a study of an investigational agent within 4 weeks or within less than 5 half-lives of the investigational agent (whichever is longer) or has used an investigational device within 4 weeks prior to the first dose of study intervention.
11. Participant has a diagnosis of immunodeficiency or is receiving chronic systemic steroid therapy (in dosing exceeding 10 mg daily of prednisone equivalent) or any other form of immunosuppressive therapy within 7 days prior to the first dose of study drug.
12. Participant has a known additional malignancy that is progressing or has required active treatment within the past 2 years. Participants with basal cell carcinoma of the skin, squamous cell carcinoma of the skin or carcinoma in situ (e.g., breast carcinoma, cervical cancer in situ) that have undergone potentially curative therapy are not excluded.
13. Participant has myelodysplastic syndrome (MDS)/acute myeloid leukemia (AML) or with features suggestive of MDS/AML.
14. Participant has severe dyspnea at rest requiring supplementary oxygen therapy.
15. History of severe allergic, anaphylactic, or other hypersensitivity reactions to chimeric or humanized antibodies or fusion protein; known hypersensitivity to Chinese hamster ovary cell products or to any component of the pembrolizumab or trastuzumab formulation
16. Any known contraindication (including hypersensitivity) to docetaxel, 5-FU, folinic acid/leucovorin, or oxaliplatin.
17. Known DPD deficiency. Patients with a reduced DPD activity (CPIC activity score of 1.0-1.5) might participate in the study and receive a reduced dosage of 5-FU after discussion with the coordinating investigator and sponsor [https://cpicpgx.org/guidelines/guideline-for-fluoropyrimidines-and-dpyd/]
18. Participant has active autoimmune disease that has required systemic treatment in the past 2 years (i.e., with use of disease modifying agents, corticosteroids or immunosuppressive drugs). Replacement therapy (e.g., thyroxine, insulin, or physiologic corticosteroid replacement therapy for adrenal or pituitary insufficiency, etc.) is not considered a form of systemic treatment and is allowed.
19. Participant has a history of (non-infectious) pneumonitis/interstitial lung disease that required steroids or has current pneumonitis/interstitial lung disease.
20. Participant has an active infection requiring systemic therapy.
21. Participant has a known history of Human Immunodeficiency Virus (HIV) infection

22. Participant has a known history of Hepatitis B (defined as Hepatitis B surface antigen [HBsAg] reactive) or known active Hepatitis C virus (defined as HCV RNA is detected) infection.
23. Participant is considered a poor medical risk due to a serious, uncontrolled medical disorder, non-malignant systemic disease or active, uncontrolled infection. Examples include, but are not limited to, uncontrolled ventricular arrhythmia, recent (within 3 months) myocardial infarction, uncontrolled major seizure disorder, unstable spinal cord compression, superior vena cava syndrome, extensive interstitial bilateral lung disease on High Resolution Computed Tomography (HRCT) scan, previous allogenic bone marrow/blood transplantation or any psychiatric disorder or substance abuse that prohibits obtaining informed consent.
24. Participant is pregnant or breastfeeding or expecting to conceive or father children within the projected duration of the study, starting with the screening visit through 6 months after the last dose of study intervention.
25. Participant has had an allogenic tissue/solid organ transplant.

5.1.3 Lifestyle considerations

5.1.3.1 Meals and dietary restrictions

Participants should maintain a normal diet unless modifications are required to manage an AE such as diarrhea, nausea or vomiting.

5.1.3.2 Activity restrictions

Adverse events related to chemotherapy, pembrolizumab or trastuzumab may include asthenia, fatigue and dizziness. Therefore, participants should be advised to use caution while driving or using machinery if these symptoms occur.

5.1.3.3 Contraception

FLOT chemotherapy, pembrolizumab and trastuzumab may have adverse effects on a fetus in utero. Refer to Appendix 2 for approved methods of contraception.

For this study, male participants will be considered to be of non-reproductive potential if they have azoospermia (whether due to having had a vasectomy or due to an underlying medical condition).

5.1.4 Pregnancy

If a participant inadvertently becomes pregnant while on study intervention, the participant will be immediately discontinued from study intervention(s). The site will contact the participant at

least monthly and document the participant's status until the pregnancy has been completed or terminated. The outcome of the pregnancy will be reported to the Sponsor or designee (who will forward the report to MSD Pharmacovigilance Germany and ORGANON's Local Pharmacovigilance within 1 working day) if the outcome is a serious adverse experience (e.g., death, abortion, congenital anomaly, or other disabling or life-threatening complication to the mother or newborn). The study Investigator will make every effort to obtain permission to follow the outcome of the pregnancy and report the condition of the fetus or newborn to the Sponsor or designee. If a male participant impregnates his female partner, the study personnel at the site must be informed immediately and the pregnancy must be reported to the Sponsor or designee and followed as described in Section 6.2.

Please also note that women should not breast-feed during trastuzumab therapy and for 7 months after the last dose.

5.2 Trial intervention(s)

The intervention(s) to be used in this trial is outlined below in Table 3.

Table 3 Trial interventions

Drug	Dose/Potency	Duration of administration	Route of Administration	Day(s) of application*
**Perioperative Chemo-immunotherapy Phase (8 weeks pre- and 8 weeks post-surgery)*:**				
**Pembrolizumab**	200 mg	30 min	IV Infusion	d1, d22, d43
**Trastuzumab**	8 mg/kg (loading dose)<br>6 mg/kg	90 min<br>30 min	IV Infusion<br>IV Infusion	d1<br>d22, d43
**FLOT:**<br>**Oxaliplatin**<br>**Folinic Acid****<br>**5-FU*****<br>**Docetaxel**	 85 mg/m²<br>200 mg/m²<br>2600 mg/m² *<br>50 mg/m²	 2 h<br>1 h<br>24 h<br>1 h	 IV Infusion<br>IV Infusion<br>IV Infusion<br>IV Infusion	 d1, d15, d29, d43
**Post Chemotherapy phase (for up to 33 weeks/ 11 cycles):**				
**Pembrolizumab**	200 mg	30 min	IV Infusion	d1 of each 3-week cycle
**Trastuzumab**	6 mg/kg	30 min	IV Infusion	d1 of each 3-week cycle

*Therapy can also be administered over two days, administering pembrolizumab/trastuzumab on first day and FLOT on following day at timepoints where combination is planned. Infusion rates of chemotherapeutical components might be modified according to local standards.

**Folinic acid can be applied according to local standards (product and dosing).

***Dosage in DPD mutation carriers with a CPIC activity score of 1.0-1.5 should be reduced by 50%. See section 5.2 for dose adjustments in patients with a reduced DPD activity.

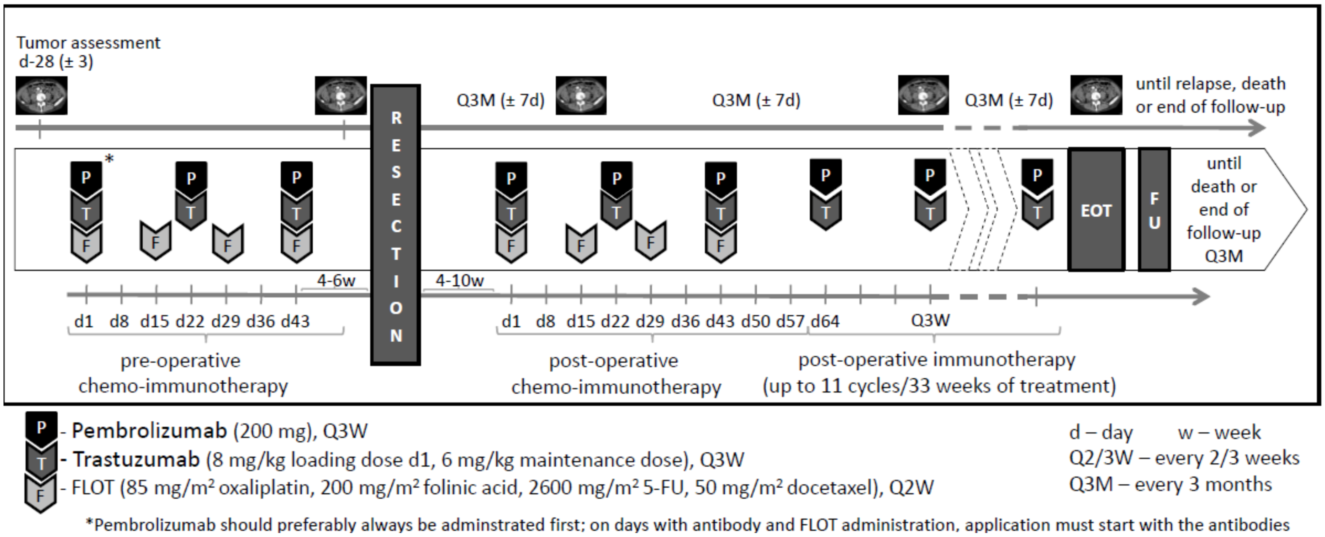

Figure 2 Treatment overview

5.2.1 Timing of dose administration

Trial interventions should be administered after all necessary procedures/assessments have been completed as detailed on the Schedule of Activities (Section 2.3). All eligible patients will receive pembrolizumab in combination with trastuzumab every 3 weeks and FLOT chemotherapy every 2 weeks pre-operative for a duration of 8 weeks. Surgical resection will follow 4-6 weeks after last dose of pre-operative treatment the earliest. 4-10 weeks after surgery 8 weeks post-operative treatment with pembrolizumab in combination with trastuzumab every 3 weeks and FLOT chemotherapy every 2 weeks will start. Afterwards maintenance therapy with pembrolizumab in combination with trastuzumab every 3 weeks for up to 11 cycles /33 weeks of treatment. In total each patient will receive approximately one year of systemic treatment with max. 17 cycles of trastuzumab/pembrolizumab administration (). In case post-operative therapy did not start within at last 10 weeks after surgery, the study treatment ends (EOT) and the patient enters follow-up phase.

All trial interventions will be administered on an outpatient basis.

Pembrolizumab 200 mg will be administered as a 30 minute IV infusion every 3 weeks. Sites should make every effort to target infusion timing to be as close to 30 minutes as possible.

However, given the variability of infusion pumps from site to site, a window of -5 minutes and +10 minutes is permitted (i.e., infusion time is 30 minutes: -5 min/+10 min).

The Pharmacy Manual contains specific instructions for the preparation of the pembrolizumab infusion fluid and administration of infusion solution.

Trastuzumab will be administered at 8 mg/kg initial loading dose (D1 pre- and D1 post-operative), following doses of 6 mg/kg every 3 weeks. If the patient missed a dose by a week or less, the usual maintenance dose should be given as soon as possible. If the patient missed a dose by more than a week, another initial dose should be given as soon as possible. Subsequent maintenance doses should be administered according to the 3 weeks interval after 21 days.

On days with both monoclonal antibody and chemotherapy application therapy must always start with monoclonal antibodies. In addition, pembrolizumab should be preferably administrated first if multiple agents are delivered the same day.

In patients with a DPD mutation resulting in a reduced DPD activity with a CPIC activity score of 1.0-1.5 fluoropyrimidine dosage should be reduced by 50%. Patients with a CPIC activity score of 0-0.5 cannot participate in the current study.

If a patient discontinues the FLOT chemotherapy completely or partially, the patient might continue to receive pembrolizumab and trastuzumab. If a patient has to discontinue pembrolizumab, the patient is allowed to continue to receive trastuzumab and vice versa. If both of the antibodies need to be permanently discontinued, the patient is allowed to continue study treatment with FLOT.

5.2.2 Prerequisites for application of study intervention

Patients can start with the application of the corresponding study intervention (FLOT plus pembrolizumab and trastuzumab) if the following prerequisites are met:

- Leucocytes ≥ 3,000/µL*
- Thrombocytes ≥ 100,000/µL
- no relevant non-hematologic toxicity ≥ Grade 2.
- Serum bilirubin ≤ 1.5 x ULN (≤ 3.0 x ULN in patients with known M. Meulengracht).
- Creatinine clearance ≥ 50mL/min (calculated according to Cockcroft and Gault)
-

*Note: For FLOT only therapy, only the first three parameters apply (leucocytes, thrombocytes, non-hematologic toxicity). For FLOT, the overall number of leukocytes is relevant, not the neutrophils. Treatment can be continued if leukocytes are at 3.000/µl or higher, independently of the number of

neutrophils – provided the patient has no infection, fever or other side effects that are possibly neutropenia-related

5.2.3 Dose modification and toxicity management of FLOT chemotherapy.

Toxicity will be graded according to NCI CTCAE, version 5.0. The dose modification described below is performed according to this grading system. Toxicities of severity grade 1 only will not lead to any dose reduction or administration delay. The same holds for adverse reactions without any potential of serious or life-threatening complications according to the judgment of the physician (e.g. alopecia). In case of toxicities requiring dose modification, the dose modification should reflect the causal relationship to the respective drug(s). E.g., if the toxicity is unequivocally caused by only one drug, a dose modification of other drugs is not required. If more than one different type of toxicity occurs concurrently, the most severe grade will determine the modification. If a dose reduction is performed, the reduced dose level is usually kept throughout the rest of the study without re-escalation. However, investigators can deviate from this procedure and increase the dose to the previous level if they feel i) that this is in the best interest of the patient and if they ii) do not expect the toxicity to reoccur.

In case of acute allergic reactions of grade 3 or 4, the causative agent should be discontinued permanently. In case of grade 1 or 2, it is up to the physician to continue treatment without dose modification, if this is in the best interest of the patient.

The study teams will document all actions of dose modifications or treatment delays in the eCRF, including the reason for each action**.**

Hematologic toxicity:

Patients who experience febrile neutropenia (despite the use of G-CSF), or thrombocytopenia causing bleeding, or any other hematological dose limiting toxicities (DLT, investigator decision) will receive a dose reduction of docetaxel and oxaliplatin to 75% of the initial dose. Subsequent dose limiting toxicities lead to a further dose reduction to 50% of the initial dose level. If DLTs reoccur at the 50% dose level, the investigator may remove one or both of the drugs (investigator decision)

Oxaliplatin-related neurotoxicity:

Table 4 Dose adjustment in case of oxaliplatin-related neurotoxicity

**Neurotoxicity**	**Duration of neurotoxicity**		
	≤ 7 days	> 7 and < 14 days	Persistent (present at start of next cycle)
cold-induced dysesthesia	no change	no change	no change
Paresthesia	no change	no change	reduction to 75%
paranesthesia with pain	no change	reduction to 75%	stop oxaliplatin*, continue docetaxel/5-FU/FA

paresthesia with functional impairment	no change	reduction to 50%	stop oxaliplatin*, continue docetaxel/5-FU/FA

*Usually, discontinuation will be permanently. Nevertheless, administration of oxaliplatin can be resumed (e.g. after complete recovery from the related symptoms) if the investigator decides that this is in the best interest of the patient and if he does not expect the toxicity to reoccur. FA – Folinic Acid, 5-FU – 5-Fluorouracil

Renal toxicity:
If a creatinine clearance of < 30 mL/min is measured, oxaliplatin treatment will be stopped. 5-FU and docetaxel administration can be continued under consideration of the recommendations given in the respective SmPCs.

Other toxicities:
If non-hematologic toxicities ≥ grade 3 appear, the dose of the chemotherapeutic agent most likely responsible for the observed toxicity should be reduced to 75% of the initial dose (for all further administrations). This procedure may apply to grade 2 toxicities upon investigator decision. In case of repeated toxicity, a further dose reduction to 50% of the initial dose should be performed. If the toxicity re-occurs at the 50% dose level, the investigator should remove the relevant drug(s) or stop the whole treatment. Each of the chemotherapeutic agents administered in the course of this study are market approved in Germany and all participating investigators will have broad experience with these medications. Therefore, investigators are permitted to deviate from the recommendations given above in reasonable cases.

5.2.4 Dose Modification and toxicity management for immune-related AEs associated with pembrolizumab and combination therapy

AEs associated with pembrolizumab exposure, including coadministration with additional compounds such as FLOT and trastuzumab, may represent an immunologic etiology. These immune-related AEs (irAEs) may occur shortly after the first dose or several months after the last dose of pembrolizumab treatment and may affect more than one body system simultaneously. Therefore, early recognition and initiation of treatment is critical to reduce complications. Based on existing clinical study data, most irAEs were reversible and could be managed with interruptions of pembrolizumab treatment, administration of corticosteroids and/or other supportive care. For suspected irAEs, ensure adequate evaluation to confirm etiology or exclude other causes. Additional procedures or tests such as bronchoscopy, endoscopy, skin biopsy may be included as part of the evaluation. Dose modification and toxicity management guidelines for irAEs associated with pembrolizumab treatment are provided in table 5.

Attribution of Toxicity:
Presumably, severe overlapping toxicity between pembrolizumab, trastuzumab and/or FLOT will not occur. Thus, in case of toxicity requiring treatment modification, this alteration should

reflect the causal relationship of the respective drug(s). For example, if the toxicity is unequivocally caused by only pembrolizumab, a dosage modification of the other drugs is not required.

Holding study interventions:

When study interventions are administered in combination, if the AE is considered immune-related, pembrolizumab should be held according to recommended dose modifications and trastuzumab with chemotherapy may be continued at the principal investigator’s discretion.

Restarting study interventions:

Participants may not have any dose modifications (no change in dose or schedule) of pembrolizumab in this study, as described in Table 5.

- If pembrolizumab application has to be stopped permanently due to an irAE, administration of chemotherapy and/or trastuzumab may continue if the AE was not related to the chemotherapeutical components or trastuzumab.
- If the toxicities do resolve and conditions are aligned with what is defined in table 5, the combination of pembrolizumab and trastuzumab and chemotherapy may be restarted at the discretion of the investigator. In these cases where the toxicity is attributed to the combination or to chemotherapy or trastuzumab alone, re-initiation of pembrolizumab as a monotherapy may be considered at the principal investigator’s discretion.

General instructions:

- Severe and life-threatening irAEs should be treated with IV corticosteroids followed by oral steroids. Other immunosuppressive treatment should begin if the irAEs are not controlled by corticosteroids.
- Pembrolizumab must be permanently discontinued if the irAE does not resolve or the corticosteroid dose is not ≤10 mg/day within 12 weeks of the last study intervention treatment. Chemotherapy or trastuzumab administration may be continued at the discretion of the principal investigator if the AE is solely related to pembrolizumab
- The corticosteroid taper should begin when the irAE is ≤ grade 1 and continue at least 4 weeks.
- If study intervention has been withheld, study intervention may resume after the irAE decreased to ≤ Grade 1 after corticosteroid taper.

Table 5 Dose modification and toxicity management guidelines for immune-related AEs associated with pembrolizumab monotherapy and IO Combinations

irAEs	Toxicity grade CTCAE v5.0	Action with Pembrolizumab	Corticosteroid and/or other therapies	Monitoring and Follow-up
Pneumonitis	Grade 2	Withhold	• Administer corticosteroids (initial dose of 1 to 2 mg/kg prednisone or equivalent) followed by taper<br>• Add prophylactic antibiotics for opportunistic infections	• Monitor participants for signs and symptoms of pneumonitis<br>• Evaluate participants with suspected pneumonitis with radiographic imaging and initiate corticosteroid treatment
	Recurrent Grade 2, Grade 3 or 4	Permanently discontinue		
Diarrhea/ Colitis	Grade 2 or 3	Withhold	• Administer corticosteroids (initial dose of 1 to 2 mg/kg prednisone or equivalent) followed by taper	• Monitor participants for signs and symptoms of enterocolitis (ie, diarrhea, abdominal pain, blood or mucus in stool with or without fever) and of bowel perforation (ie, peritoneal signs and ileus)<br>• Participants with ≥ grade 2 diarrhea suspecting colitis should consider GI consultation and performing endoscopy to rule out colitis<br>• Participants with diarrhea/colitis should be advised to drink liberal quantities of clear fluids. If sufficient oral fluid intake is not feasible, fluid and electrolytes should be substituted via IV infusion
	Recurrent Grade 3 or Grade 4	Permanently discontinue		
AST or ALT elevation or increased bilirubin	Grade 2[a]	Withhold	• Administer corticosteroids (initial dose of 0.5 to 1 mg/kg prednisone or equivalent) followed by taper	• Monitor with liver function tests (consider weekly or more frequently until liver enzyme value returned to baseline or is stable)
	Grade 3[b] or 4[c]	Permanently discontinue	• Administer corticosteroids (initial dose of 1 to 2 mg/kg prednisone or equivalent) followed by taper	
T1DM or Hyper-glycemia	New onset T1DM or Grade 3 or 4	Withhold[d]	• Initiate insulin replacement therapy for participants with T1DM	• Monitor participants for hyperglycemia or other signs and symptoms of diabetes

irAEs	Toxicity grade CTCAE v5.0	Action with Pembrolizumab	Corticosteroid and/or other therapies	Monitoring and Follow-up
	hyperglycemia associated with evidence of β-cell failure		• Administer antihyperglycemic in participants with hyperglycemia	
Hypophysitis	Grade 2	Withhold	• Administer corticosteroids and initiate hormonal replacements as clinically indicated	• Monitor for signs and symptoms of hypophysitis (including hypopituitarism and adrenal insufficiency)
	Grade 3 or 4	Withhold or permanently discontinue[d]		
Hyper-thyroidism	Grade 2	Continue	• Treat with nonselective beta-blockers (e.g. propranolol) or thionamides as appropriate	• Monitor for signs and symptoms of thyroid disorders
	Grade 3 or 4	Withhold or permanently discontinue[d]		
Hypo-thyroidism	Grade 2, 3 or 4	Continue	• Initiate thyroid replacement hormones (e.g. levothyroxine or liothyronine) per standard of care	• Monitor for signs and symptoms of thyroid disorders
Nephritis: grading according to increased creatinine or acute kidney injury	Grade 2	Withhold	• Administer corticosteroids (prednisone 1 to 2 mg/kg or equivalent) followed by taper	• Monitor changes of renal function
	Grade 3 or 4	Permanently discontinue		
Neurological Toxicities	Grade 2	Withhold	• Based on severity of AE administer corticosteroids	• Ensure adequate evaluation to confirm etiology and/or exclude other causes
	Grade 3 or 4	Permanently discontinue		

irAEs	Toxicity grade CTCAE v5.0	Action with Pembrolizumab	Corticosteroid and/or other therapies	Monitoring and Follow-up
Myocarditis	Grade 1	Withhold	• Based on severity of AE administer corticosteroids	• Ensure adequate evaluation to confirm etiology and/or exclude other causes
	Grade 2, 3 or 4	Permanently discontinue		
Exfoliative Dermatologic Conditions	Suspected SJS, TEN, or DRESS	Withhold	• Based on severity of AE administer corticosteroids	• Ensure adequate evaluation to confirm etiology or exclude other causes
	Confirmed SJS, TEN, or DRESS	Permanently discontinue		
All Other irAEs	Persistent Grade 2	Withhold	• Based on severity of AE administer corticosteroids	• Ensure adequate evaluation to confirm etiology or exclude other causes
	Grade 3	Withhold or discontinue based on the event [e]		
	Recurrent Grade 3 or Grade 4	Permanently discontinue		

ALT = alanine aminotransferase; AST = aspartate aminotransferase; DRESS = drug rash with eosinophilia and systemic symptom; GI = gastrointestinal; SJS = Stevens-Johnson Syndrome; T1DM = type 1 diabetes mellitus; TEN = toxic epidermal necrolysis; ULN = upper limit of normal.

Note: Non-irAE will be managed as appropriate, following clinical practice recommendations.

[a] AST/ALT: >3.0 to5.0 x ULN if baseline normal; >3.0 to 5.0 x baseline, if baseline abnormal; bilirubin:>1.5 to 3.0 x ULN if baseline normal; >1.5 to 3.0 x baseline if baseline abnormal

[b] AST/ALT: >5.0 to 20.0 x ULN, if baseline normal; >5.0 to 20.0 x baseline, if baseline abnormal; bilirubin:>3.0 to 10.0 x ULN if baseline normal; >3.0 to 10.0 x baseline if baseline abnormal

[c] AST/ALT: >20.0 x ULN, if baseline normal; >20.0 x baseline, if baseline abnormal; bilirubin: >10.0 x ULN if baseline normal; >10.0 x baseline if baseline abnormal

[d] The decision to withhold or permanently discontinue pembrolizumab is at the discretion of the investigator or treating physician. If control achieved or ≤ grade 2, pembrolizumab may be resumed.

[e] Events that require discontinuation include, but are not limited to, encephalitis and other clinically important irAEs (e.g. vasculitis and sclerosing cholangitis).

Dose modification and toxicity management of infusion-reactions related to pembrolizumab

Pembrolizumab may cause severe or life-threatening infusion-reactions including severe hypersensitivity or anaphylaxis. Signs and symptoms usually develop during or shortly after drug infusion and generally resolve completely within 24 hours of completion of infusion. Dose modification and toxicity management guidelines on pembrolizumab associated infusion reaction are provided in table 6.

Table 6 Pembrolizumab Infusion reaction dose modification and treatment guidelines

NCI CTCAE Grade	Treatment	Premedication at Subsequent Dosing
**Grade 1**<br>Mild reaction, infusion interruption not indicated, intervention not indicated	Increase monitoring of vital signs as medically indicated until the participant is deemed medically stable in the opinion of the investigator.	None
**Grade 2**<br>Requires therapy or infusion interruption but responds promptly to symptomatic treatment (e.g., antihistamines, NSAIDs, narcotics, IV fluids); prophylactic medications indicated for ≤ 24 h	**Stop Infusion.**<br>Additional appropriate medical therapy may include but is not limited to: IV fluids, antihistamines, NSAIDs, acetaminophen, narcotics.<br>Increase monitoring of vital signs as medically indicated until the participant is deemed medically stable in the opinion of the investigator.<br>If symptoms resolve within 1 h of stopping drug infusion, the infusion may be restarted at 50% of the original infusion rate (e.g., from 100 mL/h to 50 mL/h). Otherwise, dosing will be held until symptoms resolve and the participant should be premedicated for the next scheduled dose.<br>**Participants who develop grade 2 toxicity despite adequate premedication should be permanently discontinued from further study drug intervention**	Participant may be premedicated 1.5 h (± 30 min) prior to infusion of study intervention with:<br>Diphenhydramine 50 mg po (or equivalent dose of antihistamine).<br>Acetaminophen 500-1000 mg po (or equivalent dose of analgesic).
**Grade 3:**<br>Prolonged (i.e., not rapidly responsive to symptomatic	**Stop Infusion.**<br>Additional appropriate medical therapy may include but is not limited to:	No subsequent dosing

medication and/or brief interruption of infusion); recurrence of symptoms following initial improvement; hospitalization indicated for other clinical sequelae (e.g., renal impairment, pulmonary infiltrates)<br>**Grade 4:**<br>Life-threatening; pressor or ventilatory support indicated	Epinephrine**, IV fluids, antihistamines, NSAIDs, acetaminophen, narcotics, oxygen, pressors, corticosteroids<br>Increase monitoring of vital signs as medically indicated until the participant is deemed medically stable in the opinion of the investigator.<br>Hospitalization may be indicated.<br>**In cases of anaphylaxis, epinephrine should be used immediately.<br>**Participant is permanently discontinued from further study drug intervention.**	

Appropriate resuscitation equipment should be available at the bedside and a physician readily available during the period of drug administration.
For further information, please refer to the CTCAE v5.0 at http://ctep.cancer.gov

Other allowed dose interruption for pembrolizumab

Pembrolizumab may be interrupted for situations other than treatment-related AEs such as medical / surgical events and/or unforeseen circumstances not related to study intervention. However, intervention is to be restarted within 3 weeks of the originally scheduled dose and within 42 days of the previously administered dose, unless otherwise discussed with the Sponsor. The reason for study intervention interruption is to be documented in the patient's study record.

If pembrolizumab has to be permanently discontinued due to toxicity attributed to pembrolizumab only, it is allowed to continue with administration of trastuzumab alone or in combination with FLOT within the trial.

5.2.5 Dose modifications and toxicity management for AEs associated with trastuzumab and combination therapy

Dose delays or discontinuations due to cardiac events

In this study, all patients must have a baseline left ventricular ejection fraction (LVEF) value ≥ 55 %, and LVEF is to be monitored at least prior to surgery and after the end of postoperative chemotherapy, every 12 weeks during antibody treatment and thereafter until 2 years. To ensure patient safety, if an investigator assesses that an AE may be related to cardiac dysfunction, an additional LVEF measurement should be performed, as well as other appropriate procedures such as chest X-ray, and the scheduled cardiac toxicity assessments will continue unchanged. If symptomatic LVSD (CHF) is confirmed by a cardiologist's evaluation in any patient, trastuzumab should be permanently discontinued, and the patient should be discontinued from study treatment on account of unacceptable toxicity. Investigators should take into account the potential differential diagnosis of an autoimmune myocarditis, in case of suspicion myocardial biopsy may be considered.

Infusion-associated reactions (IAR)

Administration of trastuzumab may result in infusion-associated symptoms such as nausea, pyrexia, diarrhea, chills, fatigue, and headache, or allergic reactions. The most hypersensitivity reactions were mild or moderate in severity and resolved upon treatment. Careful consideration must be given to the risk of enrolling patients with chronic lung diseases in this study.

Study treatment will be administered in a clinical treatment setting with emergency equipment and staff who are trained to monitor for and respond to medical emergencies. Any patient who experiences a grade 4 allergic reaction, bronchospasm, or acute respiratory distress syndrome (ARDS) associated with trastuzumab administration will stop treatment with trastuzumab. Patients will continue FLOT and pembrolizumab if clinically feasible and/or will be followed for clinical outcomes.

Patients who experience infusion-associated symptoms may be managed by slowing or interrupting the trastuzumab infusion and by providing supportive care with oxygen and medications (e.g., beta-agonists, antihistamines, antipyretics, corticosteroids), as determined by the investigator to be clinically appropriate.

Premedication with antipyretics, antihistamines, or corticosteroids may be administered before infusions of trastuzumab.

Any patient who experiences infusion-associated symptoms should be monitored under clinical observation until complete resolution of all signs and symptoms of the IAR.

On very rare occasions, patients have experienced the onset of infusion symptoms or pulmonary symptoms more than 6 hours after the start of the trastuzumab infusion. Patients should be

warned of the possibility of such a late onset and should be instructed to contact their physician if these symptoms occur.

If a patient cannot tolerate trastuzumab infusions, trastuzumab treatment will be permanently discontinued.

Other allowed dose interruption for trastuzumab

If a patient receives an incomplete dose of trastuzumab because of IARs or another reason, the following guidelines should apply: The patient should receive at least 50% of the dose during the first week of the treatment cycle.

Therefore, if the patient receives < 50% of the dose, the patient should receive the remainder before day 22, preferably within the first week. Thereafter, the patient should receive the usual maintenance dose 3 weeks after the first interrupted dose as routinely scheduled.

- If the patient has received 50% - 75% of the dose during the first week of the treatment cycle, the remainder should be given during the same treatment cycle (i.e., before day 22 of that cycle), preferably before the end of the second week (i.e., before day 15)
- If the patient received > 75% of the dose, the remainder should be given during the same treatment cycle (i.e., before day 22 of that cycle). Alternatively, the patient may be given a reloading dose on day 1 of the following treatment cycle.
- Refer to section 5.2.1 for guidance on reloading in the event of missed trastuzumab dose

5.3 Concomitant medications/vaccinations (allowed & prohibited)

Medications or vaccinations specifically prohibited in the exclusion criteria are not allowed during the ongoing trial. If there is a clinical indication for one of these or other medications or vaccinations specifically prohibited during the trial, discontinuation from trial therapy or vaccination may be required. The final decision on any supportive therapy or vaccination rests with the investigator and/or the participant's primary physician. However, the decision to continue the participant on study intervention requires the mutual agreement of the investigator, the Sponsor, and the participant.

5.3.1 Acceptable concomitant medications

All treatments that the investigator considers necessary for a participant’s welfare may be administered at the discretion of the investigator in keeping with the community standards of medical care. All concomitant medication will be recorded on the electronic case report form (eCRF) including all prescription, over-the-counter (OTC), herbal supplements, and IV medications and fluids. If changes occur during the trial period, documentation of drug dosage, frequency, route, and date may also be included on the eCRF.

Antiemetic therapy during FLOT chemotherapy might be applied according to international guidelines (ASCO, MASCC).

All concomitant medications received within 28 days prior to the first dose of trial intervention and up to 30 days after the last dose of trial intervention should be recorded. If participants experience an SAE or ECI, concomitant medications administered 30 days after the last dose of trial intervention are to be recorded as defined in Section 6.2.

5.3.2 Prohibited concomitant medications

Participants are prohibited from receiving the following therapies during the screening and treatment phase (including retreatment for post-complete response relapse) of this trial:

- Antineoplastic systemic chemotherapy or biological therapy
- Immunotherapy not specified in this protocol
- Chemotherapy not specified in this protocol
- Investigational agents other than pembrolizumab or trastuzumab
- Radiation therapy
- Live vaccines within 30 days prior to the first dose of study intervention and while participating in the study. Examples of live vaccines include, but are not limited to, the following: measles, mumps, rubella, varicella/zoster, yellow fever, rabies, BCG, and typhoid vaccine. Seasonal influenza vaccines for injection are generally killed virus vaccines and are allowed; however, intranasal influenza vaccines (e.g., FluMist®) are live attenuated vaccines and are not allowed.
- Systemic glucocorticoids for any purpose other than to modulate symptoms from an ECI of suspected immunologic etiology. The use of physiologic doses of corticosteroids may be approved after consultation with the Sponsor.
- Given the significant interaction with 5-FU the following drugs are prohibited:
    - Uracil, cimetidine, dipyridamole (may enhance 5-FU activity)
    - Sorivudine or its chemically related analogues such as brivudine irreversibly inhibit DPD, resulting in a significant increase in 5-FU exposure. This may lead to increased clinically significant fluoropyrimidine-related toxicities with potentially fatal outcomes
    - Phenytoin
    - Flucytosine, a fluorinated pyrimidine antifungal agent

Participants who, in the assessment by the investigator, require the use of any of the aforementioned treatments for clinical management should be removed from the study. All treatments that the investigator considers necessary for a participant’s welfare may be administered at the discretion of the investigator in keeping with the community standards of medical care.
There are no prohibited concomitant therapies during the Post-Treatment Follow-up Phase.

Note: Initiation of a new anti-cancer treatment is NOT allowed before disease progression/relapse (if patient has EOT without progression/relapse and tumor imaging is still ongoing).

5.3.3 Rescue medications & supportive care

Participants should receive appropriate supportive care measures as deemed necessary by the treating investigator. Suggested supportive care measures for the management of AEs with potential immunologic etiology are outlined along with the dose modification guidelines in Section 5.2.4 to 5.2.5. Where appropriate, these guidelines include the use of oral or IV treatment with corticosteroids, as well as additional anti-inflammatory agents if symptoms do not improve with administration of corticosteroids. Note that several courses of steroid tapering may be necessary as symptoms may worsen when the steroid dose is decreased. For each disorder, attempts should be made to rule out other causes such as metastatic disease or bacterial or viral infection, which might require additional supportive care. The treatment guidelines are intended to be applied when the investigator determines the events to be related to pembrolizumab and/or trastuzumab.

Note: If after the evaluation of the event, it is determined not to be related to pembrolizumab and/ or trastuzumab the investigator does not need to follow the treatment guidance. Refer to Section 5.2.4 – 5.2.5 for guidelines regarding dose modification and supportive care.

It may be necessary to perform conditional procedures such as bronchoscopy, endoscopy, or skin photography as part of evaluation of the event.

5.4 Surgery

In this chapter, the surgical intervention will be described. Surgery is planned to occur minimum 4 (but at last after 6) weeks after last FLOT/pembrolizumab/trastuzumab dose (=d43 pre-operative). The protocol specifications on surgical treatment reflect national guidelines and current expert opinion. Surgical therapy of locally advanced patients requires a highly individual approach. Aim of surgical resection is a margin-free (R0) resection of the primary tumor.

5.4.1 Resection of the primary tumor

Resectability of the primary tumor should be evaluated by a specialized visceral surgeon with experience in this type of surgery based on both, the initial clinical reports (baseline reports) and the investigations carried out after administration of preoperative treatment, including endoscopy and imaging. In case of uncertainties, surgical exploration is required. Open tumor resection represents the current standard intervention. Minimal invasive intervention is allowed.

5.4.2 Tumors of the gastroesophageal junction (GEJ I-III)

Adenocarcinomas of the esophagogastric junction (GEJ) are topographically-anatomically classified in GEJ types I-III according to Siewert and Hölscher.[42] The classification is based on endoscopic results (orthograde view on the tumor and assessment with the inversed endoscope) and CT or MRI scans with coronary section images.

GEJ type I

For radical resection of lower esophageal adenocarcinoma (GEJ type I) the right-transthoracic en-bloc-esophagectomy with radical mediastinal lymphadenectomy (LAD) as well as abdominal LAD (so-called 2-field-LAD) represents the current standard procedure. Reconstruction of the food passage will be usually achieved by gastric tube or colon interposition with high intrathoracic or cervical anastomosis.

GEJ type II (so called adenocarcinoma of the cardia) and III (adenocarcinoma of the sub-cardiac region)

For GEJ types II and III tumors, the optimal surgical procedure can often only be determined during surgery (gastrectomy vs. esophagectomy). Therefore, following to laparotomy, the esophageal hiatus shall be exposed with an intra-operative assessment of margin-free resectability at the esophagus. If a safe margin-free resection via laparotomy seems feasible, a transhiatal extended gastrectomy with distal esophagectomy is performed. In this case, a D2-LAD (LAD of levels 1 and 2) with LAD of the lower mediastinum is executed. The reconstruction after extended gastrectomy shall preferably be performed via a Roux-en-Y reconstruction with end-to-side esophagojejunostomy. The retrocolonic reconstruction uses the second jejunal loop. 50 cm distal of the esophagojejunostomy the Roux-en-Y reconstruction will be completed by a jejuno-jejunostomy end-to-side. Based on center-specific experience other techniques of reconstruction may be used.

If a margin-free resection is not feasible due to the oral expansion of the tumor into the esophagus, a thoraco-abdominal esophagectomy with proximal stomach resection and reconstruction by gastric tube is indicated, as done with GEJ type I tumors. If esophagogastrectomy is required in order to achieve margin-free resection, reconstruction is done with colon interposition

5.4.3 Gastric cancer

In patients with a gastric cancer of the upper and middle third, total gastrectomy with D2-LAD is indicated. The reconstruction technique can be chosen by the center.

In patients with a distal stomach cancer, a subtotal, distal gastrectomy may be applied. The oral safety margin should be 5 cm for the intestinal type and 8 cm for the diffuse type. LAD will be performed as modified D2-LAD.

For this modified D2-LAD, lymph nodes of level 1 (station 1-6) located directly at the stomach and lymph nodes of level 2, located in the draining area of the truncus coeliacus (station 7-12) will be removed:

- Station 7 left gastric artery
- Station 8 hepatic artery
- Station 9 coeliac trunk
- Station 10 splenic hilus (optional)
- Station 11 splenic artery

In some centers, D2-LAD also includes lymph nodes in station 12 at the hepatoduodenal ligament.

Following adequate D2-LAD, usually at least 25 lymph nodes are removed and sent to the local pathology for histopathological examination. For determination of the N-status in accordance with the current version of the UICC-classification, a minimum number of 16 lymph nodes is required.

5.4.4 Splenectomy

Whenever possible, surgeons should avoid a splenectomy. Indications for splenectomy are as follows:

Absolute indication: - direct infiltration of the tumor into the spleen

Relative indication: - huge tumors in the upper and middle third of the stomach
- linitis plastic
- huge lymphoma in the area of the spleen hilus which cannot be removed safely
- intra-operative bleeding that can only be controlled by splenectomy

5.4.5 Pancreatic resection

A pancreatic resection should be avoided. A pancreatic resection can be indicated if direct tumor infiltration into the pancreas is present. Tumor infiltration of distal stomach carcinomas into the head of the pancreas indicates a modified resection of the pancreas head according to a Whipple-approach.

5.5 Participant Discontinuation Criteria

Discontinuation of study intervention does not represent withdrawal from the study.

As certain data on clinical events beyond study intervention discontinuation may be important to the study, they must be collected through the participant's last scheduled follow-up, even if the participant has discontinued study intervention. Therefore, all participants who discontinue study intervention prior to completion of the protocol-specified treatment period will still continue to be monitored in this study and participate in the study visits and procedures as specified in Section 2.3 unless the participant has withdrawn from the study (Section 5.6).

Participants may discontinue study intervention at any time for any reason or be discontinued from the study intervention at the discretion of the investigator should any untoward effect occur. In addition, a participant may be discontinued from study intervention by the investigator or the Sponsor if study intervention is inappropriate, the study plan is violated, or for administrative and/or other safety reasons.

A participant must be discontinued from study intervention but continue to be monitored in the study for any of the following reasons:

- The participant requests to discontinue study intervention
- After prolonged study intervention interruption that prohibits restarting study intervention if agreed upon with the sponsor
- Radiographic disease progression/relapse as outlined in Section 6.1.2.8
- Any progression or recurrence of any malignancy, or any occurrence of another malignancy that requires active treatment
- Any study intervention-related toxicity specified as a reason for permanent discontinuation as defined in the guidelines for dose modification due to AEs in Section 5.2.4 to 5.2.5
- Bone marrow findings consistent with MDS or AML
- The participant has a medical condition or personal circumstance which, in the opinion of the investigator and/or sponsor, placed the participant at unnecessary risk from continued administration of study intervention.
- The participant has a confirmed positive serum pregnancy test
- Completion of in total 17 administration cycles (approximately 1 year of systemic therapy) with pembrolizumab and trastuzumab

5.6 Participant withdrawal from study

A participant must be withdrawn from the study if the participant withdraws consent from the study.

If a participant withdraws from the study, they will no longer receive study intervention or be followed at scheduled protocol visits.

Specified details regarding procedures to be performed at the time of withdrawal from the study as well as specific details regarding withdrawal from future biomedical research are outlined in Section 6.1.4.1 and Section 6.1.4.2.

5.7 Clinical criteria for early trial termination

Early trial termination will be the result of the criteria specified below:

- Quality or quantity of data recording is inaccurate or incomplete
- Poor adherence to protocol and regulatory requirements
- Incidence or severity of adverse drug reaction in this or other studies indicates a potential health hazard to participants
- Plans to modify or discontinue the development of the drugs under investigation

In the event of MSD or ORGANON decision to no longer supply study drug, ample notification will be provided to the sponsor so that appropriate adjustments to participant treatment can be made.

5.7.1 Handling premature treatment (whole treatment) termination

Therapy termination before surgery: If the tumor is still operable and resectable, the patient should undergo surgery as soon as possible. 30 days after surgery the patient will be removed from the active part of the study (end of treatment, EOT) and enter the follow-up phase. In individual cases, e.g. premature surgery because of tumor bleeding, patients can resume treatment after surgery and remain in the active study part. In case of non-resectable tumors (e.g. therapy termination due to disease progression to unresectable stage), the patient will be immediately removed from the active part of the study (EOT). Any surgical intervention occurring after EOT still have to be captured in the specific eCRF pages.

Therapy termination after surgery: If chemotherapy is prematurely discontinued during the post-operative cycles, the patient may remain in the active study part and continue treatment with trastuzumab and/or pembrolizumab at the discretion of the primary investigator.

5.7.2 Handling disease progression prior to surgery

Progressive disease prior to surgery occurs when patients experience a progression of the primary tumor (as assessed by endoscopy for example) or develop metastases prior to surgery. Investigators should consider pseudoprogression during immunotherapy as potential cause for progressive disease. Investigators will stop chemotherapeutic, trastuzumab and pembrolizumab treatment for these patients. If surgical intervention is still reasonable (investigator decision), patients may proceed to surgery and the related information will be documented in the eCRF.

After surgery, patients will be removed from the active part of the study (EOT) and will enter the follow-up phase for PFS/DFS and OS. If surgery is not possible or not recommended, patients will be removed from the active part of the study (EOT) and enter the follow-up phase for OS. The treating physician will define the subsequent therapy for this patient according to local protocols. All related data will be collected in the eCRF and follow-up will be performed for PFS/DFS and OS assessment.

5.7.3 Handling inoperability defined during surgery and R1/2 resection

If metastatic disease is diagnosed during surgery (e.g. liver metastases or peritoneal carcinomatosis), resection can be performed if considered possible and reasonable. After surgery, treatment can be continued within the study if the metastatic lesions identified are not considered as disease progression (investigator's opinion). Patients found to be non-resectable prior to or during surgery as well as patients receiving palliative resection only will be removed from the active part of the study (treatment discontinuation). The treating physician will define the subsequent therapy for these patients according to local protocols. All related data will be collected in the eCRF and follow-up will be performed for PFS and OS assessment. Any subsequent therapy (chemo- or radiation therapy) should be documented in the eCRF.

6 TRIAL ASSESSMENTS AND PROCEDURES

6.1 Trial Procedures

- Study procedures and their timing are summarized in the schedule of activities (Section 2.3). Individual trial procedures are described in detail below.
- Adherence to the study design requirements, including those specified in the schedule of activities (Section 2.3) is essential and required for study conduct.
- The investigator is responsible for ensuring that procedures are conducted by appropriately qualified (by education, training, and experience) staff.
- All screening evaluations must be completed and reviewed to confirm that potential participants meet all eligibility criteria. The investigator will maintain a screening log to record details of all participants screened and to confirm eligibility or record reasons for screening failure, as applicable.
- Additional evaluations/testing may be deemed necessary by the investigator, the Sponsor and/or MSD for reasons related to participant safety. In some cases, such evaluation/testing may be potentially sensitive in nature (e.g., HIV, Hepatitis C), and thus local regulations may require that additional informed consent be obtained from the participant. In these cases, such evaluations/testing will be performed in accordance with those regulations.

- Procedures conducted as part of the participant's routine clinical management (e.g., blood count) and obtained before signing of ICF may be utilized for screening or baseline purposes provided the procedure met the protocol-specified criteria and were performed within the time frame defined in the schedule of activities (Section 2.3).

6.1.1 Administrative and general procedures

6.1.1.1 Informed consent

The investigator must obtain documented consent from each potential participant prior to participating in a clinical trial and before any study related procedures are performed. If there are changes to a participant's status during the study (e.g., health requirements) the investigator must ensure appropriate consent is in place.

6.1.1.2 General informed consent

Consent must be documented by the participant's dated signature on a consent form along with the dated signature of the person conducting the consent discussion.

A copy of the signed and dated consent form should be given to the participant before participation in the trial.

The initial informed consent form, any subsequent revised written informed consent form and any written information provided to the participant must receive the IRB/ERC's approval/favorable opinion in advance of use. The participant should be informed in a timely manner if new information becomes available that may be relevant to the participant's willingness to continue participation in the trial. The communication of this information will be provided and documented via a revised consent form or addendum to the original consent form that captures the participant's dated signature.

Specifics about a trial and the trial population will be added to the consent form template at the protocol level.

The informed consent will adhere to IRB/ERC requirements, applicable laws and regulations and Sponsor requirements.

6.1.1.3 Inclusion/exclusion criteria

All inclusion and exclusion criteria will be reviewed by the investigator or qualified designee to ensure that the participant qualifies for the trial.

6.1.1.4 Medical History

A medical history will be obtained by the investigator or qualified designee. Medical history will include all active conditions, and any condition diagnosed within the prior 10 years that are considered to be clinically significant by the Investigator. This includes the assessment of blood transfusions within the previous 120 days from start of study intervention and the reasons, e.g. bleeding or myelosuppression. Details regarding the patient's esophagogastric adenocarcinoma for which the participant has been enrolled in this study will be recorded separately and not listed as medical history.

6.1.1.5 Prior and concomitant medications review

6.1.1.5.1 Prior medications

The investigator or qualified designee will review prior medication use, including any protocol-specified washout requirement, and record prior medication taken by the participant within 28 days before starting the trial. Prior anti-cancer treatment, if allowed by the inclusion/exclusion criteria, for the patient's esophagogastric adenocarcinoma will be recorded separately and not listed as a prior medication.

6.1.1.5.2 Concomitant medications

The investigator or qualified designee will record relevant medication, if any, taken by the participant during the trial. All medications related to reportable SAEs and ECIs should be recorded as defined in Section 6.2.

6.1.1.6 Disease details and treatments

6.1.1.6.1 Disease details

The investigator or qualified designee will obtain prior and current details regarding the patient's esophagogastric adenocarcinoma status.

6.1.1.6.2 Prior treatment details

The patients enrolled in this trial are newly diagnosed with esophagogastric adenocarcinoma and must not have received any prior antineoplastic treatment for this disease including systemic treatments, radiation and surgeries according to the inclusion//exclusion criteria.

6.1.1.6.3 Subsequent anti-cancer therapy status

The investigator or qualified designee will review all new anti-neoplastic therapy initiated after the last dose of study intervention. If a participant initiates a new anti-cancer therapy within 30

days after the last dose of study intervention, the 30-day safety follow-up visit must occur before the first dose of the new therapy. Once a new anti-cancer therapy has been initiated the participant will move into survival follow-up. Subsequent anti-cancer regimen will be recorded in the eCRF.

Note: Initiation of a new anti-cancer treatment is NOT allowed before disease progression/relapse (if patient has EOT without progression/relapse and tumor imaging is still ongoing).

6.1.1.7 Assignment of screening ID

All consented subjects will be given a unique screening ID that will be used to identify the subject for all procedures that occur prior to enrollment. Each subject will be assigned only one screening number. Screening numbers must not be re-used for different subjects.

6.1.1.8 Assignment of patient ID

All eligible subjects will be enrolled, by non-random assignment, and will receive a patient ID. The patient ID identifies the subject for all procedures occurring after enrollment. Once a patient ID is assigned to a subject, it can never be re-assigned to another subject.

A single subject cannot be assigned more than one patient ID.

6.1.1.9 Trial compliance (medication)

FLOT

Administration of FLOT will be performed according to local standards, witnessed by the investigator and/or trial staff and will be documented in the eCRF.

Pembrolizumab

The total volume of pembrolizumab infused will be compared to the total volume prepared to determine compliance with each dose of pembrolizumab administered. The instructions for preparing and administering pembrolizumab are provided in the Pharmacy Manual. Administration of trial medication will be witnessed by the investigator and/or trial staff.

Trastuzumab

The total volume of trastuzumab infused will be compared to the total volume prepared to determine compliance with each dose of trastuzumab administered. The instructions for preparing and administering trastuzumab are provided in the SmPC. Administration of trial medication will be witnessed by the investigator and/or trial staff.

6.1.2 Clinical Procedures/Assessments

6.1.2.1 Adverse Event (AE) Monitoring

The investigator or qualified designee will assess each participant to evaluate for potential new or worsening AEs as specified in the schedule of activities (Section 2.3) and more frequently if clinically indicated. AEs will be graded and recorded throughout the study and during the follow-up period according to NCI CTCAE v5.0 (see Appendix 3). Toxicities will be characterized in terms regarding seriousness, causality, toxicity grading, and action taken with regard to study intervention.

Please refer to Section 6.2 for detailed information regarding the assessment and recording of AEs.

6.1.2.2 Full Physical Exam

The investigator or qualified designee will perform a complete physical exam during the screening period within 14 days prior to first study drug administration. Clinically significant abnormal findings should be recorded as medical history. Additional full physical exams should be performed as specified in the schedule of activities (Section 2.3), i.e. before and after surgery and at EOT. Height will be measured at screening only. After the first dose of study intervention, new clinically significant abnormal findings should be recorded as AEs.
Investigators should pay attention to clinical signs related to previous serious illnesses.

6.1.2.3 Directed Physical Exam

For cycles that do not require a full physical exam as per the schedule of activities (Section 2.3), the investigator or qualified designee will perform a directed physical exam as clinically indicated prior to study intervention administration. After the first dose of study intervention, new clinically significant abnormal finding should be recorded as AEs.
Investigators should pay attention to clinical signs related to previous serious illnesses.

6.1.2.4 Vital signs

The investigator or qualified designee will take vital signs at screening, prior to the administration of each dose of study intervention and at treatment discontinuation as specified in the schedule of activities (Section 2.3). Vital signs should include temperature, pulse, respiratory rate, blood pressure and oxygen saturation. Since infusion-related reactions are known potential side effects of pembrolizumab/ trastuzumab, the patients must be closely monitored during and after infusion as described below:

First infusion

On the first infusion day, blood pressure and pulse will be measured **prior to, during and after infusion of pembrolizumab** as presented in the bullet list below (based on a 30-minute infusion)

1 Prior to the beginning of the infusion (measured once from approximately 30 minutes before up to 0 minutes [i.e., the beginning of the infusion])
2 After approximately 15 minutes during the infusion (halfway through infusion)
3 Following the end of the infusion (approximately 60 minutes ± 5 minutes)

If the infusion takes longer than 30 minutes, then blood pressure and pulse measurements should follow the principles as described above or be taken more frequently if clinically indicated. A one hour observation period is recommended after the first infusion of pembrolizumab.

Patients should be observed for at least six hours after the start of the first trastuzumab infusion for symptoms like fever and chills or other infusion-related symptoms.

Subsequent infusions

For subsequent infusions, blood pressure, pulse and other vital signs should be measured prior to the start of the infusion. Patients should be carefully monitored and blood pressure and other vital signs should be measured during and post infusion as per institution standard and as clinically indicated.

Patients should be observed for two hours after the start subsequent trastuzumab infusion for symptoms like fever and chills or other infusion-related symptoms.

Any clinically significant changes in vital signs should be documented in the source data and in the respective eCRF page.

6.1.2.5 12-Lead electrocardiogram

A standard 12-lead electrocardiogram (ECG) will be performed at screening, before surgery, at EOT and whenever clinically indicated using local standard procedures. Clinically significant abnormal findings from screening visit should be recorded as medical history, clinically significant abnormal findings during treatment should be recorded as AE.

6.1.2.6 Echocardiography

Echocardiography will be performed at screening, after surgery/before start of post-operative treatment, every 3 months during trastuzumab treatment and EOT. If treatment with trastuzumab is permanently discontinued, no further echocardiography is necessary.

6.1.2.7 Eastern Cooperative Oncology Group (ECOG) performance scale

The investigator or qualified designee will assess ECOG status (Appendix 1) at screening, prior to the administration of each dose of study intervention and discontinuation of study intervention.

6.1.2.8 Tumor imaging and assessment of disease

Tumor imaging is strongly preferred to be acquired by computed tomography (CT). For the abdomen contrast-enhanced magnetic resonance imaging (MRI) may be used when CT with iodinated contrast is contraindicated, or when local practice mandates it and if clinically appropriate. MRI is the strongly preferred modality for imaging the brain. A change between the imaging techniques is allowed as long as an assessment of the overall response or recurrency is possible. Imaging should include the chest and abdomen at baseline and all subsequent imaging time points.

Participant's eligibility will be determined using local assessment (investigator assessment) based on RECIST 1.1. In addition, images (including via other modalities) that are obtained at an unscheduled time point to determine disease progression/relapse, as well as imaging obtained for other reasons, but which demonstrate radiologic progression/relapse, should also be used to determine progression/relapse.

6.1.2.8.1 Initial tumor imaging

Initial tumor imaging at screening must be performed within 28 days (± 3 days) prior to start of study intervention. The site study team must review screening images to confirm the participant has measurable or evaluable disease per RECIST 1.1.

Tumor imaging performed as part of routine clinical management is acceptable for use as screening tumor imaging if they are suitable for baseline RECIST data collection and performed within 28 days prior to the start of study intervention.

6.1.2.8.2 Tumor imaging during the study

Tumor imaging is strongly preferred to be acquired by computed tomography (CT). For the abdomen, contrast-enhanced magnetic resonance imaging (MRI) may be used when CT with iodinated contrast is contraindicated, or when local practice mandates it and if clinically appropriate. MRI is the strongly preferred modality for imaging the brain. A change between the imaging techniques is allowed as long as an assessment of the overall response or recurrency is possible. Imaging should include the chest and abdomen at baseline and all subsequent imaging time points.

The first on study imaging assessment should be performed between the completion of the last pre-operative FLOT chemotherapy cycle/immunotherapy and surgery. Subsequent tumor

imaging should be performed every 3 months (± 7 days) or more frequently if clinically indicated. After 2 years (from EOT), imaging may be performed every 6 months.

Imaging timing should follow calendar days and should not be adjusted for delays in treatment cycle starts. Imaging should continue to be performed until disease progression/relapse is identified by the investigator.

6.1.2.8.3 End of treatment and follow-up tumor imaging

In participants who discontinue study treatment, tumor imaging should be performed at the time of treatment discontinuation (± 4 days window). If previous imaging was obtained within 4 weeks prior to the date of discontinuation, then imaging at treatment discontinuation is not mandatory. In participants who discontinue study treatment due to documented disease progression/relapse, this is the final required tumor imaging.

For participants who discontinue study treatment without documented disease progression/relapse confirmed by RECIST 1.1, tumor imaging should be performed at the time of treatment discontinuation (± 4 weeks). Then every effort should be made to continue monitoring their disease status by tumor imaging (every 3 months ± 7 days; after 2 years, every 6 months ± 7 days) to monitor disease status until the start of a new anticancer treatment, disease progression/relapse, pregnancy, death, withdrawal of consent, or the end of the study, whichever occurs first.

6.1.2.8.4 RECIST 1.1 assessment of disease

RECIST 1.1 will be used as the primary measure for assessment of tumor response, date of disease progression/relapse, and as a basis for all protocol guidelines related to disease status (e.g., discontinuation of study treatment). Although RECIST 1.1 references a maximum of 5 target lesions in total and 2 per organ.

6.1.2.9 Tumor tissue collection and correlative studies sampling

Tumor-Tissue

Archival tumor tissue sample or newly (within clinical routine) obtained core or excisional biopsy of a tumor lesion not previously irradiated should be sent to central pathology lab for optional accompanying research project (baseline sample). Formalin-fixed, paraffin embedded (FFPE) tissue blocks are preferable to slides. Newly obtained biopsies are preferred to archived tissue. Core or excisional biopsies are mandatory (fine needle aspiration and bone metastasis samples are not acceptable).

Translational Research Blood Samples

Blood samples will be taken prior to treatment, prior to second pembrolizumab administration, pre- as well as postoperatively and every 3 months afterwards until relapse/progression.

Translational Research Stool and Saliva Samples

Stool and saliva samples will be taken prior to treatment, preoperatively, directly after completion of adjuvant chemotherapy and 3 months after completion of adjuvant chemotherapy.

6.1.3 Clinical safety laboratory procedures/ assessments

Details regarding specific laboratory procedures/ assessments to be performed in this trial are provided below and timing and frequency is specified in the schedule of activities (Section 2.3).

- The investigator or medically qualified designee (consistent with local requirements) must review the laboratory report, document this review, and record any clinically relevant changes occurring during the study in the AE section of the eCRF. The laboratory reports must be filed with the source documents. Clinically significant abnormal laboratory findings are those which are not associated with the underlying disease, unless judged by the investigator to be more severe than expected for the participant's condition.
- If laboratory values from non protocol-specified laboratory assessments performed at the institution's local laboratory require a change in study participant management or are considered clinically significant by the investigator (e.g., SAE or AE or dose modification), then the results must be recorded on the appropriate eCRF page.
- For any laboratory tests with values considered clinically significantly abnormal during participation in the study or within 30 after the last dose of study intervention, every attempt should be made to perform repeat assessments until the values return to normal or baseline or if a new baseline is established as determined by the investigator.

Laboratory tests for hematology, chemistry, urinalysis, and others are specified in Table 7.

Laboratory tests for screening should be performed within 14 days prior to the first dose of study medication. In general, pre-dose laboratory procedures can be conducted up to 1 day prior to dosing in order to have the results available on the visit day. Results must be reviewed by the investigator or qualified designee and found to be acceptable prior to each dose of study intervention.

Table 7 Laboratory tests

Hematology	Chemistry	Urinalysis	Other
Hematocrit	Albumin	Blood	Serum β-human chorionic gonadotropin†
Hemoglobin	Alkaline phosphatase	Glucose	(β-hCG)†
Platelet count	Alanine aminotransferase (ALT)	Protein	PT (INR)
WBC (total and differential)	Aspartate aminotransferase (AST)	Specific gravity	aPTT
Red Blood Cell Count	Lactate dehydrogenase (LDH)	Microscopic exam *(If values of dipstick abnormal), results are noted*	Thyroid stimulating hormone (TSH)
Absolute neutrophil count	Uric acid	Urine pregnancy test†	fT3/fT4 (only if TSH is out of range)
Absolute lymphocyte count	Calcium	Nitrites	Blood for correlative studies
	Glucose	Ketones	HIV, Hepatitis B&C
	Phosphorus	Leucocytes	
	Potassium	pH	
	Sodium		
	Magnesium		
	Total bilirubin		
	Direct bilirubin *(If total bilirubin is elevated above ULN)*		
	Total protein		
	Blood urea nitrogen		
	C-reactive protein (CRP)		
	Gamma-GT		
	Creatinine, Creatinine Clearance		
	lipase		

† Perform on women of childbearing potential only. If urine pregnancy results cannot be confirmed as negative, a serum pregnancy test will be required. Alternatively, a serum pregnancy test can be performed upfront.

6.1.4 Other Procedures

6.1.4.1 Discontinuation and withdrawal

When a participant discontinues prior to trial completion, all applicable activities scheduled for the final trial visit should be performed at the time of discontinuation. Any AEs which are present at the time of discontinuation should be followed in accordance with the safety requirements outlined in Section 6.2.

Participants who withdraw from the study should be encouraged to complete all applicable activities scheduled for the final study visit at the time of withdrawal. Any AEs that are present at the time of withdrawal should be followed in accordance with the safety requirements outlined in Section 6.2.

6.1.4.2 Withdrawal from translational research / future biomedical research

Participants may withdraw their consent for future biomedical research. Participants may withdraw consent at any time by contacting the investigator. The investigator will inform the sponsor. It is the responsibility of the investigator to subsequently inform the participant of completion of withdrawal. Any analyses in progress at the time of request for withdrawal or already performed prior to the request for withdrawal being received will continue to be used as part of the overall research study data and results if not otherwise requested by the participant during the withdrawal process. No new analyses should be generated after the request is received.

In the event that the specimens have been completely anonymized, there will be no link between the participant's personal information and their specimens. In this situation, the request for specimen withdrawal cannot be processed.

6.1.4.3 Blinding/Unblinding

Not applicable, as this is an open-label trial.

6.1.5 Visit requirements

Visit requirements are outlined in the schedule of activities (Section 2.3). Specific procedure-related details are provided above in the trial procedures (Section 6.1).

Important note: These examinations are required by the study protocol. The study protocol does not limit the examinations to those mentioned below. The investigator shall perform all other/additional routine or center-specific examinations relevant for the safety of the patient or for any other procedures done. In case of uncertainties, the coordinating investigator and/or sponsor or designee should be contacted for clarification.

6.1.5.1 Screening

<u>- 28 to - 1 days before first administration of study medication:</u>

- Obtain informed consent.
- Check inclusion/exclusion criteria.
- Review / documentation of demographics and medical history.
- Review / documentation of prior and concomitant medication.
- Review adverse events.
- Baseline tumor imaging (maximum 28 days (±3 days) prior to first study drug administration).

<u>- 14 to - 1 days prior to first study medication:</u>

- Full physical examination including height and weight.
- Vital signs (should include temperature, pulse, respiratory rate, blood pressure, oxygen saturation).
- 12-Lead electrocardiogram.
- Echocardiography
- ECOG performance status.
- WOCBP: pregnancy test – urine or serum β-HCG; if applicable: repeat maximum 24 hours before <u>first</u> study medication administration.
- Coagulation (Quick's time [PT/INR], aPTT).
- CBC with differential: white blood cell (WBC) count with differential & absolute neutrophil count (ANC); absolute lymphocyte count (ALC); red blood cells (RBCs); platelet count; hemoglobin; hematocrit.
- Comprehensive serum chemistry panel: albumin; alkaline phosphatase; alanine aminotransferase (ALT); aspartate aminotransferase (AST); lactate dehydrogenase (LDH); uric acid; calcium; glucose; phosphorus; potassium; sodium; magnesium; total bilirubin; direct bilirubin (if total bilirubin is elevated above the upper limit of normal); total protein; blood urea nitrogen; C-reactive protein (CRP); gamma-GT; creatinine, creatinine clearance, lipase.
- TSH (fT3 and fT4 to be analyzed if TSH is outside of normal range).
- Urinalysis: Blood, glucose, proteins, nitrites, ketones, leucocytes, specific gravity, pH.
- HIV, Hepatitis B and Hepatitis C screening tests

Local HER2 analysis:

- According to local standards and clinical workflow prior to enrollment, interpretation of results according to Appendix 4.

HER2 positivity is an inclusion criterion!

Archival or newly obtained tissue collection:

- Tissue biopsy according to Section 8.1.1.

Initial translational research sampling (max. -7 days before first administration of study medication):

- Blood samples: 2x 10 mL Streck® tubes
- Stool sample (520 mg ± 100 mg)
- Saliva

6.1.5.2 Treatment period

Before each study treatment (chemotherapy and/or immunotherapy)

- Review / documentation of prior and concomitant medication.
- Review adverse events.
- Directed physical examination as clinically indicated
- Vital signs (should include temperature, pulse, respiratory rate, blood pressure, oxygen saturation).
- 12-lead ECG if clinically indicated
- ECOG performance status.
- WOCBP: pregnancy test – urine or serum β-HCG; to be performed monthly and when expected menstrual cycle is missed or when pregnancy is otherwise suspected, in accordance with the CTFG guidance on contraception.
- CBC with differential: white blood cell (WBC) count with differential & absolute neutrophil count (ANC); absolute lymphocyte count (ALC); red blood cells (RBCs); platelet count; hemoglobin; hematocrit.
- Comprehensive serum chemistry panel: albumin; alkaline phosphatase; alanine aminotransferase (ALT); aspartate aminotransferase (AST); lactate dehydrogenase (LDH); uric acid; calcium; glucose; phosphorus; potassium; sodium; magnesium; total bilirubin; direct bilirubin (if total bilirubin is elevated above the upper limit of normal); total

protein; blood urea nitrogen; C-reactive protein (CRP); gamma-GT; creatinine, creatinine clearance, lipase.

- Urine analysis if clinically indicated
- Coagulation (Quick's time [PT/INR], aPTT) (if clinically indicated).
- TSH (fT3 and fT4 to be analyzed if TSH is outside of normal range).
- Translational research sampling:
    - 2x 10 mL Streck® tubes blood at d15 (before second FLOT treatment)

<u>Examinations after end of the preoperative chemo/immunotherapy and before surgery</u>

- Tumor assessment: CT or MRI scan of abdomen and thorax.
- Review / documentation of prior and concomitant medication.
- Review adverse events.
- Full physical examination including weight.
- Vital signs (should include temperature, pulse, respiratory rate, blood pressure, oxygen saturation).
- 12-Lead ECG
- ECOG performance status.
- WOCBP: pregnancy test – urine or serum β-HCG; to be performed monthly and when expected menstrual cycle is missed or when pregnancy is otherwise suspected, in accordance with the CTFG guidance on contraception.
- Coagulation (Quick's time [PT/INR], aPTT)
- Urinalysis: Blood, glucose, proteins, nitrites, ketones, leucocytes, specific gravity, pH.
- Translational research sampling:
    - 2x 10 mL Streck® tubes blood
    - Stool samples (520 mg ± 100 mg)
    - Saliva

Important note: These examinations are required by study protocol. All examinations are part of routine clinical practice. It is important to note that the study protocol does not limit the examinations to these mentioned above. The investigator shall perform all other/additional routine or center-specific examinations relevant for the safety of the patient or for any other procedures done, especially all examinations needed to ensure operability of the patient.

Examinations after surgery (4-10 weeks) / before start of post-operative treatment

- Review / documentation of prior and concomitant medication.
- Review adverse events – especially post-operative complications
- Full physical examination including weight.
- Vital signs (should include temperature, pulse, respiratory rate, blood pressure, oxygen saturation).
- ECOG performance status.
- Echocardiography
- WOCBP: pregnancy test – urine or serum β-HCG; to be performed monthly and when expected menstrual cycle is missed or when pregnancy is otherwise suspected, in accordance with the CTFG guidance on contraception.
- If applicable according to current S3 guidelines: tumor assessment by sonography or X-rays (or other standard methods) to determine the absence of the tumor/metastases.
- Translational research sampling **at surgery**
  - Paraffin embedded tissue, preferably the tumor block or alternatively up to 10 unstained slides

Day 1 of pembrolizumab plus trastuzumab cycle after post-operative FLOT chemotherapy

- Review / documentation of prior and concomitant medication.
- Review Adverse Events.
- Directed Physical Examination.
- Vital signs (should include temperature, pulse, respiratory rate and blood pressure, oxygen saturation).
- 12-Lead Electrocardiogram if clinically indicated
- Echocardiography every 3 months during trastuzumab treatment
- ECOG Performance Status.
- WOCBP: Pregnancy Test – urine or serum β-HCG; to be performed monthly and when expected menstrual cycle is missed or when pregnancy is otherwise suspected, in accordance with the CTFG guidance on contraception.
- Coagulation (Quick's time [PT/INR], aPTT) (if clinically indicated).
- CBC with differential: White Blood Cell (WBC) count with differential & Absolute Neutrophil Count (ANC); Absolute Lymphocyte Count (ALC); Red Blood Cells (RBCs); Platelet count; Hemoglobin; Hematocrit.

- Comprehensive serum chemistry panel: Albumin; Alkaline phosphatase; Alanine aminotransferase (ALT); Aspartate aminotransferase (AST); Lactate dehydrogenase (LDH); Uric Acid; Calcium; Glucose; Phosphorus; Potassium; Sodium; Magnesium; Total Bilirubin; Direct Bilirubin (If total bilirubin is elevated above the upper limit of normal); Total protein; Blood Urea Nitrogen; C-reactive protein (CRP); Gamma-GT; Creatinine, creatinine clearance, lipase.
- Urinalysis if clinically indicated
- TSH (fT3 and fT4 to be analyzed if TSH is outside of normal range).
- Translational research
  - 2x 10 mL Streck® tubes blood every 3 months after surgery until progression
  - Stool (520 mg ± 100 mg) & saliva sample after completion of adjuvant chemotherapy (= 1st cycle of pembrolizumab/trastuzumab alone) and 3 months after completion of adjuvant chemotherapy

<u>Tumor imaging:</u>

The first tumor assessment will be between the completion of the last pre-operative FLOT chemotherapy cycle/immunotherapy and surgery. Follow-up tumor assessments start 3 months after surgery and are performed every 3 months (calculated from the date of the last imaging) until 2 years have passed since EOT and every 6 months thereafter until relapse, death or end of follow-up. An earlier imaging can be performed if clinically indicated.

6.1.5.3 End of treatment visit (EOT)

- Review / documentation of prior and concomitant medication.
- Review Adverse Events.
- Full physical examination including weight.
- Vital signs (should include temperature, pulse, respiratory rate and blood pressure, oxygen saturation).
- 12-Lead electrocardiogram
- Echocardiography
- Post-study anti-cancer therapy status.
- ECOG performance status.
- WOCBP: pregnancy test – urine or serum β-HCG; to be performed monthly and when expected menstrual cycle is missed or when pregnancy is otherwise suspected, in accordance with the CTFG guidance on contraception.

- CBC with differential: white blood cell (WBC) count with differential & absolute neutrophil count (ANC); absolute lymphocyte count (ALC); red blood cells (RBCs); platelet count; hemoglobin; hematocrit.
- Comprehensive serum chemistry panel: albumin; alkaline phosphatase; alanine aminotransferase (ALT); aspartate aminotransferase (AST); lactate dehydrogenase (LDH); uric acid; calcium; glucose; phosphorus; potassium; sodium; magnesium; total bilirubin; direct bilirubin (if total bilirubin is elevated above the upper limit of normal); total protein; blood urea nitrogen; C-reactive protein (CRP); gamma-GT; creatinine, creatinine clearance, lipase.
- Urinalysis: Blood, glucose, proteins, nitrites, ketones, leucocytes, specific gravity, pH.
- TSH (fT3 and fT4 to be analyzed if TSH is outside of normal range).
- Coagulation (Quick’s time [PT/INR], aPTT).

Tumor imaging

Tumor imaging should be performed at time of discontinuation (± 4 days). For subjects who discontinue study therapy without confirmed PD per RECIST, tumor imaging should be performed at the time of treatment discontinuation (± 4 weeks). If previous tumor imaging was obtained within 4 weeks prior to date of discontinuation, then additional imaging at EOT is not required.

6.1.5.4 Post-treatment visits

6.1.5.4.1 Safety follow-up visit

The mandatory safety follow-up visit should be conducted approximately 30 days (± 7 days) after discontinuation or before the initiation of a new anti-cancer treatment, whichever comes first. All AEs that occur prior to the safety follow-up visit should be recorded. Participants with an AE of Grade > 1 will be followed until the resolution of the AE to grade 0-1 or until the beginning of a new anti-cancer therapy, whichever occurs first. SAEs that become known to the trial site within 90 days of the end of treatment or before initiation of a new anti-cancer treatment (whichever occurs first) should also be followed and recorded. The following procedures must be performed during the safety follow-up visit:

- Review / documentation of prior and concomitant medication.
- Review adverse events.
- Post-study anti-cancer therapy status.
- Vital signs (should include temperature, pulse, respiratory rate and blood pressure, oxygen saturation).
- ECOG performance status.

- WOCBP: pregnancy test – urine or serum β-HCG; to be performed monthly and when expected menstrual cycle is missed or when pregnancy is otherwise suspected until the end of relevant systemic exposure to the study drug (i.e. up to 7 months after last trastuzumab dose), in accordance with the CTFG guidance on contraception.
- CBC with differential: white blood cell (WBC) count with differential & absolute neutrophil count (ANC); absolute lymphocyte count (ALC); red blood cells (RBCs); platelet count; hemoglobin; hematocrit.
- Comprehensive serum chemistry panel: albumin; alkaline phosphatase; alanine aminotransferase (ALT); aspartate aminotransferase (AST); lactate dehydrogenase (LDH); uric acid; calcium; glucose; phosphorus; potassium; sodium; magnesium; total bilirubin; direct bilirubin (if total bilirubin is elevated above the upper limit of normal); total protein; blood urea nitrogen; C-reactive protein (CRP); gamma-GT; creatinine, creatinine clearance, lipase

6.1.5.4.2 Efficacy follow-up visits

Participants who complete the protocol-required study intervention or who discontinue study intervention for a reason other than disease progression/relapse will begin the efficacy follow-up phase and should be assessed every 3 months (± 7 days) by radiologic imaging to monitor disease status. After 2 years since EOT, imaging will be done every 6 months (± 7 days). Every effort should be made to collect information regarding disease status until the start of new anti-cancer therapy, disease progression/relapse, death or end of the study. Information regarding post-study anti-cancer treatment will be collected if new treatment is initiated. The following procedures must be performed during the follow-up visit:

- Post-Study anti-cancer therapy status.
- WOCBP: pregnancy test – urine or serum β-HCG; to be performed monthly and when expected menstrual cycle is missed or when pregnancy is otherwise suspected until the end of relevant systemic exposure to the study drug (i.e. up to 7 months after last trastuzumab dose), in accordance with the CTFG guidance on contraception.

6.1.5.4.3 Survival follow-up

Participants who experience confirmed disease progression/relapse or start a new anticancer therapy, will move into the survival follow-up phase and should be contacted within regular visits or by telephone every 3 months (± 7 days) to assess for post study anti-cancer therapy status and survival status until death, withdrawal of consent, or the end of the study, whichever occurs first.

6.2 Adverse Events (AEs), Serious Adverse Events (SAEs), and Other Reportable Safety Events

The definitions of an AE or SAE, as well as the method of recording, evaluating, and assessing causality of AE and SAE and the procedures for completing and transmitting AE, SAE, and other reportable safety event reports can be found in Appendix 3.

AEs, SAEs, and other reportable safety events will be reported by the participant (or, when appropriate, by a caregiver, surrogate, or the participant's legally authorized representative) or might be detected through abnormal laboratory values.

The investigator and any designees are responsible for detecting, documenting, and reporting events that meet the definition of an AE or SAE as well as other reportable safety events. Investigators remain responsible for following up AEs, SAEs, and other reportable safety events for outcome.

The investigator, who is a qualified physician, will assess events that meet the definition of an AE or SAE as well as other reportable safety events with respect to seriousness, intensity/toxicity and causality.

A sponsor representative (e.g., medical expert) will medically review all SAE reports and perform the expectedness assessment. Expectedness will be assessed based on the information contained in the relevant Investigator Brochures or (for FLOT) SmPCs.

As long as the trial is ongoing under the Directive 2001/20/EC (clinical trial directive, CTD) under which it was approved, the following applies:
Every SAE being assessed by either the investigator or the sponsor/sponsor representative as suspected to be related to trastuzumab, pembrolizumab or FLOT and assessed as being either unexpected or unexpected with regard to outcome or severity of the event will be reported by the sponsor/sponsor representative as SUSAR to the competent authority, responsible ethics committee and investigators of the trial in accordance with all applicable country specific regulatory requirements, global laws and regulations.

Once the trial has been transitioned to the Clinical Trials Information System (CTIS), but not later than 31 January 2025, the trial will be governed by the clinical trial Regulation (EU) No 536/2014 (CTR) and the following will apply:
Serious adverse events with at least possible relation to the trastuzumab, pembrolizumab or FLOT and assessed unexpected based on the information contained in the relevant reference safety information (RSI) (e.g., current IB and SmPC) are reported as SUSAR electronically and without delay to the European Medicines Agency via the “EudraVigilance database” (referred to in Article 40(1), Regulation (EU) No 536/2014). The period for reporting of SUSARs shall be follow according to the seriousness of the reaction:

- in the case of fatal or life-threatening SUSAR, as soon as possible and in any event not later than seven days after the Sponsor became aware of the reaction
- in the case of non-fatal or non-life-threatening SUSAR, not later than 15 days after the Sponsor became aware of the reaction
- in the case of a SUSAR which was initially considered to be non-fatal or non-life-threatening but which turns out to be fatal or life-threatening, as soon as possible and in any event not later than seven days after the Sponsor became aware of the reaction being fatal or life-threatening

In addition, the manufacturers of trastuzumab and pembrolizumab will be notified in parallel about these SUSARs.

6.2.1 Time period and frequency for collecting AE, SAE, and other reportable safety event information

All AEs, SAEs, and other reportable safety events that occur after the consent form is signed but before treatment must be reported by the investigator, if the event causes the participant to be excluded from the study or is the result of a protocol-specified intervention, including but not limited to washout or discontinuation of usual therapy, diet, or a procedure.

- All AEs from the time of treatment through 30 days following cessation of study intervention must be reported by the investigator.
- All AEs related to pembrolizumab and/or trastuzumab meeting serious criteria, from the time of treatment through 90 days following cessation of study intervention or until initiation of a new anticancer therapy must be reported by the investigator. If this new anticancer therapy starts within 30 days of cessation of study intervention, SAEs related to pembrolizumab and/or trastuzumab have to be reported for 30 days following cessation of study intervention.
- All pregnancies and exposure during breastfeeding, from the time of treatment through 7 months following cessation of study intervention, or 30 days following cessation of study intervention if the participant initiates new anticancer therapy must be reported by the investigator.
- For pharmacovigilance purposes and characterization, any SAE of MDS/AML or new primary malignancy occurring after the 30 days follow up period should be reported to the Sponsor, regardless of investigator's assessment of causality.
- Additionally, any SAE brought to the attention of an investigator at any time outside of the time period specified above must be reported immediately to the sponsor or designee if the event is considered drug-related.

Investigators are not obligated to actively seek AEs or SAEs or other reportable safety events in former study participants. However, if the investigator learns of any SAE, including a death, at any time after a participant has been discharged from the study, and he/she considers the event to be reasonably related to the study intervention or study participation, the investigator must promptly notify the sponsor or his designee who will forward the notification within 1 business day to MSD Pharmacovigilance Germany and ORGANON's Local Pharmacovigilance.

All initial and follow-up AEs, SAEs, and other reportable safety events will be recorded and reported to the sponsor or designee within the time frames as indicated in table 8. The sponsor will forward the notification within 1 business day to MSD Pharmacovigilance Germany and ORGANON's Local Pharmacovigilance.

Table 8 Reporting time periods and time frames for AEs and other reportable safety events

**Type of Event**	**Reporting time period:** **Consent to treatment start**	**Reporting time period:** **Treatment through protocol-specified follow-up period**	**Reporting time period:** **After the protocol-specified follow-up period**	**Time frame to report event and follow-up information to the sponsor or designee**
SAE including cancer and overdose	Report if:<br>- due to protocol-specified intervention<br>- causes exclusion	Report all	Report if:<br>- drug related.<br>(Follow ongoing to outcome)	Within 24 h of learning of event
Pregnancy/ lactation exposure	Report if:<br>- due to intervention<br>- causes exclusion	Report all	Previously reported – Follow to completion/termination; report outcome	Within 24 h of learning of event
ECIs<br>(require regulatory reporting)	Report if:<br>- due to intervention<br>- causes exclusion	Report<br>- potential drug-induced liver injury (DILI)<br>- require regulatory reporting	Not required	Within 24 h of learning of event

6.2.2 Method of Detecting AEs, SAEs, and other reportable safety events

Care will be taken not to introduce bias when detecting AEs and/or SAEs and other reportable safety events. Open-ended and nonleading verbal questioning of the participant is the preferred method to inquire about AE occurrence.

6.2.3 Follow-up of AE, SAE, and other reportable safety event information

After the initial AE/SAE report, the investigator is required to proactively follow each participant at subsequent visits/contacts. All AEs, SAEs, and other reportable safety events including pregnancy and exposure during breastfeeding, ECIs, cancer, and overdose will be followed until resolution, stabilization, until the event is otherwise explained, or the participant is lost to follow-up. In addition, the investigator will make every attempt to follow all nonserious AEs that occur in enrolled participants for outcome. Further information on follow-up procedures is given in Appendix 3.

6.2.4 Sponsor responsibility for reporting AEs

All AEs will be reported to regulatory authorities, IRB/IECs and investigators in accordance with all applicable country specific regulatory requirements, global laws and regulations.

6.2.5 Pregnancy and exposure during breastfeeding

Although pregnancy and infant exposure during breastfeeding are not considered AEs, any pregnancy or infant exposure during breastfeeding in a participant (spontaneously reported to the investigator or their designee) that occurs during the study are reportable to the sponsor or designee who will forward the report within 1 business day to MSD Pharmacovigilance Germany and ORGANON's Local Pharmacovigilance.

All reported pregnancies must be followed to the completion/termination of the pregnancy. Pregnancy outcomes of spontaneous abortion, missed abortion, benign hydatidiform mole, blighted ovum, fetal death, intrauterine death, miscarriage, and stillbirth must be reported as serious events (Important Medical Events). If the pregnancy continues to term, the outcome (health of infant) must also be reported.

6.2.6 Events of clinical interest (ECIs)

Selected nonserious and SAEs are also known as ECIs and must be reported to the sponsor or designee within 24 h who will forward the notification to MSD Pharmacovigilance Germany within 1 business day.

Events of clinical interest for this study include:

- An overdose of pembrolizumab that is not associated with clinical symptoms or abnormal laboratory results. For purposes of this study, an overdose will be defined as any dose of 1,000 mg or greater for pembrolizumab, i.e., ≥ 5 times the indicated dose. No specific information is available on the treatment of overdose of pembrolizumab. In the event of overdose, the participant should be observed closely for signs of toxicity. Appropriate supportive treatment should be provided if clinically indicated. If an AE is associated with

(“results from”) the overdose of pembrolizumab, the AE is reported as SAE even if no other seriousness criteria are met.

- Any event of MDS/AML, new primary malignancy, or pneumonitis should be reported whether it is considered a non-serious AE (e.g. non-melanoma skin cancer) or SAE and regardless of Investigator’s assessment of causality.
- An elevated AST or ALT lab value ≥ 3x ULN and an elevated total bilirubin lab value ≥ 2x ULN and, at the same time, an alkaline phosphatase lab value ≤ 2x ULN, as determined by way of protocol-specified laboratory testing or unscheduled laboratory testing.*

  *Note: These criteria are based upon available regulatory guidance documents. The purpose of the criteria is to specify a threshold of abnormal hepatic tests that may require an additional evaluation for an underlying etiology.

6.3 Treatment of Overdose

Pembrolizumab must only be used in accordance with the dosing recommendations in this protocol. Any dose or frequency of dosing that exceeds the dosing regimen specified in this protocol should be reported as an overdose.

No specific information is available on the treatment of overdose of pembrolizumab or trastuzumab. In the event of overdose, the study intervention should be discontinued and the participant should be observed closely for signs of toxicity. Appropriate supportive treatment should be provided if clinically indicated.

7 CONTINUOUS TOXICITY MONITORING BOARD

There will be a near real time monitoring of safety parameters (e.g., SAEs reported, any potential unexpected adverse events, accumulation of certain non-serious AEs) by a continuous toxicity monitoring board for the first 6 patients enrolled to immediately identify any risks for patient safety. In addition to these ad hoc scheduled meetings depending on (S)AE reporting, the toxicity monitoring board will meet after the first 6 patients have finished the third treatment of pembrolizumab and trastuzumab plus FLOT and have passed their presurgical assessment to re-evaluate the risk-benefit ratio of the study and provide a recommendation on the continuation of the study to the coordinating investigator and the sponsor. Recruitment can be halted at the discretion of the toxicity monitoring board.

Additional details regarding composition of the CTMB, qualification of the members, frequency and type of planned safety assessments during the study, data transmission and communication between the involved parties will be specified within a CTMB charta.

8 ACCOMPANYING RESEARCH PROJECT

The participation in translational research projects, aiming to correlate clinical efficacy with molecularly-defined subgroups, is optional for the patient and consent is necessary before any samples are provided or used within this trial.

8.1 Sampling Time Points, Materials and Analyses

Details on handling and shipping of tumor, stool, saliva and blood samples will be provided within a sample manual.
More information is listed in Appendix 5.

After performing the analyses, excess FFPE tissue material will be sent to the Sponsor for archiving.

8.1.1 Tissue Sample

A tumor tissue sample (preferably 1 FFPE block, alternatively 10 unstained slides) taken in the context of clinical routine and an additional representative sample taken during surgery should be provided for the optional translational research project.

Initial Samples must be obtained by core or excisional biopsies of a tumor lesion not previously irradiated. Samples taken during surgery should preferably contain material of the primary (if not available: representative lymph node acceptable).

This sample will be used for:

- Central analysis of PD-L1 CPS.
- Assessment of HER2
- Translational studies including antibody-based or DNA/RNA probe-based staining to analyze the spatial composition of tumor, stromal and immune cells and DNA/RNA sequencing to assess genomic alterations.
- Analyzing of the intra-tumoral microbiome by 16S rRNA sequencing

8.1.2 Blood Samples

Blood samples (2x 10 ml Streck® tubes) are taken at the following time points:

1. prior to first treatment (d0)
2. before second FLOT administration (d15)
3. before surgery (i.e. after preoperative treatment is completed)

4. after surgery (i.e. before first postoperative treatment)
5. every 3 months afterwards until relapse/progression

Blood samples must be taken before administration of study medication at each timepoint. Translational research projects might be adjusted or expanded taking the latest research data into account, but will include the following analyses:

- Genomic alterations by panel-based sequencing (DNA repair/HR panel)
- RNA sequencing
- Cytometry analysis
- Functional immune cell assays
- Tumor mutational burden
- Microsatellite instability testing.

8.1.3 Stool and Saliva Samples

Stool (520 mg +/- 100 mg) will be self-collected by the study participant either at home or at hospital with the help of a toilette accessory kit. Stool will be filled in a OMNIgene gut collection tube that keeps the microbiome stable for up to 60 days at room temperature. Saliva will be self-collected by the study participant with the help of a collection adapter.

Timepoints:

1. prior to treatment (d0, stool must be collected latest at d7 of treatment)
2. before surgery (i.e. after preoperative treatment is completed)
3. after completion of adjuvant chemotherapy (= 1st cycle of pembrolizumab/trastuzumab alone)
4. 3 months after adjuvant chemotherapy

- Oral and intestine microbiome will be analyzed by using 16S rRNA sequencing.

9 STATISTICAL ANALYSIS PLAN

9.1 Justification of sample size

The trial is designed as single arm, multicenter, open-label, phase II study, which aims to show therapeutic efficacy of the experimental regimen pembrolizumab and trastuzumab in combination with FLOT. The co-primary endpoints are the pathological complete response rate (pCR) and the disease-free survival rate after 2 years (DFSR@2).

The efficacy assumptions can be obtained from the PETRARCA and the HER-FLOT study.[28,29] In the PETRARCA study pCR rate in the standard FLOT arm was 12%. This could be increased to 22% with trastuzumab plus FLOT in the HER-FLOT trial and to 35% with trastuzumab, pertuzumab and FLOT in the PETRARCA trial. We envisage that the pCR rate for pembrolizumab, trastuzumab and FLOT could increase to 30% or more.

Hence, for the experimental regimen to be considered as a desirable candidate for further development, the pCR of 30% should be achieved, but if it is 12% or less the experimental arm would be insufficient for further development.

Formally, the hypothesis testing for the first co-primary endpoint can be defined as:

H0: P≤ 0.12 versus H1: P≥0.30

The current sample size calculation is based on pCR improvement from 12% to 30% with a one-sided alpha of 5% and a beta of 20% in a Fleming single stage phase II procedure. Thus, 27 patients are required.

Concurrently, for DFS the respective sample size calculation would be an improvement of DFS rate at 2 years from 50% (results of the FLOT 4 trial)[10] to 70% the hypothesis testing for the second co-primary endpoint can be defined as:

H0: P≤ 0.5 versus H1: P≥0.7

with a one-sided alpha of 0.1 and 80% power in a Fleming single stage phase II procedure, 27 patients are needed.

Thus, a sample size of 27 patients is sufficient for both co-primary endpoints considering that the co-primary endpoints are homogeneous, no inflation of type II error is expected. We therefore keep the statistical power at 80% for both co-primary endpoints.

Considering a drop-out rate of 10%, a total number of 30 patients will be enrolled.

9.2 Statistical analysis plan

A statistical analysis plan (SAP) will be drafted to provide details of the methods of analysis to address all study objectives. The SAP may be amended during the course of the study but will be finalized before the cut-off date for any analysis. Due to the explorative nature of this trial and the small number of patients only descriptive statistics will be performed (e.g., describing the distribution of the baseline demographic data with predefined subgroups).

9.3 Analysis

Statistical analysis is based on the International Conference on Harmonization (ICH) Guidelines “Structure and Content of Clinical Study Reports” and “Statistical Principles for Clinical Trials”. Due to the explorative nature of this trial and the small number of patients only descriptive statistics will be performed (e.g., describing the distribution of the baseline demographic data with predefined subgroups). Missing data will not be extrapolated. The number of missing values will be computed. For the time-to-event variables time to progression (TTP), PFS/DFS and OS, the Kaplan-Meier method will be used.

A detailed methodology for the statistical analysis will be described in the SAP, which will be finalized before database lock.

There is no full interim analysis planned for this study, due to the small sample size and the relatively short recruitment period. However, single objectives may be analyzed as soon as sufficient events are available for analysis as detailed in the SAP.

9.3.1 Population for analysis

All patients receiving at least one dose of study treatment will be evaluable for safety and included in the safety population.

The Full analysis set (FAS) will include all patients who received at least one treatment dose.

All efficacy analyses will be based on the FAS. Toxicity analyses will be based on the safety population

Per-protocol analysis set defined as the subset of the FAS analysis set who have no major protocol deviation including the violation of entry criteria and having the measurements of the primary variable.

9.3.1.1 Sensitivity analysis

Within the Accompanying Research Project (Section 8) PD-L1 CPS will be determined through a standardized assay (22C3 pharmDx assay, DAKO North America) by the central pathology. The primary and all meaningful secondary endpoints will additionally be analyzed with a subpopulation presenting with PD-L1 CPS ≥ 1, if different from the total population.

9.3.2 Primary endpoint

The co-primary endpoints are the disease-free survival rate after 2 years and the pathological complete response rate. We hypothesize that the DFS rate after 2 years (DFSR@2) should be 70% or more and as an interim efficacy analysis (to be read out after surgery of the last

patient) pCR rate is 30% or greater. DFSR@2 is defined as the proportion of patients being tumor/disease free and alive 2 years after enrolment. The pCR rate, defined as the absence of residual tumor based on evaluation of the resected esophagogastric specimen in the primary by local pathology

9.3.3 Secondary endpoints

- Assessment of overall response rate, defined as percentage of patients with CR and PR according to RECIST v1.1
- R0 resection rate, where R0 resection is defined as a microscopically margin negative resection with no gross or microscopic tumor remains in the areas of the primary tumor and/or sampled regional lymph nodes based on evaluation by the local pathologist.
- Overall survival (OS) where OS is defined as time from enrolment to the date of death of any cause. If no event is observed (e.g., lost to follow-up) OS is censored at the date of last subject contact. Subjects who are alive will be censored at the last known alive dates.
- Pathological complete and subtotal regression (TRG1a/b by Becker). TRG1a/b is defined as < 10% residual tumor per tumor bed based on evaluation of the resected esophagogastric specimen in the primary by local pathology.
- Perioperative morbidity and mortality
- Safety and toxicity: Adverse events will be recorded and graded according to version 5.0 of National Cancer Institute Common Toxicity Criteria (NCI-CTC). Occurrence of any adverse event and occurrence of any serious adverse event (anytime during the study) will be presented. These events will also be described by nature (Primary System Organ Class and Preferred Term), severity and causal relationship to drug administration.

9.3.4 Exploratory endpoints

Assessment whether clinical efficacy correlates with molecularly-defined subgroups (PD-L1 expression, MSI subtypes, and others).

10 LABELING, PACKAGING, STORAGE AND RETURN OF CLINICAL SUPPLIES

10.1 Investigational product

The investigator shall take responsibility for and shall take all steps to maintain appropriate records and ensure appropriate supply, storage, handling, distribution and usage of investigational product in accordance with the protocol and any applicable laws and regulations.

Pembrolizumab will be provided by MSD as summarized in table 9.

Trastuzumab (Ontruzant®) will be provided by ORGANON as unlabeled, unchanged commercial product as summarized in table 9.

Table 9 Product descriptions

Product Name & Potency	Dosage Form
KEYTRUDA® (Pembrolizumab)<br>100 mg/ 4 mL (= 25 mg/mL)	Solution for Injection
ONTRUZANT® (Trastuzumab)<br>150 mg (reconstituted solution = 21 mg/mL)<br>420 mg (reconstituted solution = 21 mg/mL)	Powder for a concentrate for the preparation of a solution for infusion

All other supplies not indicated above will be provided locally by the trial site, subsidiary or designee, especially – but not limited to – relating to the FLOT therapy regimen.

10.2 Packaging and labeling information

Supplies will be labeled in accordance with regulatory requirements.

10.3 Clinical supplies disclosure

This trial is open-label; therefore, the participant, the trial site personnel, the sponsor and/or designee are not blinded to treatment. Drug identity (name, strength) is included in the label text; random code/disclosure envelopes or lists are not provided.

10.4 Storage and handling requirements

Clinical supplies must be stored in a secure, limited-access location under the storage conditions specified on the label.

Receipt and dispensing of trial medication must be recorded by an authorized person at the trial site.

Clinical supplies may not be used for any purpose other than that stated in the protocol.

10.5 Returns and reconciliation

The investigator is responsible for keeping accurate records of the clinical supplies received by MSD, ORGANON or the sponsor or any designee, the amount dispensed to and returned by the participants and the amount remaining at the conclusion of the trial.

Upon completion or termination of the study, all unused and/or partially used investigational product will be destroyed at the site per institutional policy. It is the investigator's responsibility to arrange for disposal of all empty containers, provided that procedures for proper disposal

have been established according to applicable federal, state, local and institutional guidelines and procedures, and provided that appropriate records of disposal are kept.

11 ADMINISTRATIVE AND REGULATORY DETAILS

11.1 Regulatory and ethical compliance

This clinical study was designed and shall be implemented and reported in accordance with the protocol, the AMG (Arzneimittelgesetz), the ICH Harmonized Tripartite Guidelines for Good Clinical Practice (GCP), with applicable local regulations (including European Directive 2001/20/EC or Regulation (EU) No 536/2014), and with the ethical principles laid down in the Declaration of Helsinki.

Before recruitment into the clinical trial, each patient will be informed that participation in the study is completely voluntary, and that he or she may withdraw his or her participation in the trial at any time without any declaration of reasons. This will not lead to any disadvantage for the respective patient. If the withdrawal is caused by an adverse drug event, the patient should inform the Investigator about this fact.

11.2 Registration and request for authorization of the trial

According to GCP-V, the trial has to be submitted to and to be authorized/approved by the competent authority (Paul-Ehrlich-Institut, PEI) and the ethics committee(s) responsible for the trial. The respective local authorities will be informed about the trial and the participation of individual trial sites.

The competent authority and the competent ethics committee will be informed on the course of the study with respect to safety aspects to be announced as well as on the termination of the trial and the trial results according to GCP-V. Database closure is defined as the end of the trial, because sites need to collect survival data of patients and are involved in the data cleaning process actively (e.g. additional source data may be requested, and additional monitoring visits may be necessary).

By no later than January 31, 2025, the present trial will be transitioned to Clinical Trial Information System (CTIS) and will be subject to Regulation (EU) No. 536/2014 from the date of transition.

11.3 Ethics committee

Prior to start of the trial the study protocol and all additionally relevant documents will be sent by the sponsor or designee to the competent ethics committee in order to receive the committee's opinion. The trial is only allowed to start after a positive vote of the ethics

committee has been received. During the course of the study the sponsor or designee will inform the ethics committee about all amendments to the study protocol as well as on all SUSARs emerging from the trial according to GCP-V in writing as long as the trial is governed by Directive 2001/20/EC. After transition to CTIS and when the trial is governed by Regulation (EU) No 536/2014, the respective competent authorities/ ethics committee will be informed through the “EU portal” (part of CTIS). In addition, the competent ethical committee will receive a development safety update report once a year.

All subsequent protocol amendments and amendments to the informed consent form will be submitted to the competent ethics committee to obtain an updated vote before implementation of the changes. Serious or unexpected adverse events occurring during the trial likely to affect the safety of the subjects or the conduct of the trial will also be reported to the ethics committee.

In addition, the competent ethics committee will be informed on the course of the study with respect to the termination of the trial and the trial results in writing as long as the trial is governed by Directive 2001/20/EC. Once the trial is governed by Regulation (EU) No 536/2014 the Sponsor or its delegate will notify the respective competent authorities of the Member State concerned of the end of the clinical trial through the “EU portal” within 15 days.

11.4 Informed consent

It is the investigator’s responsibility to obtain written informed consent from the patient after adequate explanation of the aims, methods, anticipated benefits, and potential hazards of the study and before any study specific procedures are performed. The patient should be given a copy of the informed consent documentation. The wet ink copy of the signed and dated informed consent must be retained in the institution’s records and is subject to inspection by representatives of the Sponsor or representatives from regulatory agencies.

11.5 Insurance

A clinical trials insurance is contracted in accordance with the local law before submission of the study to the relevant authorities. A copy of the confirmation and the conditions of the insurance will be handed out to every study participant together with the informed consent form.

11.6 Confidentiality

By signing this protocol, the investigator affirms to the sponsor that information furnished to the investigator by the sponsor will be maintained in confidence, and such information will be divulged to the local ethics committee or similar or expert committee, affiliated institution and employees, only under an appropriate understanding of confidentiality with such board or

committee, affiliated institution and employees. Data generated by this trial will be considered confidential by the Investigator, except to the extent that it is included in a publication as provided in Section 11.11.2 of this protocol.

11.7 Confidentiality of subject records

By signing this protocol, the Investigator agrees that the sponsor (or designee), ethics committee, or regulatory authority representatives may consult and/or copy trial documents in order to verify worksheet/case report form data. By signing the consent form, the subject agrees to this process. If trial documents will be photocopied during the process of verifying worksheet/case report form information, the subject will be identified by the screening or trial subject number only; full names/initials will be masked prior to transmission to the sponsor or designee.

By signing this protocol, the investigator agrees to treat all subject data used and disclosed in connection with this trial in accordance with all applicable privacy laws, rules and regulations.

11.8 Confidentiality of investigator information

By signing this protocol, the investigator recognizes that certain personal identifying information with respect to the investigator, and all sub-investigators and trial site personnel, may be used and disclosed for trial management purposes, as part of regulatory submissions, and as required by law. This information may include:

- Name, address, telephone number and e-mail address
- Hospital or clinic address and telephone number
- *Curriculum vitae* or other summary of qualifications and credentials

  and
- Other professional documentation.

Additionally, the investigator’s name and business contact information may be included when reporting certain serious adverse events to regulatory authorities, to other Investigators, or to MSD and ORGANON. By signing this protocol, the investigator expressly consents to these uses and disclosures.

As this is a multicenter trial, in order to facilitate contact between investigators, the sponsor or designee may share an investigator’s name and contact information with other participating investigators upon request.

11.9 Compliance with financial disclosure requirements

The investigator/sub-investigator(s) agree to provide his/her financial interests in and/or arrangements with the sponsor on a financial disclosure form provided by the sponsor or designee.

11.10 Quality management system

11.10.1 Quality control and quality assurance

The standard operating procedures (SOPs) of the sponsor or designee or its designated sub-contractors (if applicable) are used for conduction of the trial.

11.10.2 Audits and inspections

In case of an audit by the sponsor or designee or an appropriate authority, the investigator will make all relevant documents available. If an audit visit by a regional authority is announced, the respective trial site should inform the sponsor or designee as early as possible in order to allow for an appropriate preparation and support.

11.10.3 Monitoring

It is understood that outside monitor and other authorized personnel may contact and visit the investigator, and that they will be allowed direct access to source data/documents for trial-related monitoring, audits, ethic committee review, and regulatory inspection. Direct access is defined as permission to examine, analyze, verify, and reproduce any records and reports that are important to evaluation of a clinical trial. All reasonable precautions within the constraints of the applicable regulatory requirement(s) to maintain the confidentiality of subjects' identities and Sponsor's proprietary information will be exercised (Guideline for Good Clinical Practice, ICH Harmonized Tripartite Guideline, adopted July 1996: Chapter 5.15.1 and 1.21, respectively).

It is the monitor's responsibility to inspect the case report forms at regular intervals throughout the trial to verify adherence to the protocol: the completeness, accuracy, and consistency of the data; and adherence to GCP guidelines. The monitor should have access to patient charts, laboratory reports, and other patient records needed to verify the entries on the case report forms. Where local rules do not allow direct access to the source data, the monitor will verify entries in the case report form by asking direct questions of a person or persons with authorized access to the source data. The Investigator agrees to cooperate with the monitor to ensure that any problems detected during the course of these monitoring visits are resolved.

A monitoring plan with relevant details will be issued and regularly updated throughout the trial.

11.11 Notification of Serious Breaches

The Sponsor or its delegate will notify the Member States concerned about a serious breach through the "EU portal" without undue delay but not later than seven days of becoming aware of that breach. A serious breach is defined as any breach of:

- the Regulation (EU) No 536/2014

  or

- the clinical trial protocol version applicable at the time of the breach

which is likely to affect to significant degree:

- the safety of the trial participants
- the rights of the trial participants
- the reliability and robustness of the data generated in that trial.

Furthermore, all cases of potential fraud will be reported as serious breach. Further information and examples of situations that may be considered as serious breaches can be found in the "Guideline for the notification of serious breaches of Regulation (EU) No 536/2014 or the clinical trial protocol" (https://www.ema.europa.eu/en/documents/scientific-guideline/guideline-notification-serious-breaches-regulation-eu-no-536/2014-clinical-trial-protocol_en.pdf)

Suspected serious breaches should be promptly reported to the Sponsor by the trial sites in order to perform further investigations. The Sponsor will perform a thorough root cause analysis to identify the cause of the serious breach and to assess the impact of the breach on the reliability and robustness of the trial data as well as the impact on the participant's safety and/or rights. The assessment will be documented to allow review of the appropriateness of the decisions and actions taken by the Sponsor as part of the process triggered by the notification of a serious breach.

The management of a serious breach will be performed according to the corresponding SOP of the Sponsor.

11.12 Data management

An electronic data capture system will be used in this trial. Data collection and capture, creation of queries, and data analysis will be performed by the sponsor or designee or its designated sub-contractors (if applicable). GCP-compliant handling of the data is secured by

adequate SOPs. Archiving of data and results electronically recorded will be at least 10 years after the end of this study as long as this trial is governed by Directive 2001/20/EC and at least 25 years after the end of trial once this trial is governed by Regulation (EU) No 536/2014.

11.12.1 Data Handling/Data Capture System

Designated investigator staff will enter the data required by the protocol into the electronic case report forms (eCRF). During data entry automatic validation check will take place to determine data discrepancies and, by generating appropriate error messages, allow these data to be confirmed or corrected. All required data entry fields must be completed. Data corrections will be done according to the instructions provided. The Investigator will be asked to confirm the accuracy of completed CRFs by electronical signature.

Source documents pertaining to the trial must be maintained by investigational sites. Source documents may include a participant's medical records, hospital charts, clinic charts, the Investigator's study files, as well as the results of diagnostic tests such as X-rays, laboratory tests, and electrocardiograms. The Investigator's copy of the electronic case report forms serves as part of the Investigator's record of a participant's study-related data. Data that are derived from source documents and reported on the eCRF must be consistent or the discrepancies must be explained. Subsequent to the final database lock, validated data will be transferred to the sponsor.

11.12.2 Plausibility check, data cleaning and coding

Qualified members of the sponsor or designee or its designated sub-contractors (if applicable) regularly perform plausibility checks and data cleaning according to the data-cleaning plan. Some of the raw data of the eCRF also needs coding (e.g., surgical procedures, toxicities) according to the data-coding plan. Before data base closure, data cleaning and manual plausibility checks have to be performed for the relevant contents (defined by the data-cleaning plan), and all open questions have to be resolved.

11.12.3 Publication and registration of the study

The results of this study will be published by the coordinating investigator and/or sponsor after final analysis has been performed. Publication will be independent of the nature of the results obtained (whether they were positive or negative). The manuscript written for publication, together with the materials provided by the statistician can be accepted as the final study report. Once the trial is governed by the Regulation (EU) No 536/2014, at the latest one year after completion of the entire trial, summary results will be made available in the European database (www.clinicaltrialsregister.eu) - also in layman understandable form.

This clinical trial will be registered at a primary register of the WHO, e.g., at www.clinicaltrials.gov.

12 REFERENCES

1 Tabernero, J *et al.* 2019. Pembrolizumab with or without chemotherapy versus chemotherapy for advanced gastric or gastroesophageal junction (G/GEJ) adenocarcinoma: The phase III KEYNOTE-062 study. *Journal of Clinical Oncology* **37:**LBA4007

2 Arnold, M, Ferlay, J, van Berge Henegouwen, MI & Soerjomataram, I. 2020. Global burden of oesophageal and gastric cancer by histology and subsite in 2018. *Gut* **69:**1564-71

3 Janjigian, YY *et al.* 2021. First-line nivolumab plus chemotherapy versus chemotherapy alone for advanced gastric, gastro-oesophageal junction, and oesophageal adenocarcinoma (CheckMate 649): a randomised, open-label, phase 3 trial. *Lancet* **398:**27-40

4 . Sun *et al.* 2021. Pembrolizumab plus chemotherapy versus chemotherapy alone for first-line treatment of advanced oesophageal cancer (KEYNOTE-590): a randomised, placebo-controlled, phase 3 study. *Lancet* **398** (10302):759-771

5 Al-Batran, SE *et al.* 2017. Effect of Neoadjuvant Chemotherapy Followed by Surgical Resection on Survival in Patients With Limited Metastatic Gastric or Gastroesophageal Junction Cancer: The AIO-FLOT3 Trial. *JAMA oncology* **3**:1237-44

6 Al-Batran, SE *et al.* 2008. Phase III trial in metastatic gastroesophageal adenocarcinoma with fluorouracil, leucovorin plus either oxaliplatin or cisplatin: a study of the Arbeitsgemeinschaft Internistische Onkologie. *J Clin Oncol* **26:**1435-42

7 Al-Batran, SE *et al.* 2016. Histopathological regression after neoadjuvant docetaxel, oxaliplatin, fluorouracil, and leucovorin versus epirubicin, cisplatin, and fluorouracil or capecitabine in patients with resectable gastric or gastro-oesophageal junction adenocarcinoma (FLOT4-AIO): results from the phase 2 part of a multicentre, open-label, randomised phase 2/3 trial. *Lancet Oncol* **17:**1697-708

8 Al-Batran, SE *et al.* 2017. Effect of Neoadjuvant Chemotherapy Followed by Surgical Resection on Survival in Patients With Limited Metastatic Gastric or Gastroesophageal Junction Cancer: The AIO-FLOT3 Trial. *JAMA Oncol* **3:**1237-44

9 Al-Batran, SE *et al.* 2013. The feasibility of triple-drug chemotherapy combination in older adult patients with oesophagogastric cancer: a randomised trial of the Arbeitsgemeinschaft Internistische Onkologie (FLOT65+). *Eur J Cancer* **49:**835-42

10 Al-Batran, SE *et al.* 2019. Perioperative chemotherapy with fluorouracil plus leucovorin, oxaliplatin, and docetaxel versus fluorouracil or capecitabine plus cisplatin and epirubicin for locally advanced, resectable gastric or gastro-oesophageal junction adenocarcinoma (FLOT4): a randomised, phase 2/3 trial. *Lancet* **393:**1948-57

11 Disis, ML. 2010. Immune regulation of cancer. *J Clin Oncol* **28:**4531-8

12 Dudley, ME *et al.* 2005. Adoptive cell transfer therapy following non-myeloablative but lymphodepleting chemotherapy for the treatment of patients with refractory metastatic melanoma. *J Clin Oncol* **23:**2346-57

13 Hunder, NN *et al.* 2008. Treatment of metastatic melanoma with autologous CD4+ T cells against NY-ESO-1. *N Engl J Med* **358:**2698-703

14 Greenwald, RJ, Freeman, GJ & Sharpe, AH. 2005. The B7 family revisited. *Annu Rev Immunol* **23:**515-48

15 Okazaki, T, Maeda, A, Nishimura, H, Kurosaki, T & Honjo, T. 2001. PD-1 immunoreceptor inhibits B cell receptor-mediated signaling by recruiting src homology 2-domain-containing tyrosine phosphatase 2 to phosphotyrosine. *Proc Natl Acad Sci U S A* **98:**13866-71

16 Chemnitz, JM, Parry, RV, Nichols, KE, June, CH & Riley, JL. 2004. SHP-1 and SHP-2 associate with immunoreceptor tyrosine-based switch motif of programmed death 1 upon primary human T cell stimulation, but only receptor ligation prevents T cell activation. *J Immunol* **173:**945-54

17 Riley, JL. 2009. PD-1 signaling in primary T cells. *Immunol Rev* **229:**114-25

18 Sheppard, KA *et al.* 2004. PD-1 inhibits T-cell receptor induced phosphorylation of the ZAP70/CD3zeta signalosome and downstream signaling to PKCtheta. *FEBS Lett* **574:**37-41
19 Francisco, LM, Sage, PT & Sharpe, AH. 2010. The PD-1 pathway in tolerance and autoimmunity. *Immunol Rev* **236:**219-42
20 Shitara, K *et al.* 2020. Efficacy and Safety of Pembrolizumab or Pembrolizumab Plus Chemotherapy vs Chemotherapy Alone for Patients With First-line, Advanced Gastric Cancer: The KEYNOTE-062 Phase 3 Randomized Clinical Trial. *JAMA Oncol* **6:**1571-80
21 Akiyama, T, Sudo, C, Ogawara, H, Toyoshima, K & Yamamoto, T. 1986. The product of the human c-erbB-2 gene: a 185-kilodalton glycoprotein with tyrosine kinase activity. *Science* **232:**1644-6
22 Holbro, T *et al.* 2003. The ErbB2/ErbB3 heterodimer functions as an oncogenic unit: ErbB2 requires ErbB3 to drive breast tumor cell proliferation. *Proc Natl Acad Sci U S A* **100:**8933-8
23 Boku, N. 2014. HER2-positive gastric cancer. *Gastric Cancer* **17:**1-12
24 Krishnamurti, U & Silverman, JF. 2014. HER2 in breast cancer: a review and update. *Adv Anat Pathol* **21:**100-7
25 Klapper, LN, Waterman, H, Sela, M & Yarden, Y. 2000. Tumor-inhibitory antibodies to HER-2/ErbB-2 may act by recruiting c-Cbl and enhancing ubiquitination of HER-2. *Cancer Res* **60:**3384-8
26 Wilson, FR *et al.* 2018. Herceptin(R) (trastuzumab) in HER2-positive early breast cancer: a systematic review and cumulative network meta-analysis. *Syst Rev* **7:**191
27 Bang, YJ *et al.* 2010. Trastuzumab in combination with chemotherapy versus chemotherapy alone for treatment of HER2-positive advanced gastric or gastro-oesophageal junction cancer (ToGA): a phase 3, open-label, randomised controlled trial. *Lancet* **376:**687-97
28 Hofheinz, RD *et al.* 2021. Trastuzumab in combination with 5-fluorouracil, leucovorin, oxaliplatin and docetaxel as perioperative treatment for patients with human epidermal growth factor receptor 2-positive locally advanced esophagogastric adenocarcinoma: A phase II trial of the Arbeitsgemeinschaft Internistische Onkologie Gastric Cancer Study Group. *International journal of cancer* **149:**1322-31
29 Hofheinz, RD *et al.* 2020. Perioperative trastuzumab and pertuzumab in combination with FLOT versus FLOT alone for HER2-positive resectable esophagogastric adenocarcinoma: Final results of the PETRARCA multicenter randomized phase II trial of the AIO. *Journal of Clinical Oncology* **38:**4502-
30 Gennari, R *et al.* 2004. Pilot study of the mechanism of action of preoperative trastuzumab in patients with primary operable breast tumors overexpressing HER2. *Clin Cancer Res* **10:**5650-5
31 Muller, P *et al.* 2015. Trastuzumab emtansine (T-DM1) renders HER2+ breast cancer highly susceptible to CTLA-4/PD-1 blockade. *Sci Transl Med* **7:**315ra188
32 Janjigian, YY *et al.* 2020. First-line pembrolizumab and trastuzumab in HER2-positive oesophageal, gastric, or gastro-oesophageal junction cancer: an open-label, single-arm, phase 2 trial. *Lancet Oncol* **21:**821-31
33 Rha, SY *et al.* 2021. A multi-institutional phase Ib/II trial of first-line triplet regimen (Pembrolizumab, Trastuzumab, Chemotherapy) for HER2-positive advanced gastric and gastroesophageal junction cancer (PANTHERA Trial): Molecular profiling and clinical update. *Journal of Clinical Oncology* **39:**218-
34 Janjigian, YY *et al.* 2021. Pembrolizumab plus trastuzumab and chemotherapy for HER2+ metastatic gastric or gastroesophageal junction (G/GEJ) cancer: Initial findings of the global phase 3 KEYNOTE-811 study. *Journal of Clinical Oncology* **39:**4013-
35 Bang, YJ *et al.* 2019. KEYNOTE-585: Phase III study of perioperative chemotherapy with or without pembrolizumab for gastric cancer. *Future Oncol* **15:**943-52
36 Collisson, EA *et al.* 2014. Comprehensive molecular profiling of lung adenocarcinoma. *Nature* **511:**543-50

37 Le, DT *et al.* 2015. PD-1 Blockade in Tumors with Mismatch-Repair Deficiency. *N Engl J Med* **372:**2509-20
38 Pietrantonio, F *et al.* 2016. HER2 loss in HER2-positive gastric or gastroesophageal cancer after trastuzumab therapy: Implication for further clinical research. *International journal of cancer* **139:**2859-64
39 Roberti, MP *et al.* 2020. Chemotherapy-induced ileal crypt apoptosis and the ileal microbiome shape immunosurveillance and prognosis of proximal colon cancer. *Nat Med* **26:**919-31
40 Routy, B *et al.* 2018. Gut microbiome influences efficacy of PD-1-based immunotherapy against epithelial tumors. *Science* **359:**91-7
41 Yu, T *et al.* 2017. Fusobacterium nucleatum Promotes Chemoresistance to Colorectal Cancer by Modulating Autophagy. *Cell* **170:**548-63 e16
42 Siewert, JR, Holscher, AH, Becker, K & Gossner, W. 1987. [Cardia cancer: attempt at a therapeutically relevant classification]. *Chirurg* **58:**25-32
43 Oken, MM *et al.* 1982. Toxicity and response criteria of the Eastern Cooperative Oncology Group. *Am J Clin Oncol* **5:**649-55
44 Stein, A., *et al.* 2022. Efficacy of Ipilimumab vs FOLFOX in Combination With Nivolumab and Trastuzumab in Patients With Previously Untreated ERBB2-Positive Esophagogastric Adenocarcinoma: The AIO INTEGA Randomized Clinical Trial. *JAMA Oncol.* **8(8)**:1150-1158.
45 Moehler M *et al.* 2021. Phase III Trial of Avelumab Maintenance After First-Line Induction Chemotherapy Versus Continuation of Chemotherapy in Patients With Gastric Cancers: Results From JAVELIN Gastric 100. *J Clin Oncol* **39(9)**:966-977
46 7 Xie T *et al.* 2021. Appropriate PD-L1 Cutoff Value for Gastric Cancer Immunotherapy: A Systematic Review and Meta-Analysis. *Front Oncol* **11**:646355
47 Janjigian, YY *et al.* 2022. MATTERHORN: phase III study of durvalumab plus FLOT chemotherapy in resectable gastric/gastroesophageal junction cancer. *Future Oncol* 2022 Jun;**18(20)**:2465-2473

13 APPENDICES

Appendix 1: ECOG Performance Status

Table 10 Appendix 1 - ECOG Performance Status [43]

Grade	Description
0	Normal activity. Fully active, able to carry on all pre-disease performance without restriction.
1	Symptoms, but ambulatory. Restricted in physically strenuous activity, but ambulatory and able to carry out work of a light or sedentary nature (e.g., light housework, office work).
2	In bed < 50% of the time. Ambulatory and capable of all self-care, but unable to carry out any work activities. Up and about more than 50% of waking hours.
3	In bed > 50% of the time. Capable of only limited self-care, confined to bed or chair more than 50% of waking hours.
4	100% bedridden. Completely disabled. Cannot carry on any self-care. Totally confined to bed or chair.
5	Dead.

Appendix 2: Contraceptive guidance and pregnancy testing

Woman of Childbearing Potential (WOCBP):

A woman is considered fertile following menarche and until becoming post-menopausal unless permanently sterile (see below).

Women in the following categories are not considered WOCBP:

- Premenarchal
- Premenopausal female with 1 of the following:
  - Documented hysterectomy
  - Documented bilateral salpingectomy
  - Documented bilateral oophorectomy

  Note: Documentation can come from the site personnel's review of the participant's medical records, medical examination, or medical history interview.
- Postmenopausal female
  - A postmenopausal state is defined as no menses for 12 months without an alternative medical cause.
    - A high follicle stimulating hormone (FSH) level in the postmenopausal range may be used to confirm a postmenopausal state in women not using hormonal contraception or hormonal replacement therapy (HRT). However, in the absence of 12 months of amenorrhea, confirmation with two FSH measurements in the postmenopausal range is required.
  - Females on HRT and whose menopausal status is in doubt will be required to use one of the non-hormonal highly effective contraception methods if they wish to continue their HRT during the study. Otherwise, they must discontinue HRT to allow confirmation of postmenopausal status before study enrollment.

<u>Contraception requirements</u>

Male participants:

Male participants with female partners of childbearing potential are eligible to participate if they agree to one of the following during the protocol defined time frame:

- Be abstinent from penile-vaginal intercourse as their usual and preferred lifestyle (abstinent on a long term and persistent basis) and agree to remain abstinent
- Use a male condom plus partner use of a contraceptive method with a failure rate of < 1% per year as described in Table Appendix 2-1 when having penile-vaginal intercourse with a WOCBP who is not currently pregnant.
  - Note: Men with a pregnant or breastfeeding partner must agree to remain abstinent from penile-vaginal intercourse or use a male condom during each episode of penile penetration.

Female participants:

Female participants of childbearing potential are eligible to participate if they agree to use a highly effective method of contraception consistently and correctly as described in Table 11 from the day of study intervention initiation (or 14 days prior to the initiation of study intervention for oral contraception) throughout the trial period up to 7 months after the last dose of study intervention.

Table 11 Appendix 2 - Highly effective contraception methods

**Highly effective methods that have low user dependency**<br>*Failure rate of <1% per year when used consistently and correctly.*
• Progestogen- only contraceptive implant [a,b]<br>• Intrauterine hormone-releasing system (IUS) [a]<br>• Intrauterine device (IUD)<br>• Bilateral tubal occlusion
● **Vasectomized partner**<br>A vasectomized partner is a highly effective contraception method provided that the partner is the sole male sexual partner of the WOCBP and the absence of sperm has been confirmed. If not, an additional highly effective method of contraception should be used.
● **Sexual abstinence**<br>Sexual abstinence is considered a highly effective method only if defined as refraining from heterosexual intercourse during the entire period of risk associated with the study treatment. The reliability of sexual abstinence needs to be evaluated in relation to the duration of the study and the preferred and usual lifestyle of the participant.
Notes: Use should be consistent with local regulations regarding the use of contraceptive methods for participants of clinical studies.

a) If locally required, in accordance with Clinical Trial Facilitation Group (CTFG) guidelines, acceptable contraceptive implants are limited to those which inhibit ovulation.
b) If hormonal contraception efficacy is potentially decreased due to interaction with study treatment, condoms must be used in addition to the hormonal contraception during the treatment period and for at least 7 months after the last trastuzumab dose.

Pregnancy Testing

WOCBP should only be included after a negative highly sensitive urine or serum pregnancy test. If applicable, this test should be repeated a maximum of 24 hours before the first dose. Following initiation of treatment, pregnancy testing will be performed at monthly intervals during the treatment period and until 7 months after the last dose of study treatment and as required locally. Pregnancy testing will be performed whenever an expected menstrual cycle is missing or when pregnancy is otherwise suspected.

Important Note: Women should not breast-feed during trastuzumab therapy and for 7 months after the last dose.

Appendix 3: Adverse Events: Definitions and procedures for recording, evaluating, follow-up, and reporting

The descriptions and grading scales found in the revised NCI Common Terminology Criteria for Adverse Events (CTCAE) version 5.0 will be utilized for AE reporting. (http://ctep.cancer.gov/reporting/ctc.html).

Definition of AE

- An AE is any untoward medical occurrence in a clinical study participant, temporally associated with the use of study intervention, whether or not considered related to the study intervention.
- NOTE: An AE can therefore be any unfavorable and unintended sign (including an abnormal laboratory finding), symptom, or disease (new or exacerbated) temporally associated with the use of a study intervention.
- NOTE: For purposes of AE definition, study intervention includes any pharmaceutical product, biological product, vaccine, diagnostic agent, or protocol specified procedure whether investigational or marketed (including active comparator product, or run-in intervention), manufactured by, licensed by, provided by, or distributed by MSD or ORGANON for human use in this study.

Events meeting the AE definition

- Any abnormal laboratory test results (hematology, clinical chemistry, or urinalysis) or other safety assessments (e.g., ECG, radiological scans, vital signs measurements), including those that worsen from baseline, considered clinically significant in the medical and scientific judgment of the investigator.
- Exacerbation of a chronic or intermittent pre-existing condition including either an increase in frequency and/or intensity of the condition.
- New conditions detected or diagnosed after study intervention administration even though it may have been present before the start of the study.
- Signs, symptoms, or the clinical sequelae of a suspected drug-drug interaction.
- Signs, symptoms, or the clinical sequelae of a suspected overdose of either study intervention or a concomitant medication.
- For all reports of overdose (whether accidental or intentional) with an associated AE, the AE term should reflect the clinical symptoms or abnormal test result. An overdose without any associated clinical symptoms or abnormal laboratory results is reported using the terminology “accidental or intentional overdose without adverse effect.”

Events NOT meeting the AE definition

- Medical or surgical procedure (e.g., endoscopy, appendectomy): the condition that leads to the procedure is the AE.
- Situations in which an untoward medical occurrence did not occur (social and/or convenience admission to a hospital).
- Anticipated day-to-day fluctuations of pre-existing disease(s) or condition(s) present or detected at the start of the study that do not worsen.
- Surgery planned prior to informed consent to treat a pre-existing condition that has not worsened.

Definition of SAE

If an event is not an AE per definition above, then it cannot be an SAE even if serious conditions are met.

An SAE is defined as any untoward medical occurrence that, at any dose:

- **Results in death**
- **Is life-threatening**
  - The term “life-threatening” in the definition of “serious” refers to an event in which the participant was at risk of death at the time of the event. It does not refer to an event, which hypothetically might have caused death, if it were more severe.
- **Requires inpatient hospitalization or prolongation of existing hospitalization**
  - Hospitalization is defined as an inpatient admission, regardless of length of stay, even if the hospitalization is a precautionary measure for continued observation. (Note: Hospitalization for an elective procedure to treat a pre-existing condition that has not worsened is not an SAE. A pre-existing condition is a clinical condition that is diagnosed prior to the use of an MSD product or other investigational agent and is documented in the participant’s medical history.
- **Results in persistent or significant disability/incapacity**
  - The term disability means a substantial disruption of a person’s ability to conduct normal life functions.
  - This definition is not intended to include experiences of relatively minor medical significance such as uncomplicated headache, nausea, vomiting, diarrhea, influenza, and accidental trauma (e.g., sprained ankle) that may interfere with or prevent everyday life functions but do not constitute a substantial disruption.
- **Is a congenital anomaly/birth defect**
  - In offspring of participant taking the product regardless of time to diagnosis.

- **Other important medical events**
  - Medical or scientific judgment should be exercised in deciding whether SAE reporting is appropriate in other situations such as important medical events that may not be immediately life-threatening or result in death or hospitalization but may jeopardize the participant or may require medical or surgical intervention to prevent 1 of the other outcomes listed in the above definition. These events should usually be considered serious.
  - Examples of such events include invasive or malignant cancers, intensive treatment in an emergency room or at home for allergic bronchospasm, blood dyscrasias or convulsions that do not result in hospitalization, or development of drug dependency or drug abuse.

Additional events reported in the same manner as SAE

In addition to the above criteria, AEs meeting either of the below criteria, although not serious per ICH definition, are reportable to the sponsor or designee in the same time frame as SAEs to meet certain local requirements. Therefore, these events are considered serious by the sponsor for collection purposes.

- Is a new cancer (that is not a condition of the study)
- Is associated with an overdose of pembrolizumab and/or trastuzumab

Recording AE and SAE

AE and SAE recording

- When an AE/SAE occurs, it is the responsibility of the investigator to review all documentation (e.g., hospital progress notes, laboratory, and diagnostics reports) related to the event.
- The investigator will record all relevant AE/SAE information on the AE eCRFs/ worksheets at each examination.
- There may be instances when copies of medical records for certain cases are requested by MSD Pharmacovigilance Germany or ORGANON's Local Pharmacovigilance. In this case, all participant identifiers, with the exception of the participant number, will be blinded on the copies of the medical records, sent to the Sponsor or designee before forwarding to MSD Pharmacovigilance Germany and ORGANON's Local Pharmacovigilance.
- The investigator will attempt to establish a diagnosis of the event based on signs, symptoms, and/or other clinical information. In such cases, the diagnosis (not the individual signs/ symptoms) will be documented as the AE/SAE.

Assessment of intensity/toxicity

An event is defined as “serious” when it meets at least 1 of the predefined outcomes as described in the definition of an SAE, not when it is rated as severe.

The investigator will assess the of intensity for each AE and SAE (and other reportable safety event) according to the NCI Common Terminology for Adverse Events (CTCAE), version 5. Any AE that changes CTCAE grade over the course of a given episode will have each change of grade recorded on the AE eCRFs/worksheets.

- Grade 1: Mild; asymptomatic or mild symptoms; clinical or diagnostic observations only; intervention not indicated.
- Grade 2: Moderate; minimal, local or noninvasive intervention indicated; limiting age-appropriate instrumental activities of daily living (ADL).
- Grade 3: Severe or medically significant but not immediately life-threatening; hospitalization or prolongation of hospitalization indicated; disabling; limiting self-care ADL.
- Grade 4: Life threatening consequences; urgent intervention indicated.
- Grade 5: Death related to AE.

Assessment of causality

1. Did the investigational agent cause the AE?
2. The determination of the likelihood that the investigational agent caused the AE will be provided by an investigator who is a qualified physician. The investigator’s signed/ dated initials on the source document or worksheet that supports the causality noted on the AE form, ensures that a medically qualified assessment of causality was done. This initialed document must be retained for the required regulatory time frame. The criteria below are intended as reference guidelines to assist the investigator in assessing the likelihood of a relationship between the test product and the AE based upon the available information.
3. The following components are to be used to assess the relationship between investigational agent and the AE; the greater the correlation with the components and their respective elements (in number and/or intensity), the more likely the investigational agent caused the AE:
    - **Exposure:** Is there evidence that the participant was actually exposed to the investigational agent such as: reliable history, acceptable compliance assessment (pill count, diary, etc.), expected pharmacologic effect, or measurement of drug/metabolite in bodily specimen?

- **Time Course:** Did the AE follow in a reasonable temporal sequence from administration of the investigational agent? Is the time of onset of the AE compatible with a drug-induced effect?
- **Likely Cause:** Is the AE not reasonably explained by another etiology such as underlying disease, other drug(s)/vaccine(s), or other host or environmental factors.
- **Dechallenge:** Was the investigational agent discontinued or dose/exposure/frequency reduced?

- If yes, did the AE resolve or improve?
- If yes, this is a positive dechallenge.
- If no, this is a negative dechallenge.

(Note: This criterion is not applicable if: (1) the AE resulted in death or permanent disability; (2) the AE resolved/improved despite continuation of the investigational agent; (3) the study is a single-dose drug study; or (4) the MSD product or other investigational agent is/are only used 1 time.)

- **Rechallenge:** Was the participant re-exposed to the MSD product or other investigational agent in this study?

- If yes, did the AE recur or worsen?
- If yes, this is a positive rechallenge.
- If no, this is a negative rechallenge.

(Note: This criterion is not applicable if: (1) the initial AE resulted in death or permanent disability, or (2) the study is a single-dose drug study; or (3) the MSD product(s) is/are used only 1 time.)

NOTE: IF A RECHALLENGE IS PLANNED FOR AN AE THAT WAS SERIOUS AND MAY HAVE BEEN CAUSED BY THE INVESTIGATIONAL AGENT, OR IF RE-EXPOSURE TO INVESTIGATIONAL AGENT POSES ADDITIONAL POTENTIAL SIGNIFICANT RISK TO THE PARTICIPANT THEN THE RECHALLENGE MUST BE APPROVED IN ADVANCE BY THE SPONSOR AS PER DOSE MODIFICATION GUIDELINES IN THE PROTOCOL, AND IF REQUIRED, THE INIRB/IEC.

4. **Consistency with study intervention profile:** Is the clinical/pathological presentation of the AE consistent with previous knowledge regarding the investigational agent or drug class pharmacology or toxicology?
5. The assessment of relationship will be reported on the case report forms/worksheets by an investigator who is a qualified physician according to his/her best clinical judgment, including consideration of the above elements.

6. Use the following scale of criteria as guidance (not all criteria must be present to be indicative of an investigational agent relationship).
   - Yes, there is a reasonable possibility of an investigational agent relationship:
   - There is evidence of exposure to the investigational agent. The temporal sequence of the AE onset relative to the administration of the investigational agent is reasonable. The AE is more likely explained by the investigational agent than by another cause.
   - No, there is not a reasonable possibility of the investigational agent relationship:
   - Participant did not receive the investigational agent OR temporal sequence of the AE onset relative to administration of the investigational agent is not reasonable OR the AE is more likely explained by another cause than the investigational agent. (Also entered for a participant with overdose without an associated AE.)
7. For each AE/SAE, the investigator must document in the medical notes that he/she has reviewed the AE/SAE and has provided an assessment of causality.
8. There may be situations in which an SAE has occurred and the investigator has minimal information to include in the initial report. However, it is very important that the investigator always make an assessment of causality for every event before the initial transmission of the SAE data to the Sponsor or designee.
9. The investigator may change his/her opinion of causality in light of follow-up information and send an SAE follow-up report with the updated causality assessment.
10. The causality assessment is 1 of the criteria used when determining regulatory reporting requirements.
11. For studies in which multiple agents are administered as part of a combination regimen, the investigator may attribute each AE causality to the combination regimen or to a single agent of the combination. In general, causality attribution should be assigned to the combination regimen (i.e., to all agents in the regimen). However, causality attribution may be assigned to a single agent if in the investigator’s opinion, there is sufficient data to support full attribution of the AE to the single agent.

Follow-up of AE and SAE

- The investigator is obligated to perform or arrange for the conduct of supplemental measurements and/or evaluations as medically indicated to elucidate the nature and/or causality of the AE or SAE as fully as possible. This may include additional laboratory tests or investigations, histopathological examinations, or consultation with other health care professionals.
- New or updated information will be recorded in the eCRF.

- The investigator will submit any updated SAE data to the Sponsor or designee within 24 hours and Sponsor or designee will forward updated SAE data within 1 working day to MSD Pharmacovigilance Germany and ORGANON's Local Pharmacovigilance.

Reporting of AEs, SAEs, and other reportable safety events to the sponsor

SAE reports and any other relevant safety information are to be forwarded to the sponsor or designee who will forward the report to MSD Pharmacovigilance Germany and ORGANON's Local Pharmacovigilance:

IKF Safety facsimile number: +49 / (0)69 / 7601 - 3655

or e-mail a scan to: sae@ikf-khnw.de

Appendix 4: HER2 testing in gastric cancer

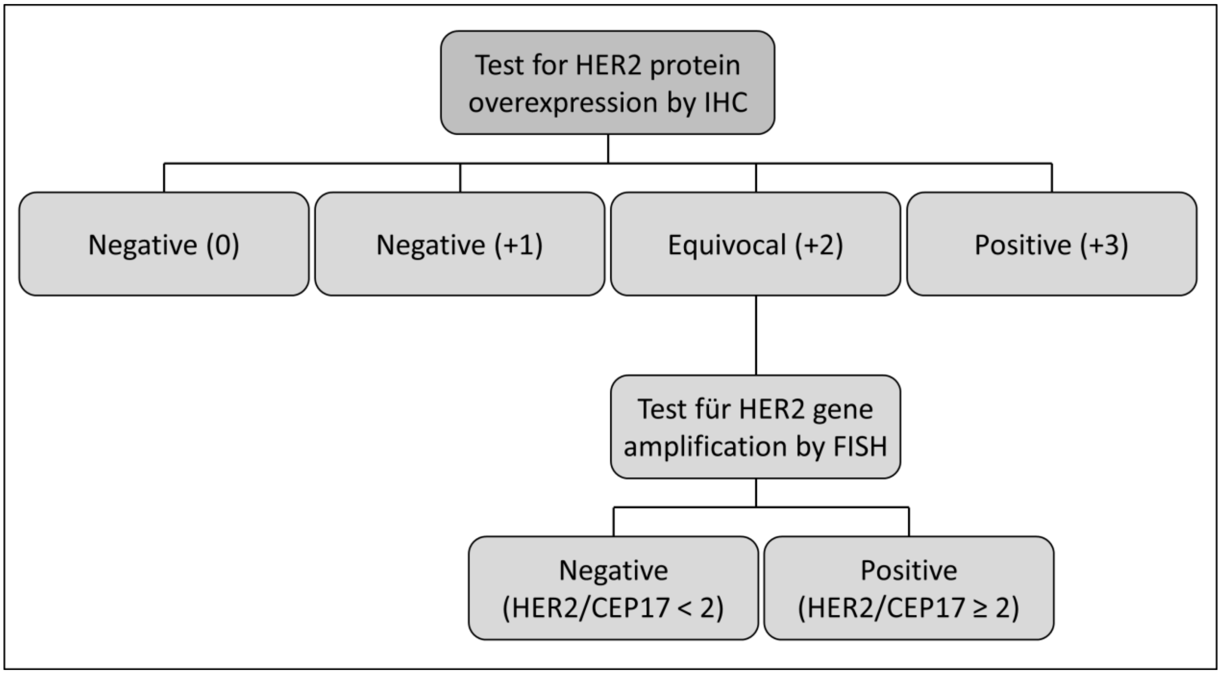

Figure 3 Appendix 3 - HER2 testing algorithm

Note: other ISH-tests than FISH (e.g. SISH or CISH) are also allowed for assessment in case of IHC 2+.

Table 12 Appendix 3 - IHC scoring for HER2

Score	Surgical Specimen	Biopsy	HER2 overexpression assessment
0	No membranous staining or staining of < 10% of the tumor cells	No membranous staining or staining only in rare cells (less than 5 cohesive cells)	Negative
1+	Staining is weak or detected in only one part of the membrane in ≥ 10% of the cells	Staining is weak or detected in only one part of the membrane of at least 5 cohesive cells	Negative
2+	Moderate/weak complete or baso-lateral membranous staining in ≥ 10% of the cells	Moderate/weak complete or baso-lateral membranous staining of at least 5 cohesive cells	Equivocal
3+	Strong complete or basolateral membranous staining in ≥ 10% of the neoplastic cells	Strong complete or basolateral membranous staining of at least 5 cohesive cells	Positive

Appendix 5: Translational research working instructions

Blood draw

Acquisition of blood in Streck® tubes

- Timepoints: prior to treatment (d0), before second pembrolizumab administration (d22), preoperatively, postoperatively and every 3 months afterwards until progression (20mL)
- Blood will be collected in 2 Streck® tubes (10 mL each) and immediately shipped using the pre-labelled envelopes to central laboratory, address and contact details are given in the sample manual.

Lab kits, including the Streck® tubes, labels and working instructions will be provided.

Tissue

Obtain paraffin embedded tissue from baseline (diagnosis) and surgery. Preferably the tumor block or alternatively up to 10 unstained slides. The tissue will be immediately shipped using the pre-labelled envelopes to central laboratory, address and contact details are given in the sample manual.

Saliva and stool collection

Stool (520 mg ± 100 mg) will be self-collected by the study participant either at home or at hospital with the help of a toilette accessory kit. Stool will be filled in a OMNIgene gut collection tube that keeps the microbiome stable for up to 60 days at room temperature. Saliva will be self-collected by the study participant with the help of a collection adapter.

- Timepoints: prior to treatment (d0, stool must be collected latest at d7 of treatment), preoperatively, after completion of adjuvant chemotherapy and 3 months after adjuvant chemotherapy
- Stool and saliva will be immediately shipped together at every timepoint of collection using the pre-labelled envelopes to:

Dr. Joseph Tintelnot
[redacted]

Collection and storage kits, labels and working instructions will be provided.

Please always refer to the most current version of the sample manual provided by the sponsor.

Pembrolizumab and trastuzumab in combination with FLOT in the perioperative treatment of HER2-positive, localized esophagogastric adenocarcinoma - A phase II trial of the AIO study group (PHERFLOT)

Statistical Analysis Plan

Sponsor: Frankfurter Institut für Klinische Krebsforschung IKF GmbH
Steinbacher Hohl 2-26
60488 Frankfurt/Main

LKP:
Dr. med. Eray Gökkurt
[redacted]

Biostatistical Analysis: Disorn Sookthai
[redacted]

SAP Version	Date	Revision Justification	Author
1.0	02.05.2025	-/-	Disorn Sookthai

Table of Contents

1. Introduction 4
2. Inclusion and exclusion criteria 5
3. Definition of analysis sets 9
4. Analysis program 10
4.1 Co-primary study endpoints 10
4.2 Secondary study endpoints 11
4.3 General statistical methods and data presentation 12
4.4 Detailed analysis procedures 13
5. Timing of analysis and interim analysis 24
6. Pre-analysis meeting(s) 25
7. Software 26
8. References 27
9. Signatures 28
Appendix 1 29

List of abbreviations

AE	Adverse Event
CI	Confidence Interval
CR	Complete Clinical Response
CRF	Case Report Form
CTC	Common Terminology Criteria
CTCAE	Common Terminology Criteria for Adverse Events
DRT	Data Record Table
DSF	Disease free Survival
ECOG	Eastern Cooperative Oncology Group
eCRF	Electronic Case Report Form
F	Full Analysis Set
HR	Hazard Ratio
ICH	International Conference on Harmonization of Technical Requirements for Registration of Pharmaceuticals
ITT	Intention to Treat
LKP	Clinical Trial Director / Principal Investigator according to AMG (Leiter der Klinischen Prüfung)
NCI	National Cancer Institute
ORR	Overall Response Rate
OS	Overall Survival
PAM	Pre-analysis Meeting
PD	Progressive Disease
PP	Per Protocol (Set)
PR	Partial Response
RECIST	Response Evaluation Criteria In Solid Tumors
S	Safety Analysis (Set)
SAE	Serious Adverse Event
SAP	Statistical Analysis Plan
SD	Stable Disease
SOP	Standard Operating Procedure

1. INTRODUCTION

This statistical analysis plan (SAP) is based on the study protocol V. 1.3 dating from 14-Mar-2024. It specifies the complete analytical procedures to be performed at the planned final analyses.

Methodologically, the analysis will follow the outlines of the protocol, and the ICH Guideline E9 "Statistical principles for clinical trials". The scope and contents of the SAP are primarily described in sections 3 (Objective(s), Hypothesis(es), and Endpoint(s)) and 9 (Statistical Analysis Plan) of the protocol.

The analysis report(s) will be written in English language in MS WORD and PDF format.

Amendments to this SAP may be required during the study but should be finalized after the pre-analysis meeting (PAM, also called “Data Review Meeting”,) and before embarking on the analysis of any efficacy or safety objectives.

2. INCLUSION AND EXCLUSION CRITERIA

Inclusion criteria

1. The participant provides written informed consent for the trial.
2. Male/female participants who are at least 18 years of age on the day of signing informed consent. There are no data that indicate special gender distribution. Therefore, patients will be enrolled in the study gender-independently.
3. In the investigator's judgement, participant is willing and able to comply with the study protocol including the planned surgical treatment
4. Histologically confirmed adenocarcinoma of the GEJ (Type I-III according to Siewert´s classification) or the stomach (cT2, cT3, cT4, any N category, M0, or any T, N+, M0) that:
   - is not infiltrating any adjacent organs or structures by CT or MRI evaluation
   - does not involve peritoneal carcinomatosis
   - is considered medically and technically resectable

Note: the absence of distant metastases must be confirmed by CT or MRI of the thorax and abdomen, and, if there is clinical suspicion of osseous lesions, a bone scan. If peritoneal carcinomatosis is suspected clinically, its absence must be confirmed by laparoscopy. Diagnostic laparoscopy is mandatory in patients with T3 or T4 tumors of the diffuse type histology in the stomach.

Participants must have HER2-positive disease defined as either IHC 3+ or IHC 2+, the latter in combination with ISH+, as assessed locally by a certified test on primary tumor (see Appendix 4 of study protocol)

6. Participants must be candidates for potential curative resection as determined by the treating surgeon
7. No prior systemic-anti cancer therapy (e.g. cytotoxic or targeted agents or radiotherapy)
8. No prior partial or complete esophagogastric tumor resection
9. ECOG (Eastern Cooperative Oncology Group) performance status score of 0 or 1
10. Male participants: A male participant must agree to use a contraception as detailed in Appendix 2 of this protocol during the treatment period and for at least 6 months after the last dose of study intervention and refrain from donating sperm during this period.

Female participants: A female participant is eligible to participate if she is not pregnant (see Appendix 2 of study protocol), not breastfeeding, and at least one of the following conditions applies:

- Not a woman of childbearing potential (WOCBP) as defined in Appendix 2

OR

- A WOCBP who agrees to follow the contraceptive guidance as given in Appendix 2 of study protocol during the treatment period and for at least 7 months after the last dose of study intervention.

11. Participants have adequate organ function as defined in the following table (Table 1). Specimens must be collected within 14 days prior to enrolment (also to be repeated if older than 14 days at day of first treatment).

Table 1 Adequate organ function laboratory values

System	Laboratory Value
Hematological	
Absolute neutrophil count (ANC)	≥ 1500/µL
leucocytes	≥ 3000/µL
Thrombocytes	≥ 100000/µL
Hemoglobin	≥ 9.0 g/dL or ≥ 5.6 mmol/L[a]
Renal: Measured or calculated[b] creatinine clearance (CrCl)	≥ 50 mL/min
Hepatic	
Total bilirubin	≤ 1.5 ×ULN OR direct bilirubin ≤ ULN for participants with total bilirubin levels > 1.5 × ULN
AST (SGOT) and ALT (SGPT)	≤ 2.5 × ULN
Coagulation	
International normalized ratio (INR) OR prothrombin time (PT) and activated partial thromboplastin time (aPTT)	≤ 1.5 × ULN unless participant is receiving anticoagulant therapy as long as PT or aPTT is within therapeutic range of intended use of anticoagulants
ALT (SGPT)=alanine aminotransferase (serum glutamic pyruvic transaminase); AST (SGOT)=aspartate aminotransferase (serum glutamic oxaloacetic transaminase); GFR=glomerular filtration rate; ULN=upper limit of normal.<br>[a] Criteria must be met without erythropoietin dependency and without packed red blood cell (pRBC) transfusion within the last 2 weeks.<br>[b] CrCl should be calculated per institutional standard.	

Exclusion criteria

1. Participants with involved retroperitoneal (e.g. para-aortal, paracaval or interaortocaval lymph nodes) or mesenterial lymph nodes (distant metastasis!)
2. A WOCBP who has a positive urine pregnancy test within 72 hours prior to start of study intervention (see Appendix 2). If the urine test is positive or cannot be confirmed as negative, a serum pregnancy test will be required.
3. Received prior therapy with an anti-PD-1, anti-PD-L1, or anti PD L2 agent or with an agent directed to another stimulatory or co-inhibitory T-cell receptor (e.g., CTLA-4, OX 40, CD137).
4. Participant received colony-stimulating factors (e.g. granulocyte colony-stimulating factor [G-CSF], granulocyte-macrophage colony-stimulating factor [GM CSF] or recombinant erythropoietin) within 28 days prior to the first dose of study intervention.
5. Major surgery within 2 weeks of starting study intervention and patients must have recovered from any effects of any major surgery.
6. Concomitant use of drugs inhibiting (dihydropyrimidine dehydrogenase) DPD activity (including sorivudine, brivudine), the required wash out phase is 4 weeks before start of the study intervention.

7. Inadequate cardiac function (LVEF value < 55 %) as determined by echocardiography
8. Resting ECG indicating uncontrolled, potentially reversible cardiac conditions, as judged by the investigator (e.g., unstable ischemia, uncontrolled symptomatic arrhythmia, congestive heart failure, QTcF prolongation > 500 ms, electrolyte disturbances, etc.), or patients with congenital long QT syndrome.
9. Participant has received a live vaccine or live-attenuated vaccine within 30 days prior to the first dose of study drug. Administration of killed vaccines is allowed.
10. Participant is currently participating in or has participated in a study of an investigational agent within 4 weeks or within less than 5 half-lives of the investigational agent (whichever is longer) or has used an investigational device within 4 weeks prior to the first dose of study intervention.
11. Participant has a diagnosis of immunodeficiency or is receiving chronic systemic steroid therapy (in dosing exceeding 10 mg daily of prednisone equivalent) or any other form of immunosuppressive therapy within 7 days prior to the first dose of study drug.
12. Participant has a known additional malignancy that is progressing or has required active treatment within the past 2 years. Participants with basal cell carcinoma of the skin, squamous cell carcinoma of the skin or carcinoma in situ (e.g., breast carcinoma, cervical cancer in situ) that have undergone potentially curative therapy are not excluded.
13. Participant has myelodysplastic syndrome (MDS)/acute myeloid leukemia (AML) or with features suggestive of MDS/AML.
14. Participant has severe dyspnea at rest requiring supplementary oxygen therapy.
15. History of severe allergic, anaphylactic, or other hypersensitivity reactions to chimeric or humanized antibodies or fusion protein; known hypersensitivity to Chinese hamster ovary cell products or to any component of the pembrolizumab or trastuzumab formulation
16. Any known contraindication (including hypersensitivity) to docetaxel, 5-FU, folinic acid/leucovorin, or oxaliplatin.
17. Known DPD deficiency. Patients with a reduced DPD activity (CPIC activity score of 1.0-1.5) might participate in the study and receive a reduced dosage of 5-FU after discussion with the coordinating investigator and sponsor [https://cpicpgx.org/guidelines/guideline-for-fluoropyrimidines-and-dpyd/]
18. Participant has active autoimmune disease that has required systemic treatment in the past 2 years (i.e., with use of disease modifying agents, corticosteroids or immunosuppressive drugs). Replacement therapy (e.g., thyroxine, insulin, or physiologic corticosteroid replacement therapy for adrenal or pituitary insufficiency, etc.) is not considered a form of systemic treatment and is allowed.
19. Participant has a history of (non-infectious) pneumonitis/interstitial lung disease that required steroids or has current pneumonitis/interstitial lung disease.
20. Participant has an active infection requiring systemic therapy.
21. Participant has a known history of Human Immunodeficiency Virus (HIV) infection
22. Participant has a known history of Hepatitis B (defined as Hepatitis B surface antigen [HBsAg] reactive) or known active Hepatitis C virus (defined as HCV RNA is detected) infection.
23. Participant is considered a poor medical risk due to a serious, uncontrolled medical disorder, non-malignant systemic disease or active, uncontrolled infection. Examples include, but are not limited to, uncontrolled ventricular arrhythmia, recent (within 3 months) myocardial infarction, uncontrolled major seizure disorder, unstable spinal cord compression, superior vena cava syndrome, extensive interstitial bilateral lung disease on High Resolution Computed Tomography

(HRCT) scan, previous allogenic bone marrow/blood transplantation or any psychiatric disorder or substance abuse that prohibits obtaining informed consent.

24. Participant is pregnant or breastfeeding or expecting to conceive or father children within the projected duration of the study, starting with the screening visit through 6 months after the last dose of study intervention.
25. Participant has had an allogenic tissue/solid organ transplant.

3. DEFINITION OF ANALYSIS SETS

Full analysis set (FAS)

The Full analysis set (FAS) will include all patients who received at least one treatment dose.

Per-protocol (PP) population

The per-protocol population includes all patients of the FAS population who completed the trial without any major protocol violations. The PP population will be used for sensitivity analysis purpose in order to assess the robustness of the results obtained from the FAS population.

Safety (S) population

Toxicity analyses will be based on the safety population which is identical to FAS.

4. Analysis program

4.1 Co-primary study endpoints

The trial is designed as single arm, multicenter, open-label, phase II study, which aims to show therapeutic efficacy of the experimental regimen pembrolizumab and trastuzumab in combination with FLOT. The co-primary endpoints are the pathological complete response rate (pCR) and the disease-free survival rate after 2 years (DFSR@2).

We hypothesize that the DFS rate after 2 years (DFSR@2) should be 70% or more and as an interim efficacy analysis (to be read out after surgery of the last patient) pCR rate is 30% or greater. DFSR@2 is defined as the proportion of patients being tumor/disease free and alive 2 years after enrolment. The pCR rate, defined as the absence of residual tumor based on evaluation of the resected esophagogastric specimen in the primary by local pathology

The efficacy assumptions can be obtained from the PETRARCA and the HER-FLOT study [1,2].In the PETRARCA study pCR rate in the standard FLOT arm was 12%. This could be increased to 22% with trastuzumab plus FLOT in the HER-FLOT trial and to 35% with trastuzumab, pertuzumab and FLOT in the PETRARCA trial. We envisage that the pCR rate for pembrolizumab, trastuzumab and FLOT could increase to 30% or more.

Hence, for the experimental regimen to be considered as a desirable candidate for further development, the pCR of 30% should be achieved, but if it is 12% or less the experimental arm would be insufficient for further development.
Formally, the hypothesis testing for the first co-primary endpoint can be defined as:

H0: $P \leq 0.12$ versus H1: $P \geq 0.30$

The current sample size calculation is based on pCR improvement from 12% to 30% with a one-sided alpha of 5% and a beta of 20% in a Fleming single stage phase II procedure. Thus, 27 patients are required.

Concurrently, for DFS the respective sample size calculation would be an improvement of DFS rate at 2 years from 50% (results of the FLOT 4 trial) to 70% the hypothesis testing for the second co-primary endpoint can be defined as:
H0: $P \leq 0.5$ versus H1: $P \geq 0.7$

with a one-sided alpha of 0.1 and 80% power in a Fleming single stage phase II procedure, 27 patients are needed.

Thus, a sample size of 27 patients is sufficient for both co-primary endpoints considering that the co-primary endpoints are homogeneous, no inflation of type II error is expected. We therefore keep the statistical power at 80% for both co-primary endpoints.

Considering a drop-out rate of 10%, a total number of 30 patients will be enrolled.

We hypothesize that the DFS rate after 2 years (DFSR@2) should be 70% or more and pCR rate is 30% or greater. DFSR@2 is defined as the proportion of patients being tumor/disease free and alive 2 years after enrolment. The pCR rate is defined as the absence of residual tumor based on evaluation of the resected esophagogastric specimen in the primary tumor by local pathology.

4.2 Secondary study endpoints

In general, the secondary parameters are analysed descriptively and exploratively:

- Assessment of overall response rate, defined as percentage of patients with CR and PR according to RECIST v1.1
- R0 resection rate, where R0 resection is defined as - microscopically margin negative resection with no gross or microscopic tumor remains in the areas of the primary tumor and/or sampled regional lymph nodes
- Overall survival (OS) where OS is defined as time from enrolment to the date of death of any cause. If no event is observed (e.g., lost to follow-up) OS is censored at the date of last subject contact. Subjects who are alive will be censored at the last known alive dates.
- Disease-free survival defined as the time from enrolment to disease progression according to RECIST v1.1 or relapse after surgery or death from any cause. Patients without an event will be censored at the date of their last tumor assessment.
- Feasibility rate defined as a rate of patient whom received a treatment regimen of pembrolizumab and trastuzumab in combination with FLOT 8 weeks pre- as well as post-surgery, followed by pembrolizumab and trastuzumab treatment for up to one year without severe toxicity/withdrawal rate resulting in discontinued the treatments for a reason other than progressive disease
- Pathological complete and subtotal regression (TRG1a/b by Becker). TRG1a/b is defined as < 10% residual tumor per tumor bed based on evaluation of the resected esophagogastric specimen in the primary by local pathology.
- Perioperative morbidity and mortality
- Safety and toxicity: Adverse events will be recorded and graded according to version 5.0 of National Cancer Institute Common Toxicity Criteria (NCI-CTC). Occurrence of any adverse event and occurrence of any serious adverse event (anytime during the study) will be presented. These events will also be described by nature (Primary System Organ Class and Preferred Term), severity and causal relationship to drug administration.

4.3 General statistical methods and data presentation

Statistical analysis is based on the International Conference on Harmonization (ICH) Guidelines "Structure and Content of Clinical Study Reports" and "Statistical Principles for Clinical Trials". Due to the explorative nature of this trial and the small number of patients only descriptive statistics will be performed (e.g., describing the distribution of the baseline demographic data with predefined subgroups). Missing data will not be extrapolated. The number of missing values will be computed. For the time-to-event variables time to progression (TTP), PFS/DFS and OS, the Kaplan-Meier method will be used.

All parameters will be evaluated in an explorative or descriptive manner, providing means, medians, interquartile and total ranges, standard deviations and/or confidence intervals, absolute and relative frequencies, or Kaplan-Meier curves, as appropriate for the respective data types.

In general, all analyses will be presented, and calculations performed based on the data actually available for each item (observed case analyses). Incomplete time-to-event observations will be handled as censored measurements and missing data will be considered as failure for the primary endpoint.

If additional p-values for differences between (sub)groups are calculated for selected items, they will be presented explicitly without referring to pre-specified hypotheses or a significance level. Usually, no error adjustment for multiple testing will be performed in an explorative analysis setting. Thus, the p-values will reflect the comparison-wise error and not the experiment-wise error. All p-values will be two-sided, if not otherwise defined or stated. The statistical methods described in this section are suited for the data and distributions usually expected in this type of trials.

Event related data like progression-free and overall survival will be estimated by the product limit method [3], providing the numbers of events and censored cases, median survival time along with its 95% CI (if applicable).

All safety parameters will be evaluated in an explorative or descriptive manner, providing proportions, means, medians, ranges, standard deviations and/or confidence intervals, as appropriate. The analyses will focus on the adverse events categorized and graded according to NCI CTCAE v5.0. Adverse events will be summarized by preferred term, intensity, and causal relationship to the study agent, with frequencies and percentages reported.

There is no full interim analysis planned for this study, due to the small sample size and the relatively short recruitment period. However, single objectives may be analyzed as soon as sufficient events are available for analysis.

4.4 Detailed analysis procedures

A description of the analyses to be performed is provided in the following tables. The contents of the final study report will correspond to this structure. The short names identifying the eCRF page are explained in appendix 1.

PARAMETER / ANALYSIS DESCRIPTION	CRF PAGE	ANALYSIS SET	METHODS	COMMENT
**GENERAL**				
Description of study/analysis population and evaluability with respect to eligibility, available forms and information throughout the course of protocol therapy (corresponding to a diagram)				Based on information from monitoring and PAM
Definition/identification of FAS, PP, and S analysis sets				
Violation of inclusion/exclusion criteria and other protocol violations leading to exclusion or censoring in primary endpoint analysis	IE			List provided by monitoring / data management (for PAM) SF_REAS
Creation of files representing the study/analysis populations				
Course of recruitment	IE	F	Figure	ENR_DAT
Patient distribution by center	IE	F	DA	MNPAID
**DEMOGRAPHIC DATA / GENERAL STATUS**				
Age	DM	F	DA, HIS	AGE_CALC
Sex	DM	F	DA	DMSEX
ECOG performance status	PE	F	DA	ECOG
**BASELINE, TUMOR DATA**				
Time since first diagnosis of esophagogastric carcinoma	ANAM, IE	F	DA	ENR_DAT - ANAM_DAT
Localization of primary	ANAM	F	DA	ANAM_LOC
Lauren classification	ANAM	F	DA	ANAM_LCL
Signet ring cells presence? (y/n)	ANAM	F	DA	ANAM_SIGC
Is there a Barrett carcinoma of distal oesophagus? (y/n)	ANAM	F	DA	ANAM_BARCA
Histopathological grade	ANAM	F	DA	HIST_GR
TNM at first diagnosis: cT	ANAM	F	DA	TNM_T
TNM at first diagnosis: cN	ANAM	F	DA	TNM_N

PARAMETER / ANALYSIS DESCRIPTION	CRF PAGE	ANALYSIS SET	METHODS	COMMENT
TNM at first diagnosis: cM	ANAM	F	DA	TNM_M
Localization of lymph nodes	ANAM	F	DA	ANAM_LNPERI, ANAM_LNTRUN, ANAM_LNLIV, ANAM_LNHEP, ANAM_LNLOW, ANAM_LNPARA, ANAM_LNPARAT, ANAM_LNUPP, ANAM_LNOTH
If other localization of lymph nodes	ANAM	F	LISTING	ANAM_LNOTHTXT
Was diagnostic laparoscopy performed? (y/n)	ANAM	F	DA	ANAM_LAPPERF
Reason if no diagnostic laparoscopy performed	ANAM	F	LISTING	ANAM_LAPPERFNREAS
Time since diagnostic laparoscopy	ANAM, IE	F	DA	ENR_DAT - ANAM_LAPDAT
Absence of peritoneal carcinomatosis confirmed?	ANAM	F	DA	ANAM_LAPRES
Reason(s) if absence of peritoneal carcinomatosis cannot be confirmed	ANAM	F	LISTING	ANAM_LAPRESNREAS
Was HER-2 assessment performed? (y/n)	ANAM	F	DA	ANAM_HER2PERF
Immunhistochemistry	ANAM	F	DA	ANAM_HER2IHRES
In Situ Hybridization (ISH) (FISH or SISH/CISH)	ANAM	F	DA	ANAM_HER2ISHRES
Patient HER-2 classification	ANAM	F	DA	ANAM_HER2RES
Was local PD-L1 assessment performed? (y/n)	ANAM	F	DA	ANAM_PDL1PERF
Time since PD-L1 assessment	ANAM	F	DA	ENR_DAT - ANAM_PDL1DAT
CPS (Combined Positive Score)	ANAM	F	DA	ANAM_PDL1CPSRES
TPS (Tumor Proportion Score)	ANAM	F	DA	ANAM_PDL1TPSRES, ANAM_PDL1TPSUNK
IC (Immune Cell Score)	ANAM	F	DA	ANAM_PDL1ICRES, ANAM_PDL1ICUNK
MSI/Mismatch Repair status available (y/n)	ANAM	F	DA	ANAM_MSIPERF
Result of IHC (immunohistochemistry)	ANAM	F	DA	ANAM_MSIIHCRES
Result of PCR	ANAM	F	DA	ANAM_MSIPCRRES
MSI if other Result	ANAM	F	LISTING	ANAM_MSIOTHTXT
DPD (CPID activity score)	ANAM	F	DA	ANAM_DPDRES
**MEDICAL HISTORY**				
Are there any relevant medical history conditions? (y/n)	MH	F	DA	MHYN

PARAMETER / ANALYSIS DESCRIPTION	CRF PAGE	ANALYSIS SET	METHODS	COMMENT
Concomitant diseases	EMH	F	DA	MH_TERM
Are there any active medical history conditions? (y/n)	MH, EMH	F	DA	MHYN, MH_STAT
Concomitant diseases at study entry	EMH	F	DA	MH_TERM, MH_STAT
Concomitant diseases treated with medication at study entry at least once (y/n)	EMH	F	DA	MH_CM
**TREATMENTS**				
IMP administered at least once	TRT	F	DA	TRTPERFYN
No. of IMP administered cycles	TRT	F	DA	TRTPERFYN
Reasons if IMP not administered	TRT	F	DA	TRTPERFNREAS
Other reasons if IMP not administered	TRT	F	LISTING	TRTPERFNREASOTHTXT
if IMP not administered due to toxicity	TRT	F	LISTING	TRTPERFNREASTOXTXT
FLOT administered at least once	TRT	F	DA	FLOTPERFYN
No. of FLOT administered cycles	TRT	F	DA	FLOTPERFYN
Reasons if FLOT not administered	TRT	F	DA	FLOTPERFNREAS
Other reasons if FLOT not administered	TRT	F	LISTING	FLOTPERFNREASOTHTXT
if FLOT not administered due to toxicity	TRT	F	LISTING	FLOTPERFNREASTOXTXT
Docetaxel administered at least once	TRT	F	DA	DOCEYN
No. of docetaxel administered cycles	TRT	F	DA	DOCEYN
Reasons if docetaxel not administered	TRT	F	DA	DOCENREAS
Other reasons if docetaxel not administered	TRT	F	LISTING	DOCENREASOTHTXT
if docetaxel not administered due to toxicity	TRT	F	LISTING	DOCENREASTOXTXT
Cumulative and average docetaxel administered dose	TRT	F	DA	DOCEDOS, DOCNEWDOS
Docetaxel interruption at least once (y/n)	TRT	F	DA	DOCEINTERYN
Type of interruption or modification for docetaxel	TRT	F	DA	DOCEINTERTYP
Reason(s) for interruption or modification for docetaxel	TRT	F	DA	DOCEINTERREAS
If other interruption reason for docetaxel	TRT	F	LISTING	DOCEINTERTXT
Docetaxel new dose	TRT	F	DA	DOCNEWDOS
Oxaliplatin administered at least once	TRT	F	DA	OXAPERFYN
No. of oxaliplatin administered cycles	TRT	F	DA	OXAPERFYN, MNPVISLABEL
Reasons if oxaliplatin not administered	TRT	F	DA	OXANREAS

PARAMETER / ANALYSIS DESCRIPTION	CRF PAGE	ANALYSIS SET	METHODS	COMMENT
Other reasons if oxaliplatin not administered	TRT	F	LISTING	OXANREASOTHTXT
if oxaliplatin not administered due to toxicity	TRT	F	LISTING	OXANREASTOXTXT
Cumulative and average oxaliplatin administered dose	TRT	F	DA	OXADOS, OXANEWDOS
Oxaliplatin interruption at least once (y/n)	TRT	F	DA	OXAINTERYN
Type of interruption or modification for oxaliplatin	TRT	F	DA	OXAINTERTYP
Reason(s) for interruption or modification for oxaliplatin	TRT	F	DA	OXAINTERREAS
If other interruption reason for oxaliplatin	TRT	F	LISTING	OXAINTERTXT
Oxaliplatin new dose	TRT	F	DA	OXANEWDOS
Folinic acid administered at least once	TRT	F	DA	FOLPERFYN
No. of folinic acid administered cycles	TRT	F	DA	FOLPERFYN, MNPVISLABEL
Reasons if folinic acid not administered	TRT	F	DA	FOLPERFNREAS
Other reasons if folinic acid not administered	TRT	F	LISTING	FOLPERFNREASOTHTXT
if folinic acid not administered due to toxicity	TRT	F	LISTING	FOLPERFNREASTOXTXT
Cumulative and average folinic acid administered dose	TRT	F	DA	FOLDOS, FOLNEWDOS
Folinic acid interruption at least once (y/n)	TRT	F	DA	FOLINTERYN
Type of interruption or modification for folinic acid	TRT	F	DA	FOLINTERTYP
Reason(s) for interruption or modification for folinic acid	TRT	F	DA	FOLINTERREAS
If other interruption reason for folinic acid	TRT	F	LISTING	FOLINTERTXT
Folinic acid new dose	TRT	F	DA	FOLNEWDOS
5-FU administered at least once	TRT	F	DA	T5FUPERFYN
No. of 5-FU administered cycles	TRT	F	DA	T5FUPERFYN, MNPVISLABEL
Reasons if 5-FU 5-FU not administered	TRT	F	DA	T5FUPERFNREAS
Other reasons if 5-FU not administered	TRT	F	LISTING	T5FUPERFNREASOTHTXT
if 5-FU not administered due to toxicity	TRT	F	LISTING	T5FUPERFNREASTOXTXT
Cumulative and average 5-FU administered dose	TRT	F	DA	T5FUDOS, T5FUNEWDOS
5-FU interruption at least once (y/n)	TRT	F	DA	T5FUINTERYN
Type of interruption or modification for 5-FU	TRT	F	DA	T5FUINTERTYP

PARAMETER / ANALYSIS DESCRIPTION	CRF PAGE	ANALYSIS SET	METHODS	COMMENT
Reason(s) for interruption or modification for 5-FU	TRT	F	DA	T5FUINTERREAS
If other interruption reason for 5-FU	TRT	F	LISTING	T5FUINTERTXT
5-FU acid new dose	TRT	F	DA	T5FUNEWDOS
Pembrolizumab administered at least once	TRT	F	DA	PEMPERFYN
No. of pembrolizumab administered cycles	TRT	F	DA	PEMPERFYN, MNPVISLABEL
Reasons if pembrolizumab not administered	TRT	F	DA	PEMPERFNREAS
Other reasons if pembrolizumab not administered	TRT	F	LISTING	PEMPERFNREASOTHTXT
if pembrolizumab not administered due to toxicity	TRT	F	LISTING	PEMPERFNREASTOXTXT
Cumulative and average pembrolizumab administered dose	TRT	F	DA	PEMDOS
Pembrolizumab interruption at least once (y/n)	TRT	F	DA	PEMINTERYN
Pembrolizumab interruption type	TRT	F	DA	PEMINTERTYP
Pembrolizumab interruption reason	TRT	F	DA	PEMINTERREAS
If other interruption reason for pembrolizumab	TRT	F	LISTING	PEMINTERTXT
Trastuzumab administered at least once	TRT	F	DA	TRAPERFYN
No. of trastuzumab administered cycles	TRT	F	DA	TRAPERFYN, MNPVISLABEL
Reasons if trastuzumab not administered	TRT	F	DA	TRAPERFNREAS
Other reasons if trastuzumab not administered	TRT	F	LISTING	TRAPERFNREASOTHTXT
if trastuzumab not administered due to toxicity	TRT	F	LISTING	TRAPERFNREASTOXTXT
Cumulative and average trastuzumab administered dose	TRT	F	DA	TRADOS
Trastuzumab interruption at least once (y/n)	TRT	F	DA	TRAINTERYN
Trastuzumab interruption type	TRT	F	DA	TRAINTERTYP
Trastuzumab interruption reason	TRT	F	DA	TRAINTERREAS
If other interruption reason for trastuzumab	TRT	F	LISTING	TRAINTERTXT
**SURGERY**				
Was surgery performed? (y/n)	SURG	F	DA	SURGYN
Reason(s) if surgery was not performed.	SURG	F	LISTING	SURGNREAS
Was resection performed? (y/n)	SURG	F	DA	RESECYN
Reason(s) if resection was not performed.	SURG	F	LISTING	RESECNREAS
Time from enrolment until surgery	IE, SURG	F	DA	SURGDAT- ENR_DAT

PARAMETER / ANALYSIS DESCRIPTION	CRF PAGE	ANALYSIS SET	METHODS	COMMENT
Surgery after 4-6 weeks after last treatment? (y/n)	SURG	F	DA	SURGPLAN
Surgery duration	SURG	F	DA	SURGDUR
Surgery specifics	SURG	F	LISTING	SURGSPECTXT
Reason(s) if surgery specifics none	SURG	F	DA	SURGSPECN
Reason(s) if surgery not as scheduled,	SURG	F	LISTING	SURGPLANNREAS
Duration of hospital stay	SURG	F	DA	SURGIHOSPENDAT-SURGHOSPSTDAT
Ventilated? (y/n)	SURG	F	DA	SURGVENTYN
Ventilated duration	SURG	F	DA	SURGVENTDUR
Complications? (y/n)	SURG	F	DA	SURGCOM
Surgical complication	SURG	F	DA	SURGCOMSURG
Medical complication	SURG	F	DA	SURGCOMMED
Inpatient stay duration	SURG	F	DA	SURGHOSPDUR
Medical complication specify	SURG	F	DA	SURGCOMPOH, SURGCOMANL, SURGCOMABS, SURGCOMHAE, SURGCOMFIS, SURGCOMLOCOTH
If abscess, please specify:	SURG	F	LISTING	SURGCOMABSTXT
If other, please specify:	SURG	F	LISTING	SURGCOMLOCOTHTXT
Local complications score	SURG	F	DA	SURGCOMLOSCORE
Local complications specify	SURG	F	DA	SURGCOMPNEU, SURGCOMCAIN, SURGCOM1OF, SURGCOM2OF, SURGCOMMOF, SURGCOMSEP, SURGCOMHDF, SURGCOMCARD, SURGCOMRESFA, SURGCOMARDS, SURGCOMRENFA, SURGCOMHEPFA, SURGCOMCOA, SURGCOMSYSOTH
If other local complications, please specify:	SURG	F	LISTING	SURGCOMSYSOTHSPEC
HDF / dialysis duration	SURG	F	DA	SURGCOMHDFDUR
Systemic complications score	SURG	F	DA	SURGCOMSYSCO
Systemic complications specify	SURG	F	DA	SURGINTYN, SURGEXLEYN, SURGRESURGYN

PARAMETER / ANALYSIS DESCRIPTION	CRF PAGE	ANALYSIS SET	METHODS	COMMENT
Intervention occurred specify on which post-OP day	SURG	F	LISTING	SURGINTYTXT, SURGINTPOSTOPD
**HISTOLOGY**				
Resection stomach or GEJ? (y/n)	HIST	F	DA	HISTRESEC
If other resection	HIST	F	LISTING	HISTRESECOTHSPEC
If non-resectional surgery	HIST	F	DA	HISTNRESEC
If other non-resectional surgery	HIST	F	LISTING	HISTNRESECOTHSPEC
Lymphadenectomy	HIST	F	DA	HISTLYMPHA
No. of lymph node removed	HIST	F	DA	HISLTYMNOREM
Splenectomy performed (y/n)	HIST	F	DA	HISTSPLENYN
Histological diagnosis	HIST	F	DA	HISTDIAG
If other histological diagnosis	HIST	F	LISTING	HISTDIAGOTHTXT
Lauren classification	HIST	F	DA	HISTLAUREN
Signet cells	HIST	F	DA	HISTSIGNET
T-stage stomach	HIST	F	DA	HISTTSTAGESTOM
N-stage stomach	HIST	F	DA	HISTNSTAGESTOM
M-stage stomach	HIST	F	DA	HISTMSTAGESTOM
T-stage GEJ	HIST	F	DA	HISTTSTAGEGEJ
N-stage GEJ	HIST	F	DA	HISTNSTAGEGEJ
M-stage GEJ	HIST	F	DA	HISTMSTAGEGEJ
Residual tumor	HIST	F	DA	HISTRESTUM
Location primary	HIST	F	DA	HISTLOCPRIM
Lymphangiosis? (y/n)	HIST	F	DA	HISTLYMPHAYN
Angioinvasion? (y/n)	HIST	F	DA	HISTANGIOYN
Stomach carcinoma	HIST	F	DA	HISTAJCCSTOM
GEJ I-III	HIST	F	DA	HISTAJCCGEJ
Pathological response in the primary	HIST	F	DA	HISTPATRESP
Comment on primary pathological response	HIST	F	LISTING	PRIMCOM
Pathological response in the lymph node	HIST	F	DA	HISTPATRESLN
Comment on lymph node pathological response	HIST	F	LISTING	LNCOM
Lymph nodes	HIST	F	DA	HISTPATRESLNYN
**END OF PROTOCOL TREATMENT / END OF STUDY**				
Reasons(s) for end of therapy	EOT	F	DA	EOT_REAS
Premature end of protocol treatment	EOT	F	DA	EOS_REAS ≠ 1,2,3

PARAMETER / ANALYSIS DESCRIPTION	CRF PAGE	ANALYSIS SET	METHODS	COMMENT
Follow-up time per patient (for whole population and for surviving patients only)	EOS, IE, FU	F	DA	Max (FU_DAT, EOS_REASDTH_DAT, EOS_DAT) – ENR_DAT;<br><br>Max (FU_DAT, EOS_REASDTH_DAT, EOS_DAT) - ENR_DAT where EOS_REAS ≠1;
Reason for end of study	EOS	F	DA	EOS_REAS
If other reasons for end of study	EOS	F	LISTING	EOS_REASOTHTXT
Causes of death	EOS	F	DA	EOS_REASDTH_REAS
If other causes of death	EOS	F	LISTING	EOS_REASDTH_TXT
Was there any Follow-up contact?	EOS	F	DA	EOS_FUYN
Were there any AEs reported during first administration until the safety follow-up visit (28 ± 3 days after the last dose of study treatment) or until initiation of another anti-cancer therapy?	EOS	F	DA	EOS_AEYN
**EFFICACY ANALYSIS (CO-PRIMARY ENDPOINTS)**				
Disease-free survival rate at 2 years (DFSR@2) defined as the proportion of patients being tumor/disease free and alive 2 years after enrolment.	EOS, FU, TA, IE	F, PP	DA, T, CI	TA_IMGDAT, RECIST_OVERALL, FU_PDTYP, FU_PDDAT, EOS_REASDTH_DAT, ENR_DAT
Pathological complete regression (pCR) rate defined as the absence of residual tumor based on evaluation of the resected esophagogastric specimen in the primary tumor by local pathology.	HIST	F, PP	DA, T, CI	HISTPATRESP
**EFFICACY ANALYSIS ON TIME TO EVENT (SECONDARY ENDPOINTS)**				
Disease-free survival defined as the time from enrolment to disease progression according to RECIST v1.1 or relapse after surgery or death from any cause. Patients without an event will be censored at the date of their last tumor assessment.	EOS, FU, TA, IE	F, PP	DA, FIGURE	median survival time with 95CI%, KM-plot<br><br>TA_IMGDAT, RECIST_OVERALL, FU_PDTYP, FU_PDDAT, EOS_REASDTH_DAT, ENR_DAT

Parameter / Analysis Description	CRF Page	Analysis Set	Methods	Comment
Overall survival defined as time from study enrolment to the date of death of any cause. If no event is observed (e.g., lost to follow-up) OS is censored at the date of last subject contact. Subjects who are alive will be censored at the last known alive dates.	EOS, FU, SURV, RND	F, PP	DA, FIGURE	median survival time with 95CI%, KM-plot<br><br>FU_DAT, EOS_REASDTH_DAT, EOS_DAT<br><br>ENR_DAT
**EFFICACY ANALYSIS (SECONDARY ENDPOINTS)**				
Overall response rate (ORR) according to RECIST v1.1	TA	F, PP	DA, CI	RECIST_OVERALL
Best overall response (all response categories) according to RECIST v1.1	TA	F, PP	DA	RECIST_OVERALL
Resection rate (R0)	HIST	F, PP	DA, CI	HISTRESTUM
Resection rate (all response categories)	HIST	F, PP	DA	HISTRESTUM
Rate of pathological complete and subtotal regression (pCR+pSR TRG1a/b) as assessed according to the Becker criteria	HIST	F, PP	DA, CI	HISTPATRESP
Pathological regression for all response categories	HIST	F, PP	DA	HISTPATRESP
Rate of pathological complete and subtotal remission (pCR+pSR, TRG1a/b) as assessed according to the Becker criteria in the sampled regional lymph nodes	HIST	F, PP	DA, CI	HISTPATRESLN
Pathological regression for all response categories in the sampled regional lymph nodes	HIST	F, PP	DA	HISTPATRESLN
Perioperative morbidity rate	SURG	F, PP	DA, CI	SURGCOM
Perioperative mortality	SURG	F, PP	DA, CI	SURGEXLEYN
Feasibility rate	EOT	F, PP	DA, CI	EOT_REAS
**TOXICITY / SAFETY**				
Total number of AEs and SAEs, fatal SAEs, and ECIs (total and by grade)	AE	S	DA	AE_TERM, AE_SER, AE_GR, AE_ECI
Total number of AEs, ECIs and SAEs related to study treatment (total and by grade)	AE	S	DA	AE_TERM, AE_SER, AE_ECI, AE_GR, AE_PEMREL, AE_TRAREL, AE_OXREL, AE_FOLREL, AE_5FUREL, AE_DOCREL
Rate of patients with AEs, SAEs, ECIs, and fatal SAEs	AE	S	DA	AE_TERM, AE_SER, AE_ECI, AE_OUT =5

PARAMETER / ANALYSIS DESCRIPTION	CRF PAGE	ANALYSIS SET	METHODS	COMMENT
Rate of patients with AEs, SAEs, ECIs, and fatal SAEs related to study treatments	AE	S	DA	AE_TERM, AE_SER, AE_ECI AE_PEMREL, AE_TRAREL, AE_OXREL, AE_FOLREL, AE_5FUREL, AE_DOCREL
Rate of patients with AEs and SAEs, and ECIs related to study treatment with grade 3, 4, or 5	AE	S	DA	AE_TERM, AE_SER, AE_ECI, AE_GR AE_PEMREL, AE_TRAREL, AE_OXREL, AE_FOLREL, AE_5FUREL, AE_DOCREL
AEs: maximum CTC severity grade (1-5) by patient and by term for all NCI CTC terms observed	AE	S	DA	AE_TERM, AE_GR
Adverse effects: maximum CTC severity grade (1-5) by patient and by term for all NCI CTC terms observed, but limited to events that are related to study treatmenta	AE	S	DA	AE_TERM, AE_GR where (AE_PEMREL, AE_TRAREL, AE_OXREL, AE_FOLREL, AE_5FUREL, AE_DOCREL) =1
Listing for all serious adverse events including the following data:<br><br>a. System Organ Class/ Category<br>b. Event/ CTC Term<br>c. Number of patients with the AE a least once per Arm<br>d. Number of total occurrence of the AE per Arm<br>e. Number of total occurrence of the AE per Arm related to the treatment (in general, not broken down by individual substances)<br>f. Number of fatal outcomes of the AE per Arm<br>g. Number of fatal outcomes of the AE per Arm related to the treatment (in general, not broken down by individual substances)	AE	S	DA	AE_CAT, AE_TERM, AE_GR, AE_SER, AE_PEMREL, AE_TRAREL, AE_OXREL, AE_FOLREL, AE_5FUREL, AE_DOCREL , AEOUT= 5<br><br>AE_TERM_TXT
Listing for all non-serious adverse events with at least 5% of incidence in at least on arm<br><br>a. System Organ Class/Category<br>b. Event/CTC Term<br>c. Number of patients with the AE a least once per Arm<br>d. Number of total occurrences of the AE per Arm	AE	S	DA	AE_CAT, AE_TERM AE_TERM_TXT
Listing of all adverse events not included in NCI CTC categories	AE	S	LISTING	AE_TERMOTHTXT

PP = per protocol set; F = full analysis set; S = safety analysis set; DA = descriptive analysis; HIS = histogram; T = significance test, CI = confidence interval, HR = Hazard Ratio, OR = Odds Ratio

Accompanying research project as described in section 8 of the study protocol is not included in this plan.

A decision on additional, optional analyses to be performed will be based on the results in the report described above. On publication or presentation, they must be clearly described as "post-hoc".

5. TIMING OF ANALYSIS AND INTERIM ANALYSIS

There is no full interim analysis planned for this study, due to the small sample size and the relatively short recruitment period. However, as stated in the protocol, single objectives may be analyzed as soon as sufficient events are available for analysis.

Each co-primary endpoint will be analyzed as soon as the required events are complete. Therefore, the pCR co-primary endpoint will be analyzed once all enrolled patients have obtained the result from pathological response after surgery. The 2-year DFS rate will be assessed once all in-study patients have achieved the 24-month follow-up landmark.

Prior to analysis, we will perform data verification with respect to completeness and plausibility (data cleaning). Inconsistencies and mistakes will be clarified with the study sites and will be removed. The data cleaning process starts soon after first patients are enrolled and monitored. Major protocol violations and special cases will be listed. Finally, a pre-analysis meeting will take place prior to database lock.

The final analysis will be performed on data transferred to a database file indicating the exported date in its file name, having been documented as meeting the cleaning and approval requirements of relevant data management and statistics SOPs and after the finalization and approval of this SAP document.

6. PRE-ANALYSIS MEETING(S)

Before performing any analyses of clinical data collected within this study, one or more pre-analysis meeting(s) are held (cf. ICH E9 Guideline, section 7.1), focusing on the following topics:

- Assessment of patients with major protocol violations (e.g.: with severe violation of in/exclusion criteria before randomization, with unauthorized treatment before reaching a primary endpoint event etc.), with respect to their allocation to analysis sets and/or their exclusion from analyses and/or their primary endpoint event categorization. Lists of such patients/violations are provided by Monitoring / Data Management.

- Amendments to this SAP, which may be required by, e.g., protocol amendments, the actual course of study enrolment and treatment, or important new information from outside the trial.

- Additional definitions eventually required for specific items of the analysis.

Participants of the meeting will include at least the LKP, representatives of the Sponsor and persons responsible for the trial coordination / data management, and the statistical analysis. Whenever possible, the review and decisions will be performed blinded with respect to randomization arm. Of note, the term meeting does not necessarily refer to a face-to-face meeting but also includes virtual meetings via e-mail or telephone conference.

7. SOFTWARE

Statistical analyses will be performed using SAS software version 9.4 or higher (Copyright © 2016 SAS Institute Inc. SAS and all other SAS Institute Inc. product or service names are registered trademarks or trademarks of SAS Institute Inc., Cary, NC, USA) or R version 3.6.1 or higher (Copyright © 2018 the R Foundation for Statistical Computing; Vienna, Austria).

8. References

1. Hofheinz, RD *et al.* 2021. Trastuzumab in combination with 5-fluorouracil, leucovorin, oxaliplatin and docetaxel as perioperative treatment for patients with human epidermal growth factor receptor 2-positive locally advanced esophagogastric adenocarcinoma: A phase II trial of the Arbeitsgemeinschaft Internistische Onkologie Gastric Cancer Study Group. *International journal of cancer* **149:**1322-31

2. Hofheinz, RD *et al.* 2020. Perioperative trastuzumab and pertuzumab in combination with FLOT versus FLOT alone for HER2-positive resectable esophagogastric adenocarcinoma: Final results of the PETRARCA multicenter randomized phase II trial of the AIO. *Journal of Clinical Oncology* **38:**4502-

3. Kaplan, E.L. and Meier, P. (1958) Nonparametric Estimation from Incomplete Observations. Journal of the American Statistical Association, 53, 457-481.

9. Signatures

We the undersigned, certify that we read this SAP and approve it as adequate in scope of the main-analyses of the PHERFLOT study.

Author

[redacted] ....

Date Disorn Sookthai

Coordinating Investigator

[redacted] ....

Date Dr. med. Eray Gökkurt

Project Manager at the sponsor

[redacted] ....

Date Sabine Junge

APPENDIX 1

Description of eCRF forms and (ordered by) corresponding data table short name

eCRF Form / Data table name according to data description by data record table (DRT)	Short code of table(s)
Adverse Events	*AE*
Tumor anamnesis	*ANAM*
Laboratory units	*CENTRE*
Patient information	*CN*
Centers	*CTR*
Demography and informed consent	*DM*
Concomitant medication	*ECM, CM*
Further anticancer therapy	*EFU_TER, FU_THER*
Medical history	*EMH, MH*
End of Treatment	*EOT*
End of Study	*EOS*
Re-surgery	*ERESUG*
Individual Follow-Up	*FU*
Histology	*HIST*
Inclusion / Exclusion Criteria	*IE*
Laboratory Parameters	*LABOR*
Physical Examination	*PE*
Reportable safety events	*RAE*
Surgery	*SURG*
Tumor assessment according to RECIST1.1	*TA*
Visit plan	*VP*
Translational Research	*TLR*
