# Supplementary material for: Perioperative pembrolizumab, trastuzumab and FLOT in HER2-positive localized esophagogastric adenocarcinoma: a phase 2 trial
Source: Nat Med. 2025 Oct 18;31(12):4197–204. doi: 10.1038/s41591-025-03979-y (PMC12705456; doi:10.1038/s41591-025-03979-y)
Supplement: Supplementary file 2 — Reporting Summary [file 41591_2025_3979_MOESM2_ESM.pdf]

nature portfolio

Corresponding author(s): Joseph Tintelnot

Last updated by author(s): 8/24/2025

Reporting Summary

Nature Portfolio wishes to improve the reproducibility of the work that we publish. This form provides structure for consistency and transparency in reporting. For further information on Nature Portfolio policies, see our Editorial Policies and the Editorial Policy Checklist.

Statistics

For all statistical analyses, confirm that the following items are present in the figure legend, table legend, main text, or Methods section.

n/a	Confirmed	
☐	☒	The exact sample size (*n*) for each experimental group/condition, given as a discrete number and unit of measurement
☐	☒	A statement on whether measurements were taken from distinct samples or whether the same sample was measured repeatedly
☐	☒	The statistical test(s) used AND whether they are one- or two-sided<br>*Only common tests should be described solely by name; describe more complex techniques in the Methods section.*
☐	☒	A description of all covariates tested
☒	☐	A description of any assumptions or corrections, such as tests of normality and adjustment for multiple comparisons
☐	☒	A full description of the statistical parameters including central tendency (e.g. means) or other basic estimates (e.g. regression coefficient) AND variation (e.g. standard deviation) or associated estimates of uncertainty (e.g. confidence intervals)
☒	☐	For null hypothesis testing, the test statistic (e.g. *F*, *t*, *r*) with confidence intervals, effect sizes, degrees of freedom and *P* value noted<br>*Give P values as exact values whenever suitable.*
☒	☐	For Bayesian analysis, information on the choice of priors and Markov chain Monte Carlo settings
☒	☐	For hierarchical and complex designs, identification of the appropriate level for tests and full reporting of outcomes
☒	☐	Estimates of effect sizes (e.g. Cohen's *d*, Pearson's *r*), indicating how they were calculated

Our web collection on statistics for biologists contains articles on many of the points above.

Software and code

Policy information about availability of computer code

Data collection	A browser-based EDC system for electronic case report forms (eCRFs), hosted and overseen by the IKF.
Data analysis	Prism version 9.5.1., Illustrator 25.3.1, SAS software version 9.4 or higher and R version 3.6.1 or higher.

For manuscripts utilizing custom algorithms or software that are central to the research but not yet described in published literature, software must be made available to editors and reviewers. We strongly encourage code deposition in a community repository (e.g. GitHub). See the Nature Portfolio guidelines for submitting code & software for further information.

Data

Policy information about availability of data

All manuscripts must include a data availability statement. This statement should provide the following information, where applicable:

- Accession codes, unique identifiers, or web links for publicly available datasets
- A description of any restrictions on data availability
- For clinical datasets or third party data, please ensure that the statement adheres to our policy

Data generated or analyzed during the preparation of this publication are included in this published article and its Extended Data or Supplementary Information. Anonymized individual participant data and related documents can be made available by the corresponding author upon request. Responses to such requests can be expected within onemonth. The trial protocol is available in the Supplementary Information.

Research involving human participants, their data, or biological material

Policy information about studies with human participants or human data. See also policy information about sex, gender (identity/presentation), and sexual orientation and race, ethnicity and racism.

Reporting on sex and gender	The sex of the participants was reported based on self-identification. Gender information was not collected.
Reporting on race, ethnicity, or other socially relevant groupings	Since the trial was conducted at cancer centers in Germany, an ethnic bias in the study population is expected. However, no ethnic group was excluded from participation. Ethnicity was self-reported by the participants.
Population characteristics	Patient as well as tumor characteristics are reported in table 1 of the manuscript.<br>Age, median (Range) 65 (33-76)<br>Sex f/m (%) 6/25 (19.4/80.6)
Recruitment	Recruitment was done at 11 German cancer centers. Recruitment was carried out by 11 PIs and local physicians according to the inclusion criteria, thereby reducing selection bias.
Ethics oversight	The protocol (AIO STO 0321) is approved by the independent ethics committee of the medical council Hamburg.

Note that full information on the approval of the study protocol must also be provided in the manuscript.

Field-specific reporting

Please select the one below that is the best fit for your research. If you are not sure, read the appropriate sections before making your selection.

☒ Life sciences ☐ Behavioural & social sciences ☐ Ecological, evolutionary & environmental sciences

For a reference copy of the document with all sections, see nature.com/documents/nr-reporting-summary-flat.pdf

Life sciences study design

All studies must disclose on these points even when the disclosure is negative.

Sample size	For DFSR@2, we tested the null hypothesis of ≤50% against the alternative of ≥70% using a one-sided α of 0.10 and 80% power in a Fleming single-stage design. For the pCR rate, the null hypothesis of ≤12% versus an alternative of ≥30% was tested with a one-sided α of 0.05 and 80% power. Both calculations required 27 evaluable patients; allowing for a 10% dropout yielded a planned enrollment of 30 patients.
Data exclusions	No data was excluded.
Replication	Not applicable.
Randomization	Not applicable.
Blinding	Not applicable.

Reporting for specific materials, systems and methods

We require information from authors about some types of materials, experimental systems and methods used in many studies. Here, indicate whether each material, system or method listed is relevant to your study. If you are not sure if a list item applies to your research, read the appropriate section before selecting a response.

Materials & experimental systems

n/a	Involved in the study	
☐	☒	Antibodies
☒	☐	Eukaryotic cell lines
☒	☐	Palaeontology and archaeology
☒	☐	Animals and other organisms
☐	☒	Clinical data
☒	☐	Dual use research of concern
☒	☐	Plants

Methods

n/a	Involved in the study	
☒	☐	ChIP-seq
☒	☐	Flow cytometry
☒	☐	MRI-based neuroimaging

Antibodies

Antibodies used	IHC was performed on 2-µm paraffin sections using the Ventana Benchmark XT automated staining system (Ventana Medical Systems). The following antibodies were used at the manufacturer's ready-to-use concentrations: PD-L1 (Ventana, clone SP263; catalog no. 790-4905), MLH1 (Ventana, clone M1; catalog no. 760-5091), PMS2 (Ventana, clone A16-4; catalog no. 760-5094), MSH2 (Ventana, clone G219-1129; catalog no. 760-5093), and MSH6 (Ventana, clone SP93; catalog no. 760-5092).
Validation	Pathological assessments were performed according to local routines accepted as clinical standards. For central testing, staining was performed according to the manufacturer's standard for CPS (https://elabdoc-prod.roche.com/eLD/web/global/en/products/RTD001234) and dMMR (https://www.accessdata.fda.gov/cdrh_docs/pdf21/P210001d.pdf).

Clinical data

Policy information about clinical studies

All manuscripts should comply with the ICMJE guidelines for publication of clinical research and a completed CONSORT checklist must be included with all submissions.

Clinical trial registration	NCT05504720 (clinicaltrials.gov) and 2024-513610-34-00 (euclinicaltrials.eu).
Study protocol	Can be found within the Supplementary Information.
Data collection	Data was collected centrally at the IKF (sponsor and CRO or the trial).
Outcomes	The study's co primary endpoints are the two year disease free survival rate (DFSR@2) and pCR rate. For DFSR@2, the null hypothesis of P≤50% against the alternative of P≥70% will be tested using a one sided α of 0.10 and 80% power in a Fleming single stage design. For the pCR rate, the null hypothesis of P≤12% versus an alternative of P≥30% was tested with a one sided α of 0.05 and 80% power. Both calculations required 27 evaluable patients; accounting for a 10% dropout rate, the planned enrollment was set at 30 patients. Secondary endpoints - including overall response, R0 resection, overall survival, Becker regression grading (TRG1a/b), perioperative morbidity and mortality, feasibility rate and safety (AEs per NCI CTC v5.0) - are analyzed descriptively, with time to event outcomes estimated by Kaplan - Meier methods. Calculations were performed on the data actually available. Incomplete time-to-event observations were handled as censored measurements, and missing data for the primary endpoint were considered failures. In this interim analysis, only the pCR rate is reported because the DFSR@2 data are not yet mature. Regarding the secondary endpoints, the R0 resection rate, safety and toxicity, and feasibility are reported, as survival data are still immature and ORR data are not yet available. The trial protocol and statistical analysis plan are available in the Supplementary Information.

Plants

Seed stocks	–
Novel plant genotypes	–
Authentication	–
